# A GPS assisted translocation experiment to study the homing behavior of red deer

**DOI:** 10.1038/s41598-024-56951-0

**Published:** 2024-03-21

**Authors:** Václav Silovský, Lukas Landler, Monika Faltusová, Luca Börger, Hynek Burda, Mark Holton, Ondřej Lagner, Erich Pascal Malkemper, Astrid Olejarz, Magdalena Spießberger, Adam Váchal, Miloš Ježek

**Affiliations:** 1 Department of Game Management and Wildlife Biology, Faculty of Forestry and Wood Sciences, Czech University of Life Sciences Prague, Prague, Czech Republic; 2 Institute of Zoology, University of Natural Resources and Life Sciences (BOKU), Gregor-Mendel-Straße 33/I, 1180 Vienna, Austria; 3 Swansea Lab for Animal Movement, Department of Biosciences, Swansea University, Singleton Park, Swansea, Wales SA2 8PP UK; 4 Department of Spatial Sciences, Faculty of Environmental Sciences, Czech University of Life Sciences Prague, Prague, Czech Republic; 5 Research Group Neurobiology of Magnetoreception, Max Planck Institute for Neurobiology of Behavior – Caesar, Ludwig-Erhard-Allee 2, 53175 Bonn, Germany

**Keywords:** Mammal navigation, Satellite tracking, *Cervus elaphus*, Czech Republic, Spatial orientation, Animal behaviour, Animal migration

## Abstract

Many animals return to their home areas (i.e., ‘homing’) after translocation to sites further away. Such translocations have traditionally been used in behavioral ecology to understand the orientation and migration behavior of animals. The movement itself can then be followed by marking and recapturing animals or by tracking, for example, using GPS systems. Most detailed studies investigating this behavior have been conducted in smaller vertebrates (e.g., birds, amphibians, and mice), whereas information on larger mammals, such as red deer, is sparse. We conducted GPS-assisted translocation experiments with red deer at two sites in the Czech Republic. Individuals were translocated over a distance of approximately 11 km and their home journey was tracked. Circular statistics were used to test for significant homeward orientation at distances of 100, 500, 1000, and 5000 m from the release site. In addition, we applied Lavielle trajectory segmentation to identify the different phases of homing behavior. Thirty-one out of 35 translocations resulted in successful homing, with a median time of 4.75 days (range 1.23–100 days). Animals were significantly oriented towards home immediately after release and again when they came closer to home; however, they did not show a significant orientation at the distances in between. We were able to identify three homing phases, an initial ‘exploratory phase’, followed by a ‘homing phase’ which sometimes was again followed by an ‘arrival phase’. The ‘homing phase’ was characterized by the straightest paths and fastest movements. However, the variation between translocation events was considerable. We showed good homing abilities of red deer after translocation. Our results demonstrate the feasibility of conducting experiments with environmental manipulations (e.g., to impede the use of sensory cues) close to the release site. The homing behavior of red deer is comparable to that of other species, and might represent general homing behavior patterns in animals. Follow-up studies should further dissect and investigate the drivers of the individual variations observed and try to identify the sensory cues used during homing.

## Introduction

Animal navigation, spatial cognition, and memory are essential abilities that allow animals to reach specific destinations, such as food resources, mating grounds, and nesting sites, and exploit spatially clustered resources efficiently^1,2^. Many species display site fidelity, that is, they return to specific locations every season or stay in the same general area year around^3^. Typically, animals that show site fidelity return to their approximate capture location after translocation to a site outside their typical residence^4,5^. The reason for such a preference for certain ‘home areas’ can be manifold, but is often associated with territorial behavior and/or mating sites (for example^6,7^) or spatial learning and memory-based home range formation^8,9^. The return behavior to such specific sites is called ‘homing’ and has been extensively studied in smaller terrestrial vertebrate species such as birds^10,11^ and, to a lesser extent, amphibians^12^, mice^13–15^, rabbits^16,17^, bats^18^, and dogs^19^. Large terrestrial mammals also show good homing abilities^20^, and some perform seasonal migrations (for example^21^). However, spatial movements of large non-migratory mammals have been subjected to far fewer experimental/manipulative investigations, probably due to practical issues with their translocation.

Studies across many species suggest that there are species-specific limits of translocation distances beyond which animals are no longer able to navigate back^20,22^. These limitations might be associated with the navigational cues used for homing, which might require familiarity with the environment. Homing abilities outside the previously visited (i.e. ‘familiar’) area are often termed ‘true navigation’, meaning that the animals can extrapolate the navigation cues beyond their home range. It is unknown what kinds of sensory mechanisms large terrestrial mammals use for their navigation performance. However, in smaller mammals it has been shown that olfactory^23^, visual^24^ and magnetic cues^25^ can be used for orientation. In principle, larger mammals, such as the red deer (*Cervus elaphus*), can use all of these cues, if available. However, no direct tests of the cues involved have yet been performed.

Understanding the homing abilities of larger mammals is important when managing populations, either for the protection of endangered animals or for game management. For instance, bears are sometimes relocated to avoid conflict with human settlements, which is considered a better alternative to sacrificing animals^26^. However, such efforts are unsuccessful if individuals return to their home sites. Bears can find their way home from up to 100 km^26^, which is likely an especially well-developed homing ability for large terrestrial mammals. For white-tailed deer (*Odocoileus virginianus*), studies on homing performance showed frequent settlement of the animals in an area approximately 30 km from the release site^20^. For instance, in a study in Illinois of 28 translocated individuals (distance 10–58 km), only two returned home^27^. Such studies usually rely on mark-recapture or radio telemetry, while advancements in remote sensing allow detailed movement monitoring of animals over long periods due to GPS-assisted tracking. Translocation studies with red deer are still missing, although they show high site fidelity for their home ranges, regardless of whether they show seasonal migration between home ranges or whether they remain resident in an area during the year^28,29^. High site fidelity could lead to issues when managing populations because translocations that may be intended to resettle animals can fail if they show homing. In contrast, well-developed homing abilities may provide us with a study system that can be used to investigate navigational cues in larger mammals. This might reveal similarities and differences in cue use and integration based on the scale of migration, for example, a few hundred meters for mice or a few kilometers for deer.

In the current study, we performed translocation experiments with GPS-monitored red deer, followed by detailed analyses of the homing performance and tracks. We show overall consistent and fast homing behavior, with most animals returning within days from a 10 km distance.

## Methods

### Study site and species

**Figure 1:**
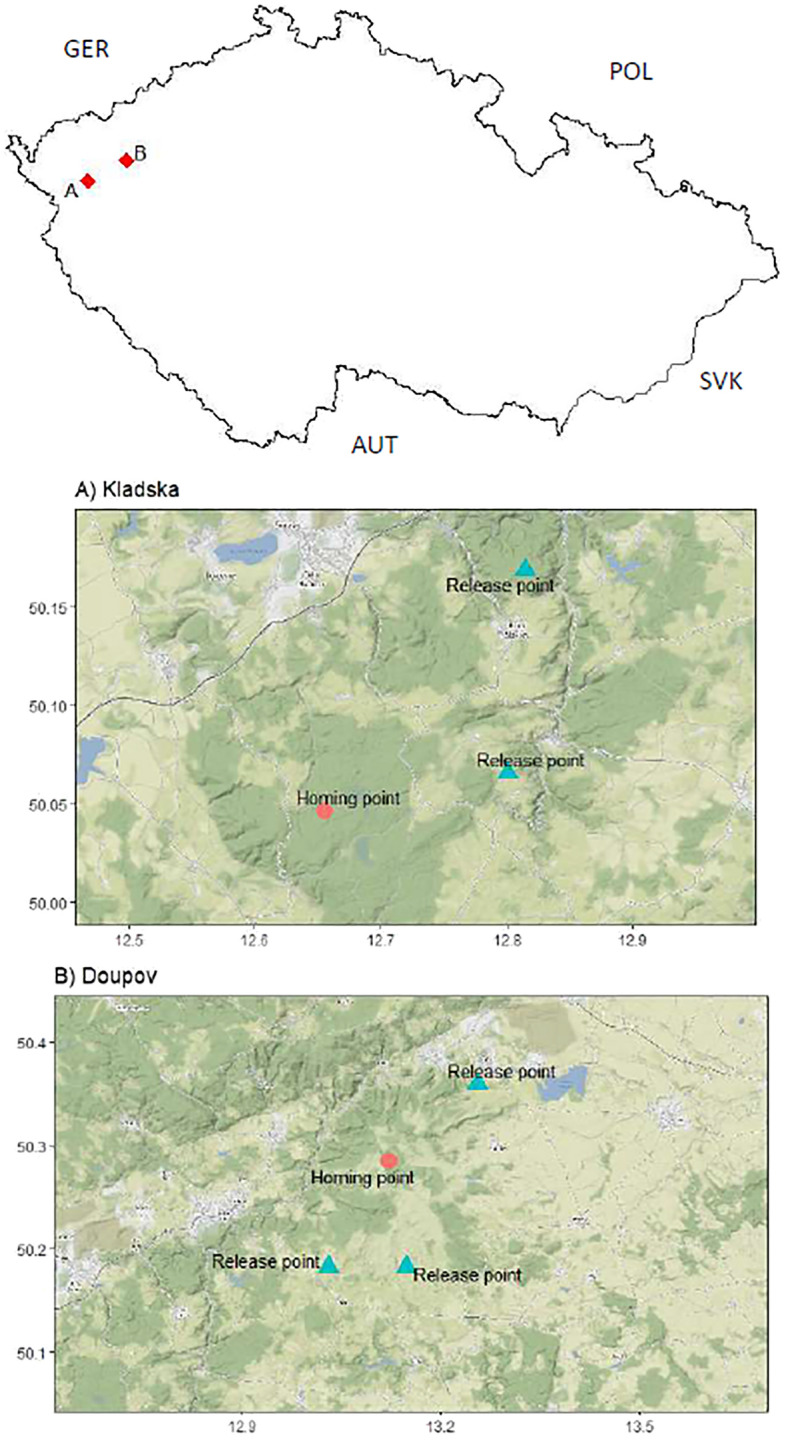
Map with all release sites and respective goals (homes) in Location Kladska (**A**) and Doupov (**B**)^45^.

Translocation experiments with red deer (*Cervus elaphus*) were conducted on two different populations in 2019, 2020, and 2021 in the Czech Republic (Fig. 1) at two different locations in West Bohemia. The first was in the Doupov Mountains, a military training site that is closed to the public and used only for military training, game species hunting, and forestry. This area spans 331 km^2^. The second location was Kladska, which is situated in the west and consists of a semi-natural coniferous forest. The total area is 140 km^2^ and forest stands are used for timber production and recreation. Stable deer populations are present at both locations and both are managed by hunting. In total, we translocated 23 individuals at least once (15 animals in Doupov and eight animals in Kladska). Thirteen animals were translocated a second time and two animals were translocated a third time. Two animals were excluded from the analysis: one was killed by wolfs one day after translocation, and for another one the tracking device was malfunctioning. One animal was male, and the others were adult females. Translocations took place during early spring from February to April, which is the spring season without large-scale movements, while the rutting season starts in mid-September and continues until mid-October.

### Translocation procedure

Animals were anesthetized using a mixture of Ketamine and Xylazine (3 ml per 100 kg of body mass). The concentrations of ketamine and xylazine were 50 mg/ml and 250 mg/ml, respectively. The animals were then secured in a wooden transport box on the trailer behind a pick-up truck and driven to the release sites (11.9 ± 1.8 km distance), which were either approximately towards the north (n = 4 + 2), south (n = 15 + 7), or west (n = 8) of the capture location and 12 km away. All individuals were transported in a wooden box developed for animal transport. The wooden box used for transport included ventilation holes in the lower part of the construction. The animals were unable to see the outside of the box during transport. The translocation time was less than 90 min. The animals were released from the box after translocation and visually checked by the veterinary staff. All individuals left the box without injury, and none needed an antidote.

### GPS tracking

**Figure 2:**
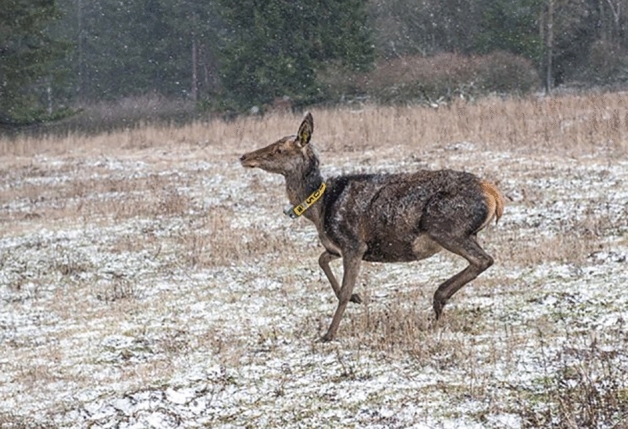
Translocated female red deer (Animal ID 105) were equipped with a GPS collar.

Red deer individuals were tracked using GPS Vertex Plus collars (Vectronic Aerospace GmbH) (Fig. 2). The total mass of the collars was 750 g, and their lifetime was approximately 1 year. The collar mass was < 3% of the animal’s body mass ^46^. GPS data were retrieved via GSM every 8 h and stored on servers. The frequency of GPS fixes was set to 30 min intervals and the collars included a VHF drop-off mechanism (Vectronic Aerospace GmbH) for non-invasive collar release after the end of the project or before the end of the battery lifetime.

### Track analysis

For the aim of this study, we defined all animals that reached or crossed a circle of 1 km radius around their capture location within 180 days after translocation as “homed successfully” and animals that did not reach this criterion as “did not home”. This does not necessarily mean that animals defined as “did not home” did not home at a later time point.

For all animals (“all together”, “homed successfully” and “did not home”) we calculated the homeward bearings at a distance of (at least) 100 m, 500 m, 1 km and 5 km from the release location. The bearings were calculated as the angle of the vector between the release location and the closest GPS fix after individuals crossed the respective distance mark. For this analysis, we used the recently described MANOVA approach^30^. We used the animals’ ID as a random error term (intercept) in the mixed-effects MANOVA model to account for the repeated testing of individuals (we only applied this approach to distributions with at least five bearings). This approach was used to circumvent non-independence issues caused by animal retesting. Our aim was to test whether animals orient more strongly towards home with increasing distance from the release location. The idea behind this analysis was that a deer might, at first, disperse in a random direction when released after re-orienting itself and then following a homeward direction. Circular plots were generated using the wrapper function presented in Pail et al.^31^, which is based on the plot.circular function from the circular package in R^32^.

To analyze the different homing segments for all deer that homed successfully, we used the approach described by Lavielle^33,34^, using the function *lavielle.ltraj* from the R package adehabitatLT^35^. In this approach, a track is partitioned using a contrast function, in which we set the minimum length of a segment to four, used four as the maximum possible segments, and set the type of segmentation to “mean”, following the suggestions by Calenge^36^. We then applied the *chooseseg* function to determine the optimum number of segments for individual tracks. In the final step, we applied the *findpath* function to divide the tracks into the suggested segments; this was only done if more than one segment was suggested from the *chooseseg* function. For each segment in each track we calculated the average speed, straightness (as the standardized vector length ‘r’) and proportion of entire track. We also correlated the number of days required for homing with the number of trajectory segments using the cor. test function.

### Ethical approval

Red deer trapping was implemented in accordance with the decision of the Ethics Committee of the Ministry of the Environment of the Czech Republic (number MZP/2019/630/361). This study was conducted in compliance with the recommendations of the ARRIVE guidelines. The trapping and handling protocol was approved by the ethics committee of the Ministry of the Environment of the Czech Republic, and we confirm that the experiment was performed in accordance with the relevant guidelines and regulations.

## Results

Of all deer translocations 89% (31 of 35) ended with successful homing, i.e., returned back to the capture location in a vicinity of a 1 km radius within 180 days (see Supplementary Information: Overview_Table_S1.pdf, tracks can be found in Tracks_All_animals.pdf and Tracks_homed.pdf supplementary files). Of the four translocations without homing, two were individuals that had been translocated the second time. Of all individuals, 19 homed at least once, and two did not.

**Figure 3:**
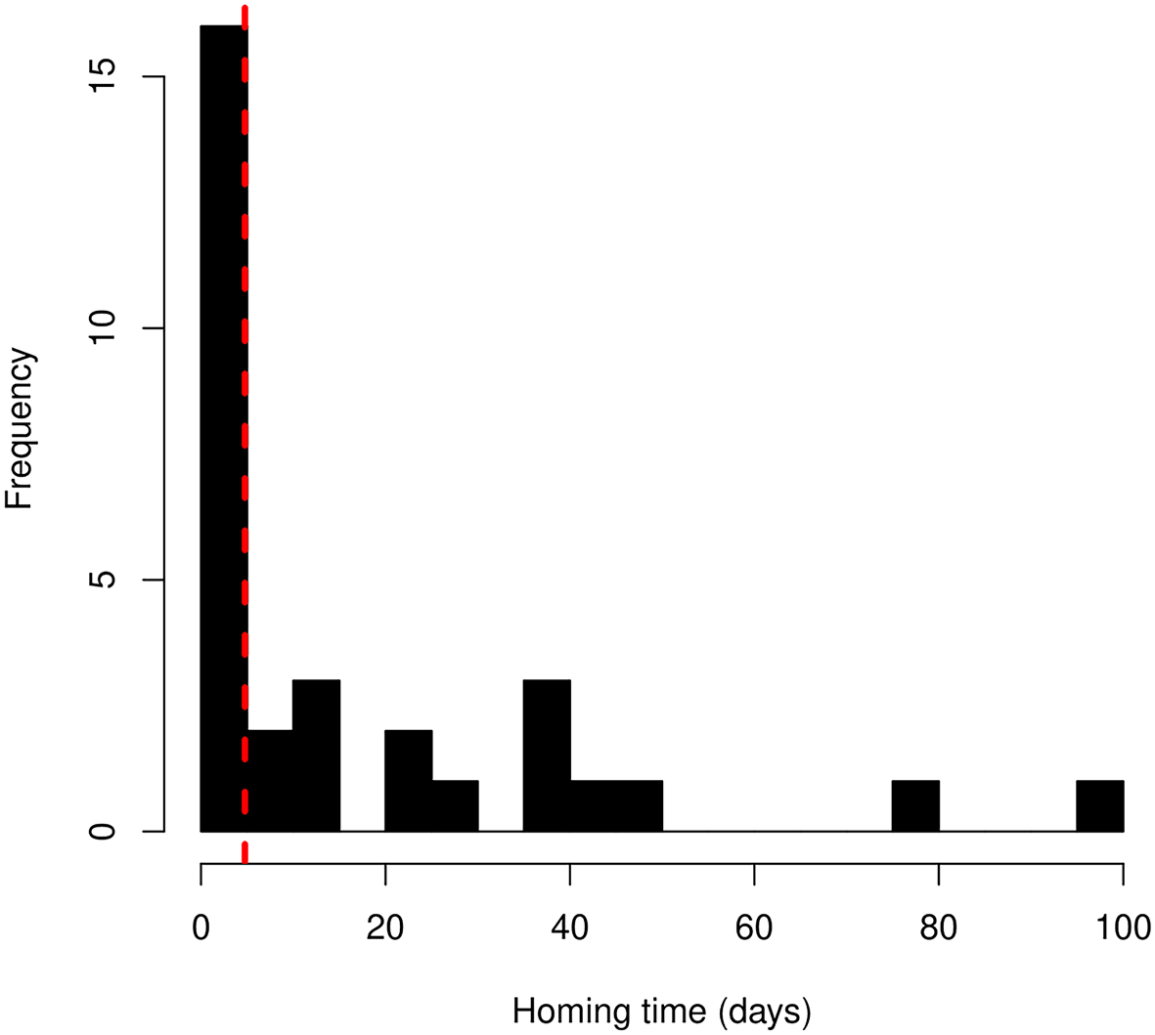
The frequency distribution of deer homing times (in days) is shown in a histogram. The median homing time (4.75 days) is indicated by a red dashed vertical line.

The median homing time was 4.75 days (range 1.23–100 days) (Fig. 3).

**Figure 4:**
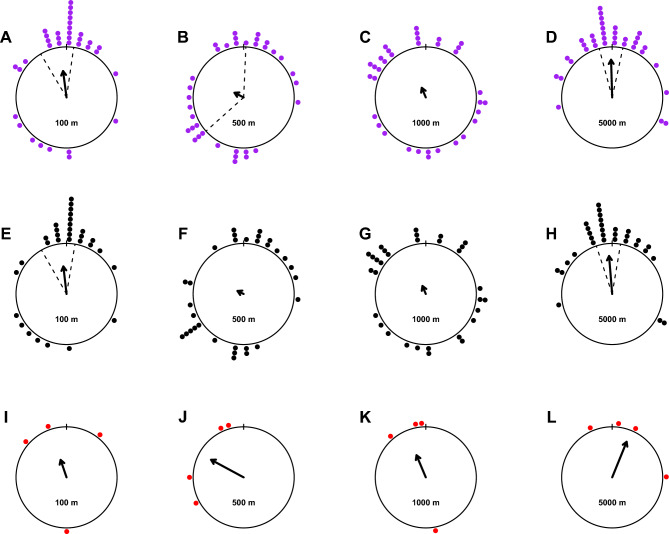
The bearings relative to the goal direction at a radius of 100 m (**A,E,I**), 500 m (**B,F,J**), 1000 m (**C,G,K**), and 5000 m (**D,H,L**) around the release location for all trials (purple dots, **A–D**), trials where animals homed successfully (black dots, **E–H**), and trials where they did not home (red dots, **I–L**). The mean vectors are shown as solid lines with arrowheads, whereas the length corresponds to the r-value of the mean vector. Bootstrap confidence intervals (dashed lines) around the means were calculated for significant distributions according to the MANOVA approach (for all distributions with a minimum sample size of five).

The initial orientation (100 m) of deer that homed successfully was significantly directed towards the home direction (Fig. 4, MANOVA tables in Supplementary Information: MANOVA_results_tables.pdf). The subsequent orientations at distances of 500 m and 1 km from the release site were randomly oriented, whereas at a distance of 5 km, the animals were again oriented towards home. As only four releases were included in the analyses that did not include home, we did not perform a statistical analysis. However, the general patterns, especially for the initial homeward trend, appeared comparable to those of the animals that did home. When taken together, the orientation at a distance of 500 m showed a weakly significant homeward vector (Fig. 4B).

**Figure 5:**
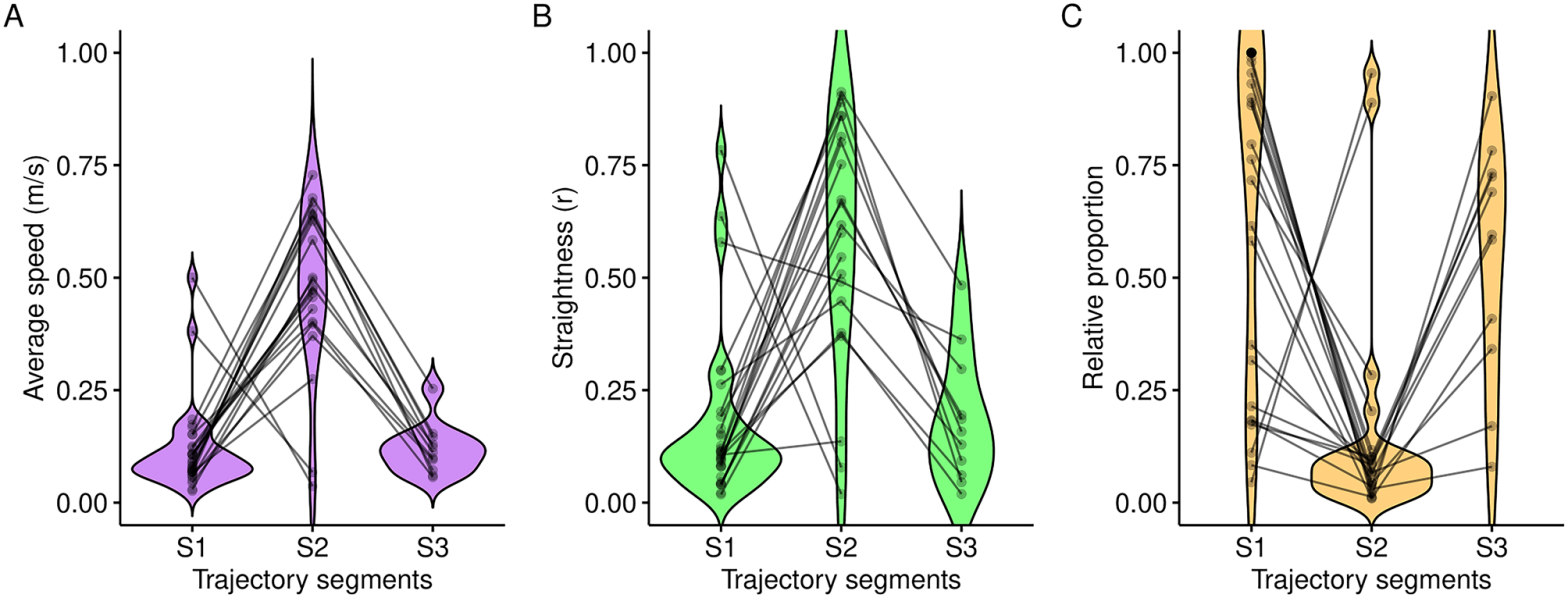
The trajectory segments based on the Lavielle segmentation (Segment 1 to Segment 3, labeled S1 to S3) compared the average speed (**A**), straightness of the tracks (using the standardized vector length r, **B**), and relative proportion of the segments in comparison to the overall trajectories (**C**).

The segmentation algorithm resulted in 10 trajectories that were not split up (i.e., one segment for the entire track, herein assigned to segment 1), 10 trajectories were split into two segments (assigned to segments 1 and 2), and 11 were split into three segments (Fig. 5). None was split into four segments (the highest segmentation possible in our analysis). The general trend for the trajectories showed that the animals had lower average speed and straightness in segment 1 than in segment 2, and lower values in segment 3 (Fig. 5). In contrast, the relative proportion of segments (i.e., the relative proportion of each segment on the entire trajectory) showed the opposite trend (lower proportion in segment 2 than in segments 1 and 3). The two animals with directly opposite trends consisted of only two segments, suggesting that they might belong to segments 2 and 3 compared with the other animals (i.e., missing segment 1). Our results show a possible separation in three phases of homing, which could be classified as an initial exploration of the release site, a faster homing phase, and a (possible) exploration close to the home site, until our criteria of a 1 km radius around the release site were met. The number of days the animals needed home was significantly negatively correlated with the number of segments (Pearson’s product-moment correlation: − 0.45, t =  − 2.7195, df = 29, p-value = 0.011); that is, animals that needed longer were more likely to be classified into fewer segments using our segmentation algorithm.

## Discussion

Our study showed the excellent homing abilities of red deer when translocated by approximately 11 km. This is surprising, as the selected release sites may be beyond the home ranges of the investigated deer, suggesting that the individuals likely navigated homewards from a novel location. Interestingly, the initial orientation at the release sites was significantly oriented towards the animals’ homes. This may suggest good navigational capabilities, potentially based on a global map cue, such as a magnetic or olfactory internal map^37,38^. Alternatively, deer may have a very large area of familiarity and used familiar landmarks (e.g., visual cues) to orient towards home or perceived some salient visual or olfactory cues directly from the home area. While we cannot exclude the possibility that deer path-integrated the way back to their home location, using the information they memorized during the translocation, this is highly unlikely. They were anesthetized during at least part of the translocation and could not see out of the transport box while being transported at higher speeds that they usually experience.

After this initial orientation, the animals appeared to explore their release areas before starting their journey home. This is again exemplified by the highly significant homeward orientation at a distance of 5 km from the release site. Overall, the homing abilities of red deer appear to be better than those of white-tailed deer, at least according to the available literature, where the latter species failed to return from comparable distances in many cases (c.f.^20^).

Despite considerable variation, homeward navigation contains repeatable features among some individuals. There often was a first ‘exploratory phase’ immediately after release which is recognizable by its more irregular and slower movement. The animals then started the ‘homing phase’, and these trajectories were characterized by straight and fast movements. Close to home, there often was a final ‘arrival phase’ where the animals are again moving slower and less straight. Typically, the homing phases are shorter than those of the other two (first and third segments). Two animals did not show the initial exploration phase and started immediately with the homing phase, and 10 animals did not show discrete phases according to our classification algorithm. The arrival phase appeared in 11 of the animals and might only be entered when the animals miss the goal or if they assume they have reached ‘home’. The longer the animals needed to reach home, the fewer phases the classification algorithm detected. This is possibly because they never entered the homing phase and slowly wandered towards home, never transitioning into a fast and straight movement.

Interestingly, our results are comparable to pigeon homing analyses, which also showed an initial phase with an individual variable duration and a homing phase with steeply increased steadiness (i.e., straightness)^39^. This suggests a common mechanism underlying the navigational process, presumably related to the cues used for homing, as well as the individuals’ motivational state. In pigeons, studies have shown the importance of the hippocampus in navigational tasks^40–42^, a brain structure that is also important in mammal navigation^43^. It is very likely that further investigation would find comparable neuronal processes underlying these strikingly similar behavioral patterns.

It would be highly interesting to gain further insights into the specific behavior of the animals during the identified homing phases. Furthermore, exploring what happens when they do not home could provide insights into the navigation cues that deer use. For instance, electromagnetic anomalies, obscured sun (cloud cover), or wind from directions other than home (lacking home odors) could interfere with homing abilities and either delay homing or impede homing during the time interval we considered homing. Manipulating the sensory environments of the release sites could be an option for discerning the sensory mechanisms used for homing (e.g., radio-frequency noise to disrupt the magnetic sense (c.f.^44^)). This is possible because the initial orientation of the animals corresponded well with their direction towards home. In conclusion, we provide evidence for the excellent homing abilities of red deer, which fits well with the homing behavior found in other animals (e.g., well-studied pigeons). Further studies regarding sensory cues and behavioral switches along the route would be a fruitful endeavor for the future.

### Supplementary Information

Supplementary Information 1.

Supplementary Information 2.

Supplementary Information 3.

Supplementary Information 4.

## Data Availability

The datasets used and analyzed during the current study are available from the corresponding author upon reasonable request.
