# Supplementary material for: A GPS assisted translocation experiment to study the homing behavior of red deer
Source: Sci Rep. 2024 Mar 21;14:6770. doi: 10.1038/s41598-024-56951-0 (PMC10958021; doi:10.1038/s41598-024-56951-0)
Supplement: Supplementary file 1 — Supplementary Information 1. [file 41598_2024_56951_MOESM1_ESM.pdf]

MANOVA result tables (R output of manova function)

As described in Landler et al. (2022, Movement Ecology):
the intercept in the model corresponds to a significance of departure from uniformity.

100 m - all animals

```
Error: ID
          Df  Pillai approx F num Df den Df    Pr(>F)
(Intercept)  1 0.54898   11.563      2     19 0.0005186 ***
Residuals   20
---
Signif. codes:  0 ‘***’ 0.001 ‘**’ 0.01 ‘*’ 0.05 ‘.’ 0.1 ‘ ’ 1

Error: Within
        Df Pillai approx F num Df den Df Pr(>F)
Residuals 14
```

500 m - all animals

```
Error: ID
          Df  Pillai approx F num Df den Df  Pr(>F)
(Intercept)  1 0.30557   4.1803      2     19 0.03129 *
Residuals   20
---
Signif. codes:  0 ‘***’ 0.001 ‘**’ 0.01 ‘*’ 0.05 ‘.’ 0.1 ‘ ’ 1

Error: Within
        Df Pillai approx F num Df den Df Pr(>F)
Residuals 14
```

1000 m - all animals

```
Error: ID
          Df  Pillai approx F num Df den Df Pr(>F)
(Intercept)  1 0.10836   1.1545      2     19 0.3364
Residuals   20

Error: Within
        Df Pillai approx F num Df den Df Pr(>F)
Residuals 14
```

5000 m - all animals

```
Error: ID
          Df  Pillai approx F num Df den Df    Pr(>F)
(Intercept)  1 0.84953   53.636      2     19 1.534e-08 ***
Residuals   20
---
Signif. codes:  0 ‘***’ 0.001 ‘**’ 0.01 ‘*’ 0.05 ‘.’ 0.1 ‘ ’ 1

Error: Within
        Df Pillai approx F num Df den Df Pr(>F)
Residuals 14
```

100 m - trials with successful homing

```
Error: ID
          Df  Pillai approx F num Df den Df   Pr(>F)
```

```
(Intercept)  1 0.55223   10.483      2     17 0.001081 **
Residuals   18
---
Signif. codes:  0 ‘***’ 0.001 ‘**’ 0.01 ‘*’ 0.05 ‘.’ 0.1 ‘ ’ 1

Error: Within
          Df Pillai approx F num Df den Df Pr(>F)
Residuals 12
```

500 m - trials with successful homing

```
Error: ID
            Df Pillai approx F num Df den Df Pr(>F)
(Intercept)  1 0.1467   1.4613      2     17 0.2596
Residuals   18

Error: Within
          Df Pillai approx F num Df den Df Pr(>F)
Residuals 12
```

1000 m - trials with successful homing

```
Error: ID
            Df   Pillai approx F num Df den Df Pr(>F)
(Intercept)  1 0.067461   0.6149      2     17 0.5523
Residuals   18

Error: Within
          Df Pillai approx F num Df den Df Pr(>F)
Residuals 12
```

5000 m - trials with successful homing

```
Error: ID
            Df  Pillai approx F num Df den Df    Pr(>F)
(Intercept)  1 0.85548   50.316      2     17 7.234e-08 ***
Residuals   18
---
Signif. codes:  0 ‘***’ 0.001 ‘**’ 0.01 ‘*’ 0.05 ‘.’ 0.1 ‘ ’ 1

Error: Within
          Df Pillai approx F num Df den Df Pr(>F)
Residuals 12
```
