# Supplementary material for: A GPS assisted translocation experiment to study the homing behavior of red deer
Source: Sci Rep. 2024 Mar 21;14:6770. doi: 10.1038/s41598-024-56951-0 (PMC10958021; doi:10.1038/s41598-024-56951-0)
Supplement: Supplementary file 2 — Supplementary Information 2. [file 41598_2024_56951_MOESM2_ESM.pdf]

Supplementary information: Overview_Table_S1 - Detailed information of tested animals and released sites used for homing study analyses.

Animal ID	Sex	Age class	Location	Trial	Date of translocation	Note
96	F	adult	Doupov	first	2020-03-31	
96	F	adult	Doupov	second	2021-04-01	
104	F	adult	Doupov	first	2020-03-13	
104	F	adult	Doupov	second	2021-03-26	
105	F	adult	Kladska	first	2019-03-01	
105	F	adult	Kladska	second	2020-02-03	
107	F	adult	Doupov	first	2019-03-26	
107	F	adult	Doupov	second	2020-03-15	
107	F	adult	Doupov	third	2021-04-06	
108	F	adult	Doupov	first	2019-03-23	
108	F	adult	Doupov	second	2020-03-20	
108	F	adult	Doupov	third	2021-03-22	
110	F	adult	Kladska	first	2019-02-27	
110	F	adult	Kladska	second	2020-01-31	Did not home in 180 days
115	F	adult	Doupov	first	2019-03-23	
115	F	adult	Doupov	second	2020-03-20	
117	F	adult	Doupov	first	2019-03-26	
117	F	adult	Doupov	second	2020-03-08	
118	F	adult	Doupov	first	2020-03-13	
120	F	adult	Doupov	first	2019-03-28	
120	F	adult	Doupov	second	2020-03-23	
124	F	adult	Doupov	first	2020-03-26	Did not home in 180 days
126	F	adult	Kladska	first	2020-02-26	
132	M	2-year-old male	Kladska	first	2020-03-11	
133	F	adult	Doupov	first	2020-02-28	
133	F	adult	Doupov	second	2021-03-26	Did not home in 180 days
134	F	adult	Doupov	first	2020-03-15	Excluded: killed by wolf
135	F	adult	Doupov	first	2020-03-08	
135	F	adult	Doupov	second	2021-03-22	
137	F	adult	Doupov	first	2020-03-06	
137	F	adult	Doupov	second	2021-04-09	
139	F	adult	Doupov	first	2020-03-23	Excluded: malfunction of tracking device
142	F	adult	Kladska	first	2020-01-27	Did not home in 180 days
144	F	adult	Kladska	first	2020-01-24	
145	F	adult	Kladska	first	2020-01-20	
149	F	adult	Kladska	first	2020-01-10	
151	F	adult	Doupov	first	2019-03-28	
