# Supplementary material for: A GPS assisted translocation experiment to study the homing behavior of red deer
Source: Sci Rep. 2024 Mar 21;14:6770. doi: 10.1038/s41598-024-56951-0 (PMC10958021; doi:10.1038/s41598-024-56951-0)
Supplement: Supplementary file 3 — Supplementary Information 3. [file 41598_2024_56951_MOESM3_ESM.pdf]

Map Information

Open purple circle: Home area

turquoise crosshair: Release location

Blue triangle: first tracked position

Red square: last tracked position (or reached home area)

The ID of the animal and testing round are given in the map headers (number before decimal point is the ID number, after is the testing round).

96.1

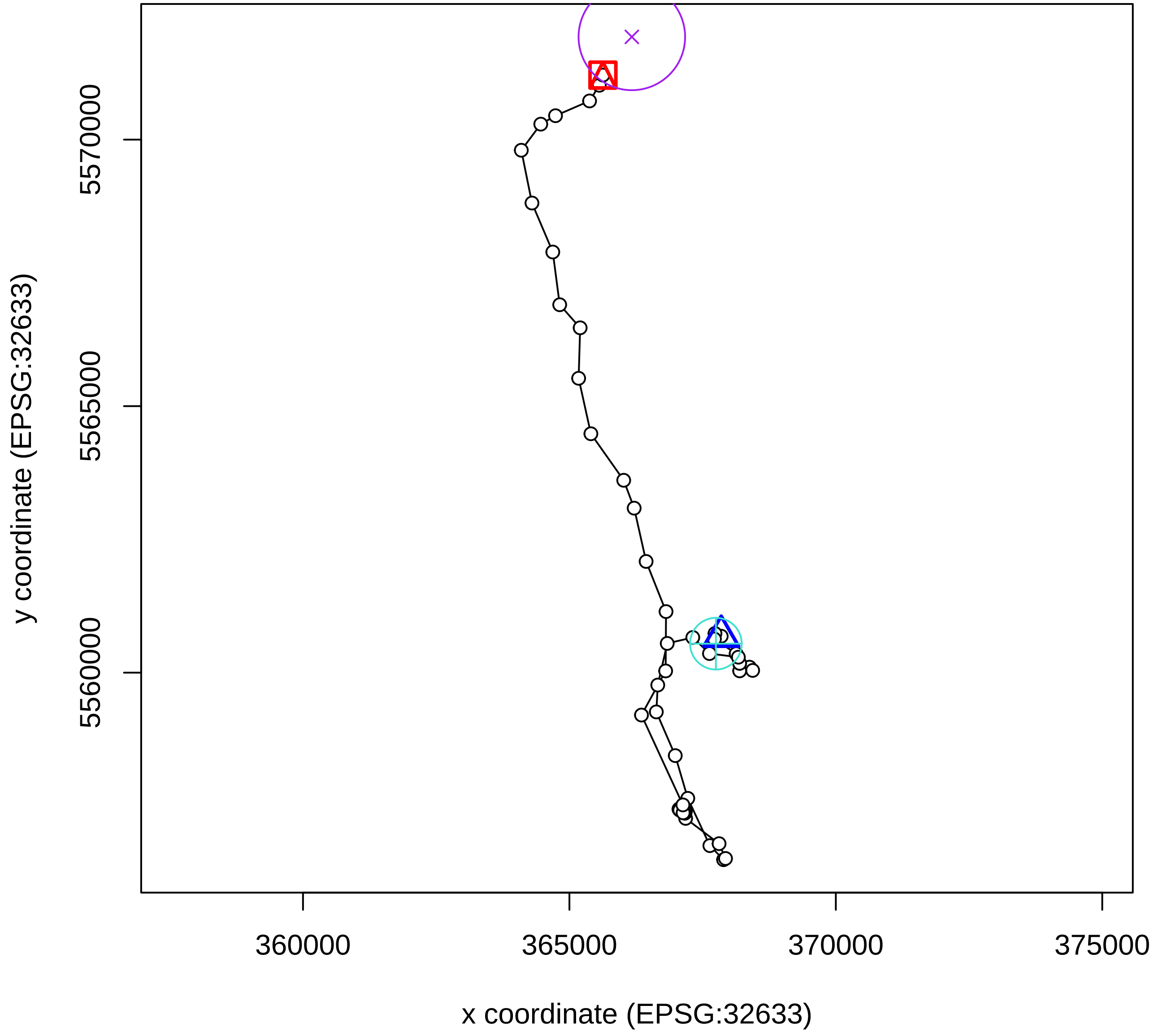

5570000
5565000
5560000
y coordinate (EPSG:32633)
360000
365000
370000
375000
x coordinate (EPSG:32633)

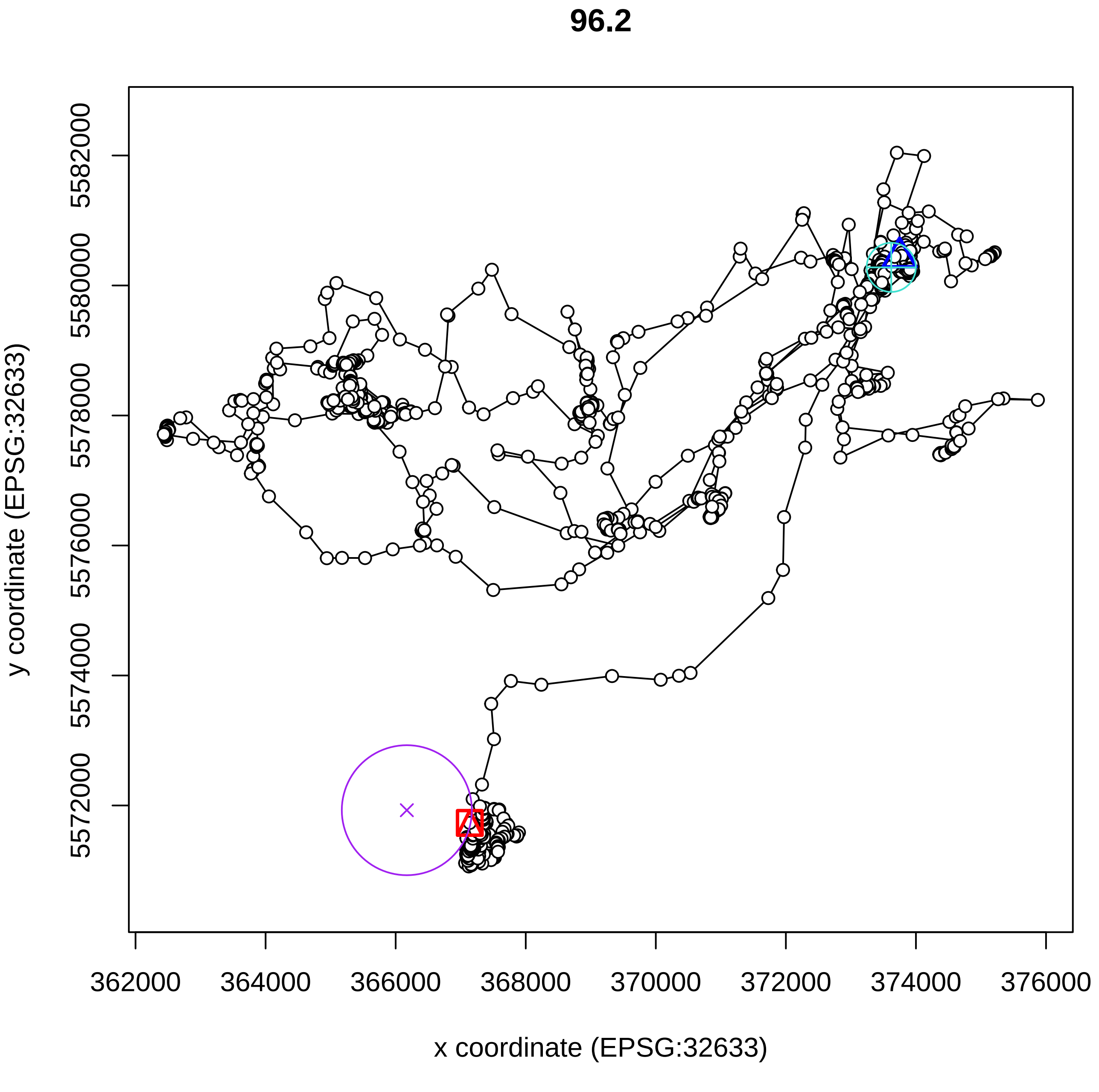

96.2
5572000
5574000
5576000
5578000
5580000
5582000
y coordinate (EPSG:32633)
362000
364000
366000
368000
370000
372000
374000
376000
x coordinate (EPSG:32633)

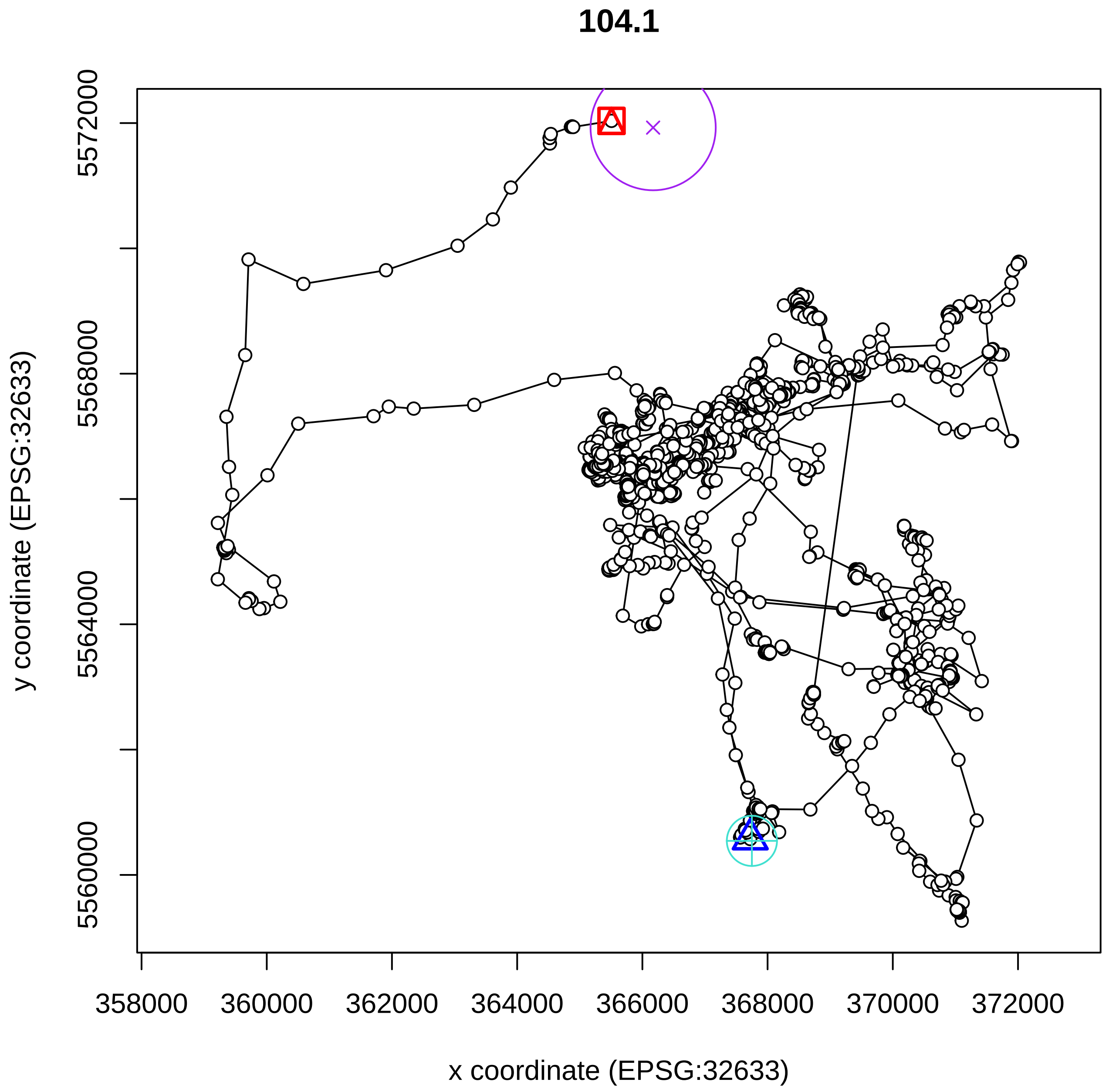

104.1
5572000
5568000
5564000
5560000
y coordinate (EPSG:32633)
358000
360000
362000
364000
366000
368000
370000
372000
x coordinate (EPSG:32633)

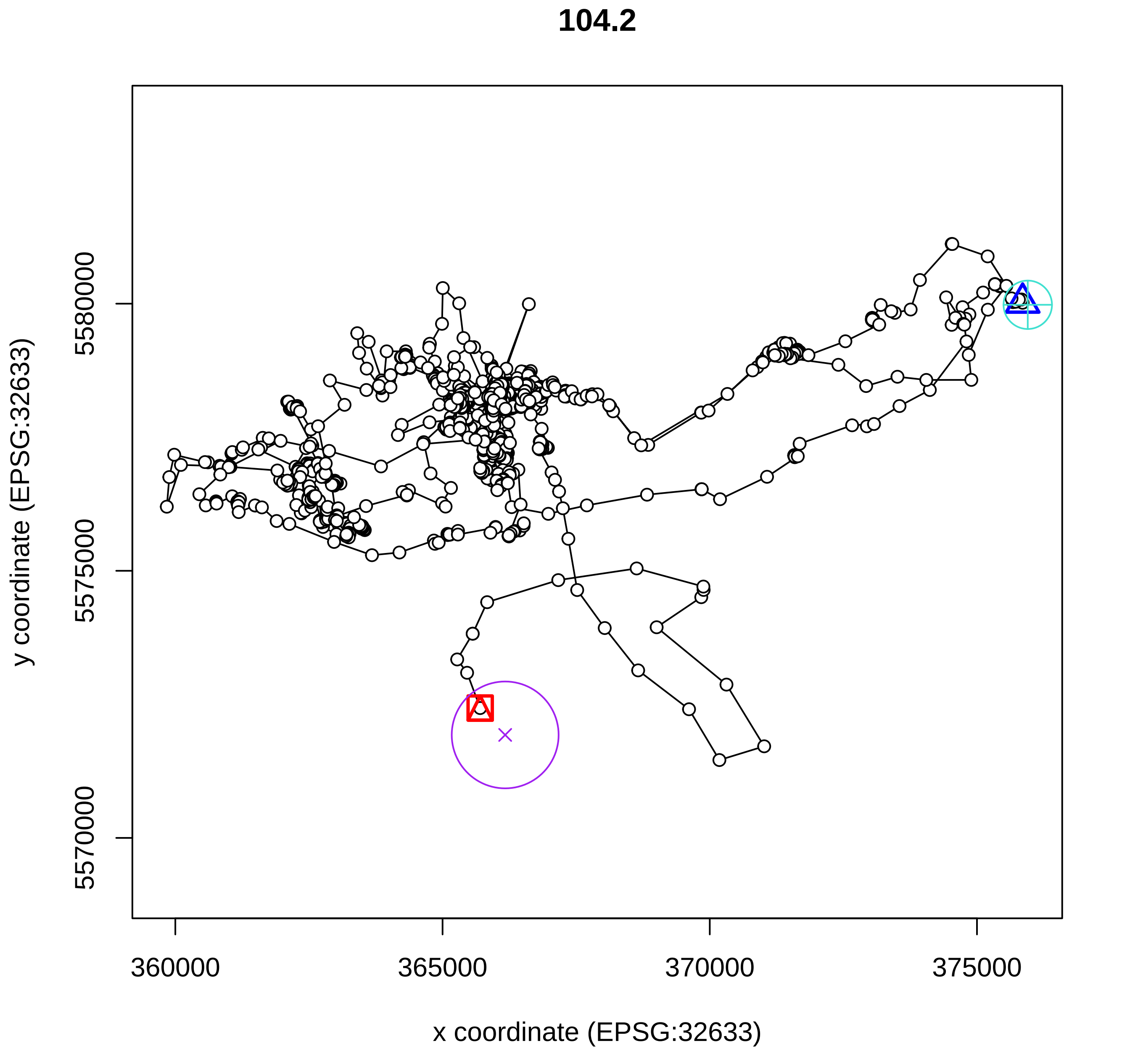
104.2
y coordinate (EPSG:32633)
5580000
5575000
5570000
x coordinate (EPSG:32633)
360000
365000
370000
375000

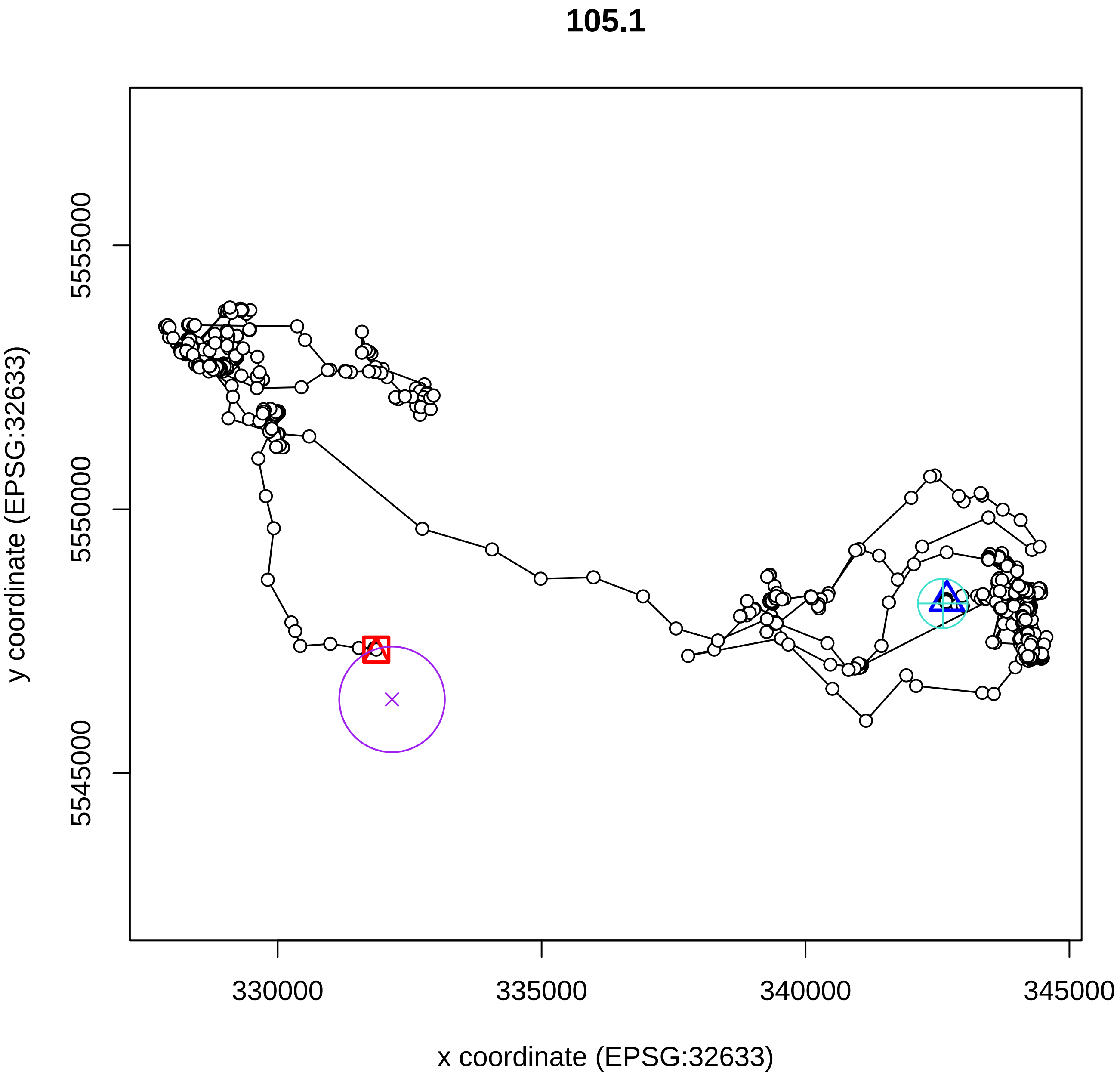

105.1
y coordinate (EPSG:32633)
5545000
5550000
5555000
330000
335000
340000
345000
x coordinate (EPSG:32633)

105.2

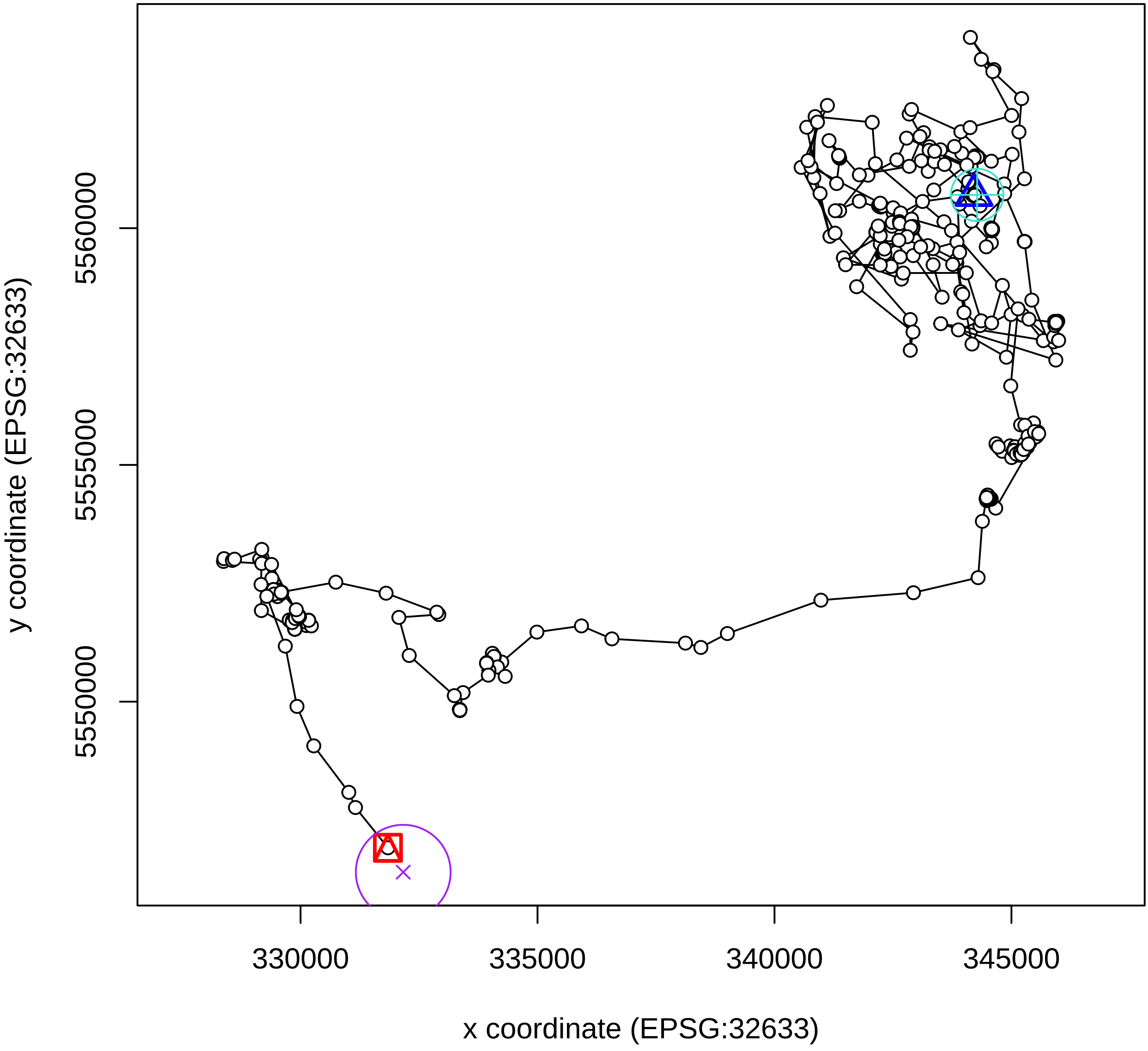

y coordinate (EPSG:32633)
5550000
5555000
5560000
330000
335000
340000
345000
x coordinate (EPSG:32633)

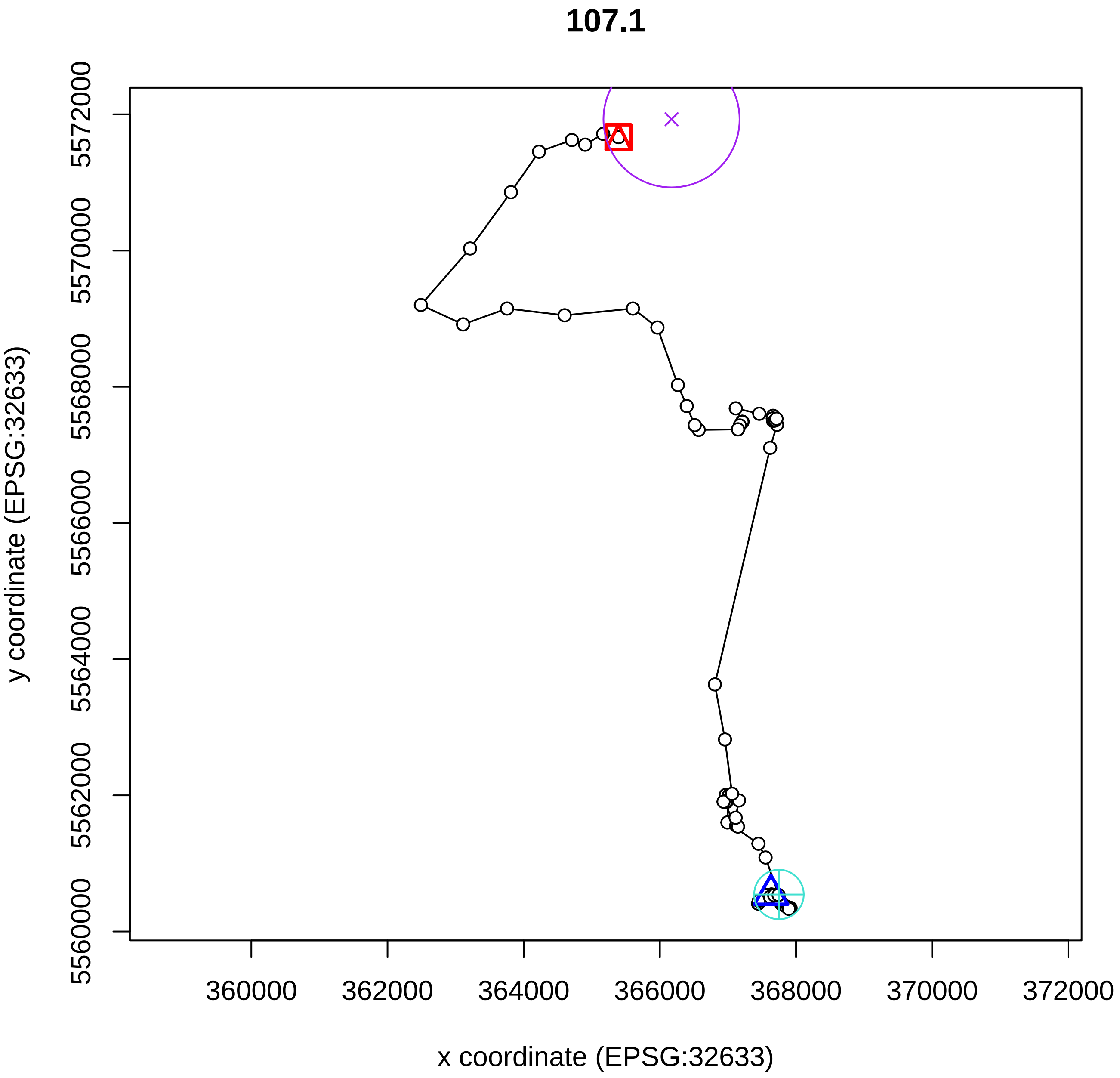
107.1
5560000
5562000
5564000
5566000
5568000
5570000
5572000
y coordinate (EPSG:32633)
360000
362000
364000
366000
368000
370000
372000
x coordinate (EPSG:32633)

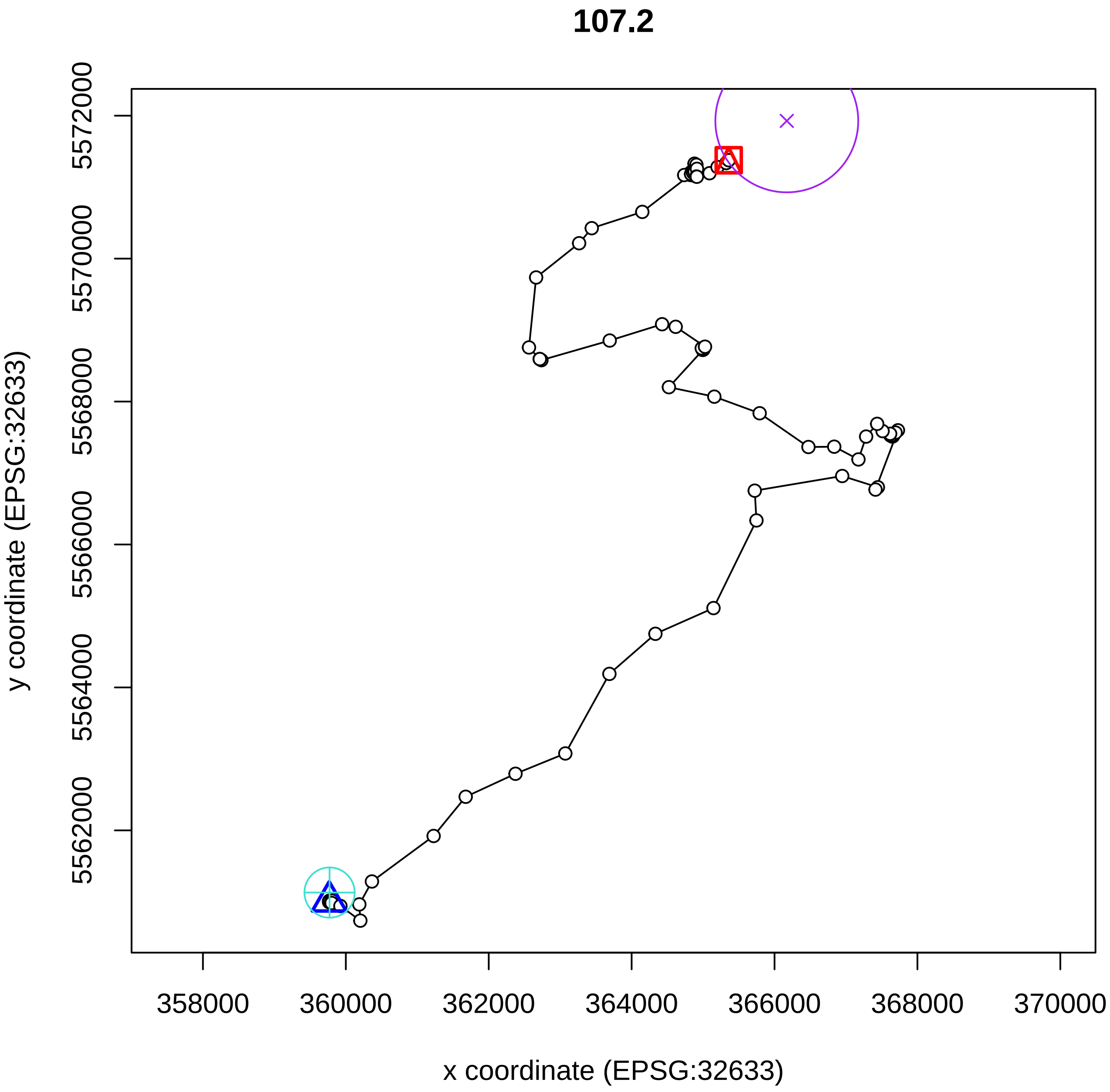
107.2
5572000
5570000
5568000
5566000
5564000
5562000
y coordinate (EPSG:32633)
358000
360000
362000
364000
366000
368000
370000
x coordinate (EPSG:32633)

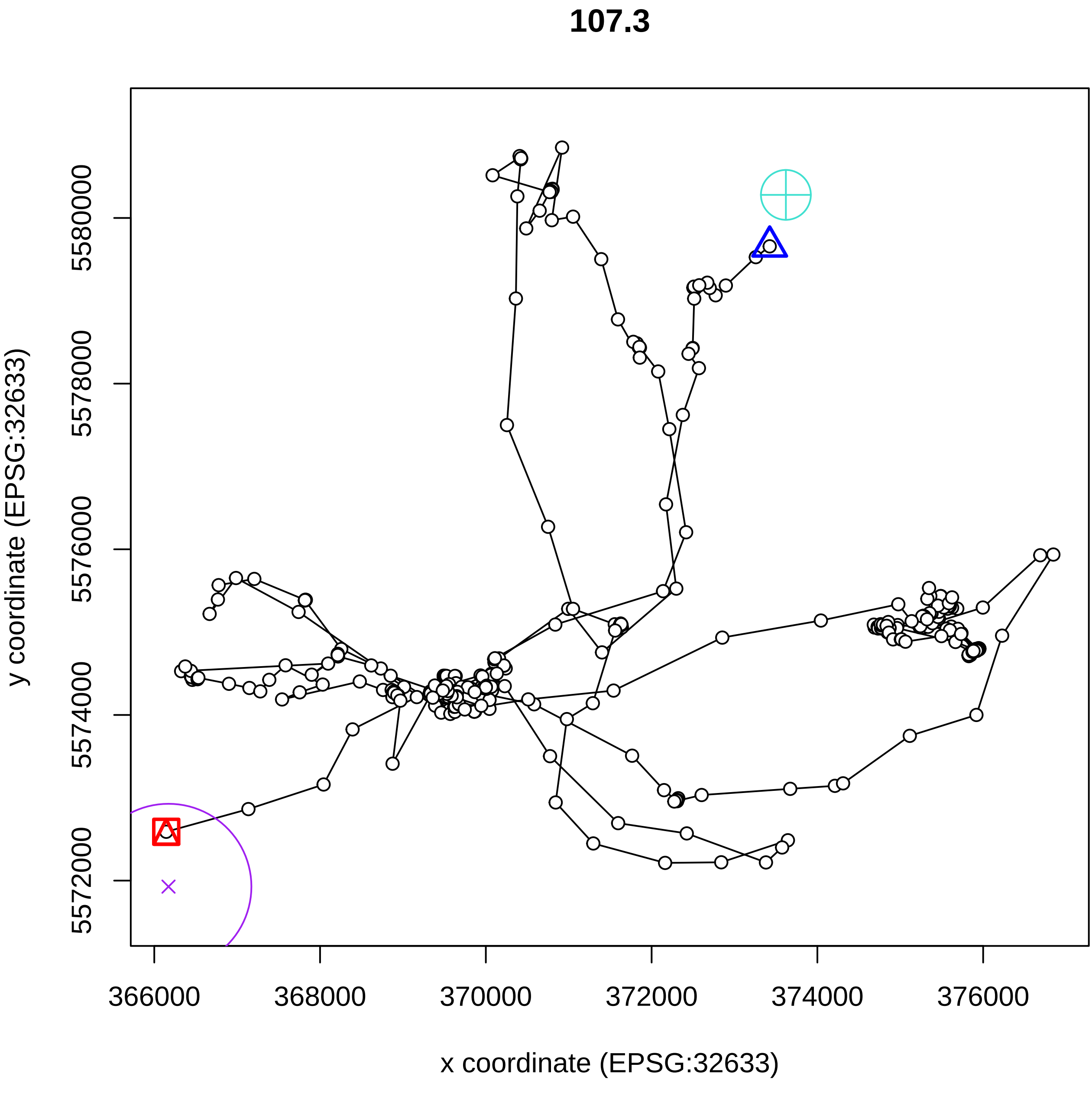
107.3
y coordinate (EPSG:32633)
5572000
5574000
5576000
5578000
5580000
366000
368000
370000
372000
374000
376000
x coordinate (EPSG:32633)

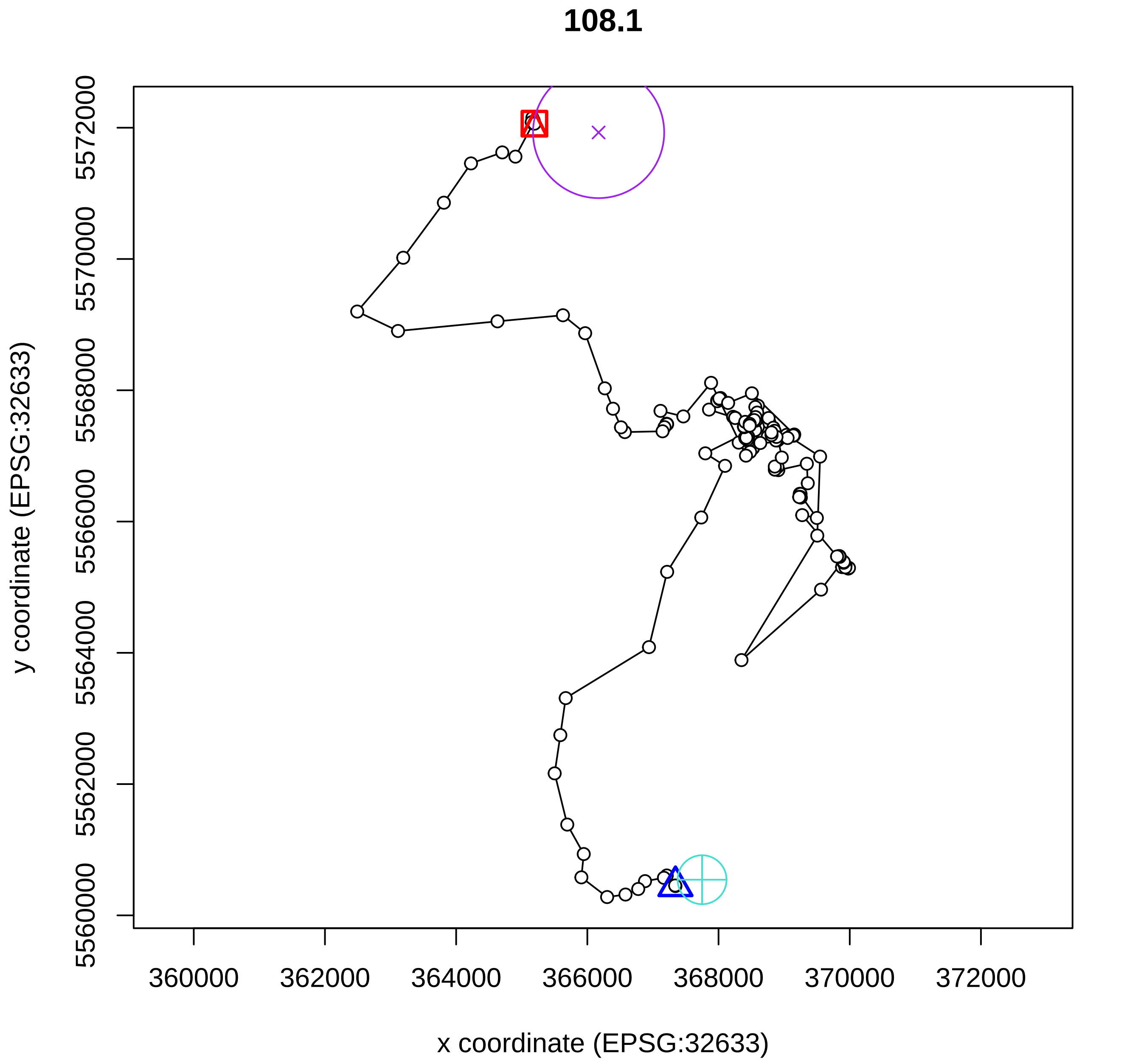
108.1
y coordinate (EPSG:32633)
5560000
5562000
5564000
5566000
5568000
5570000
5572000
360000
362000
364000
366000
368000
370000
372000
x coordinate (EPSG:32633)

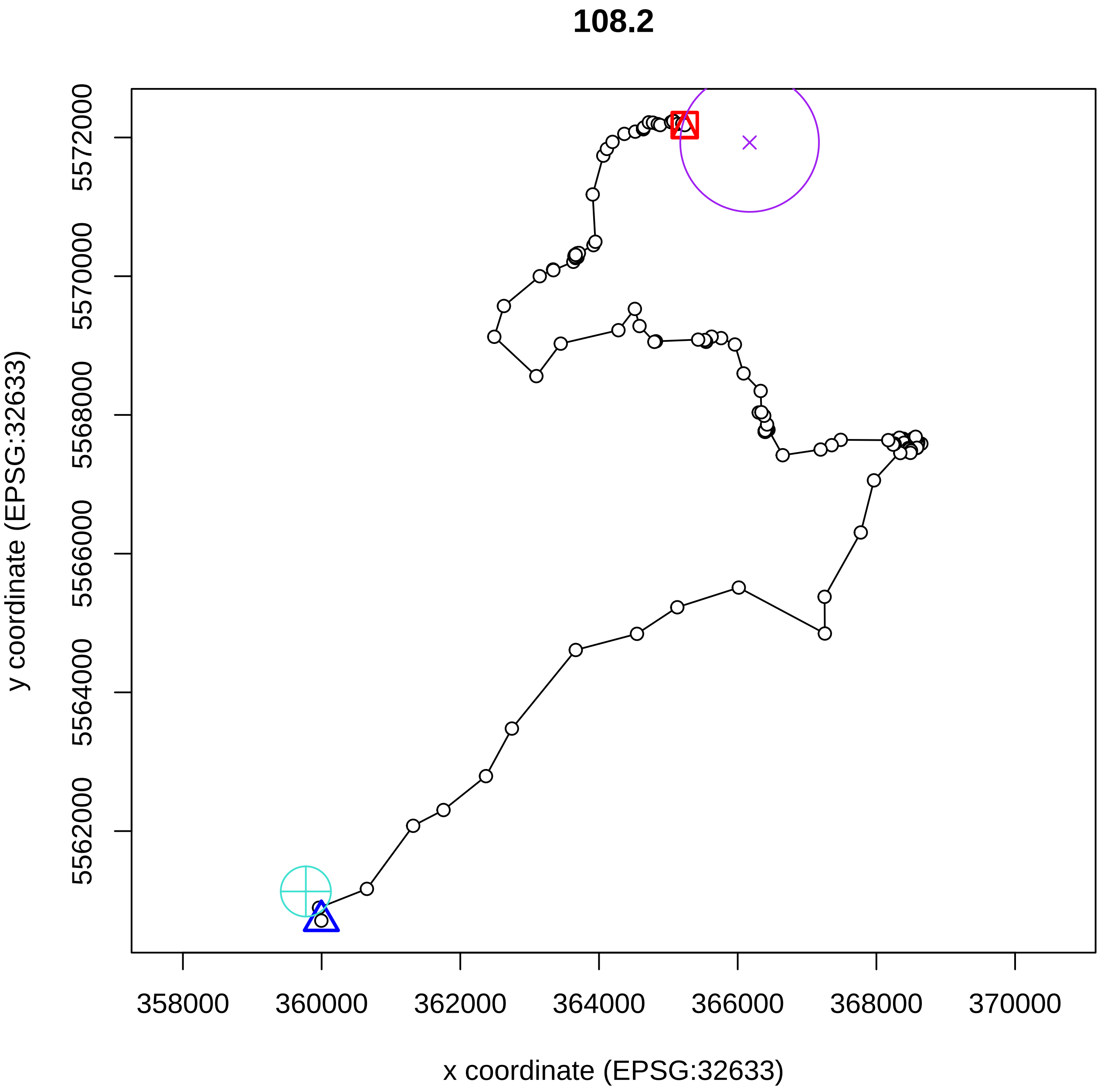
108.2
5572000
5570000
5568000
5566000
5564000
5562000
y coordinate (EPSG:32633)
358000
360000
362000
364000
366000
368000
370000
x coordinate (EPSG:32633)

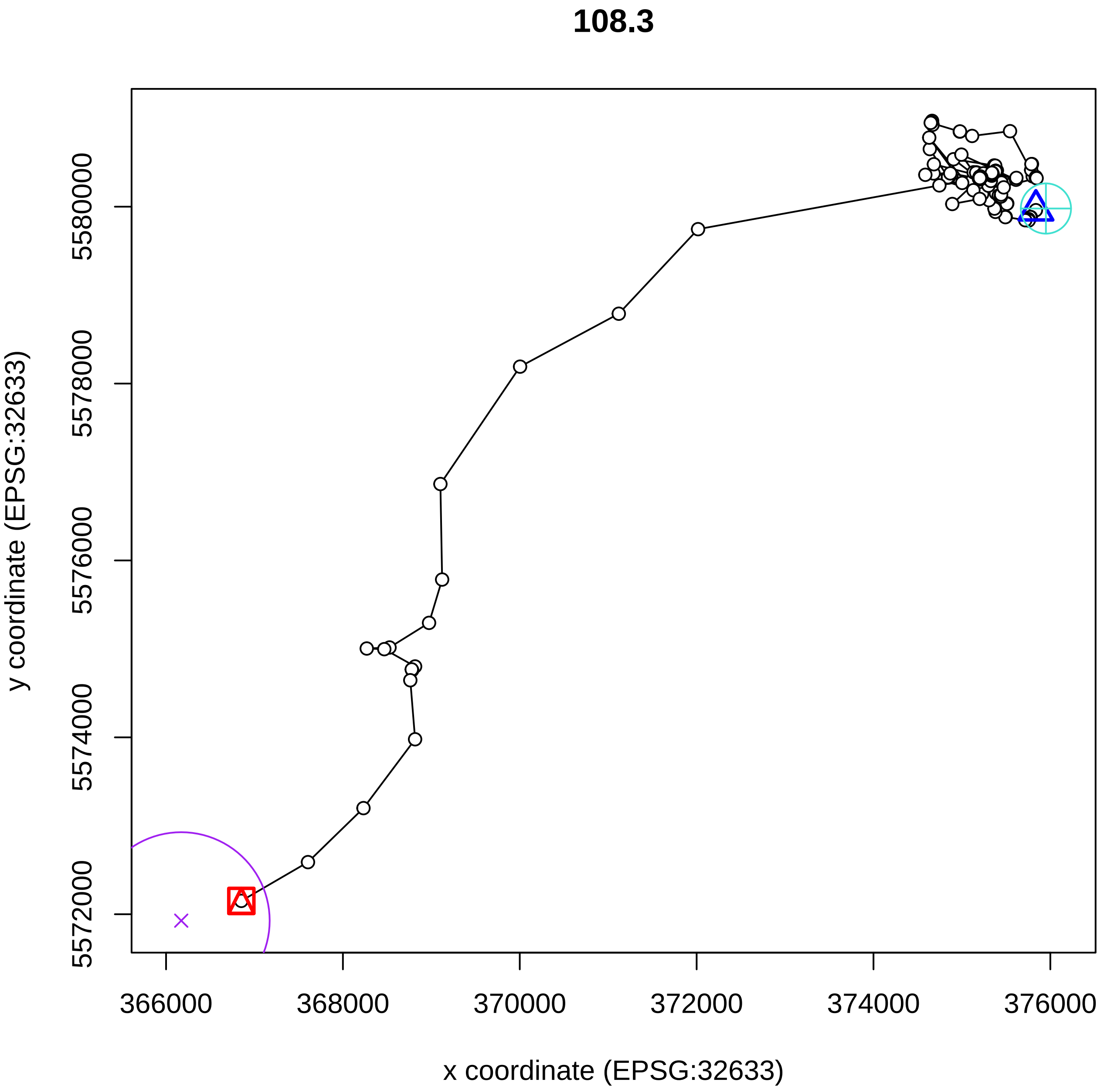

108.3
5572000
5574000
5576000
5578000
5580000
y coordinate (EPSG:32633)
366000
368000
370000
372000
374000
376000
x coordinate (EPSG:32633)

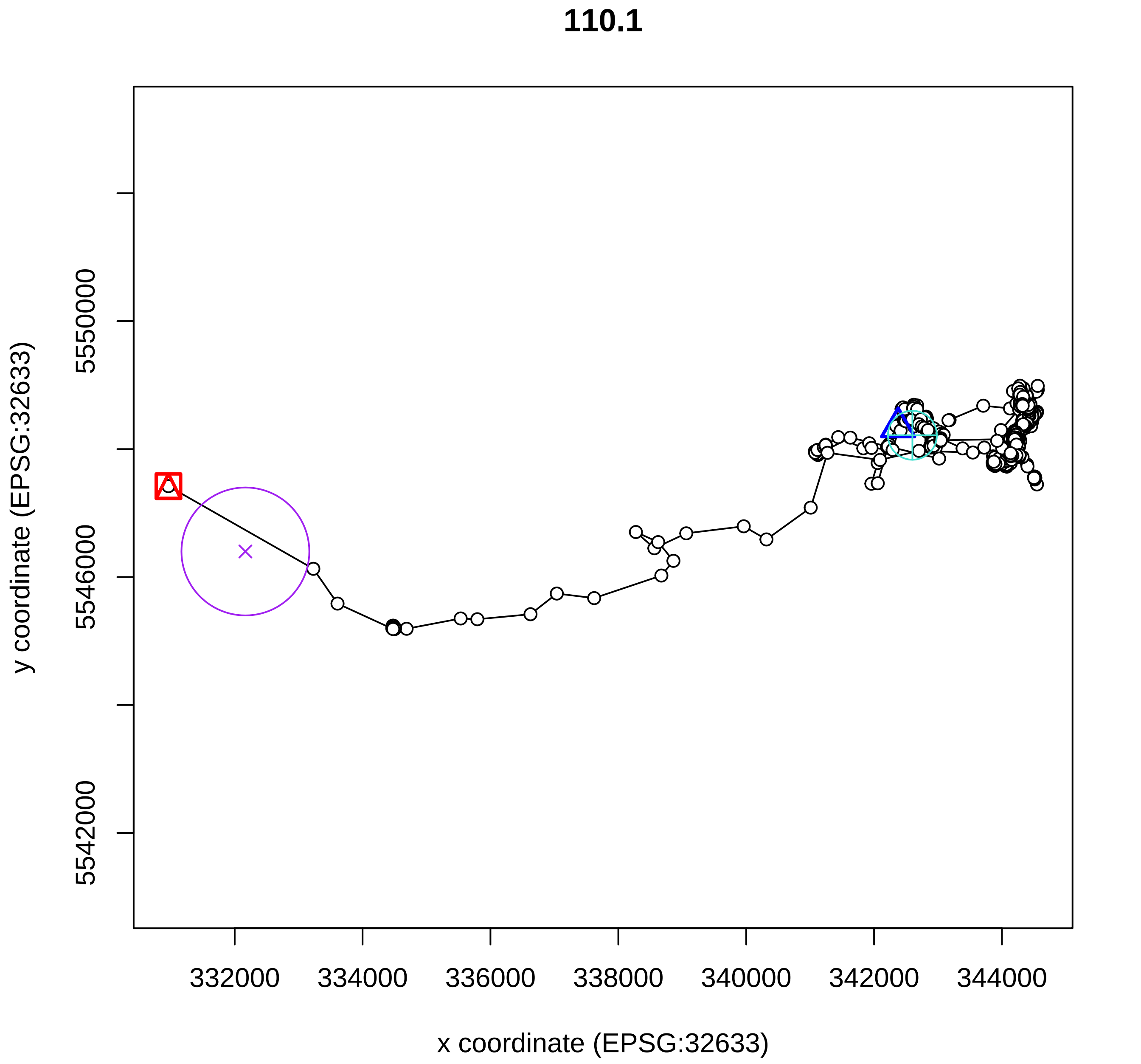
110.1
y coordinate (EPSG:32633)
5542000
5546000
5550000
x coordinate (EPSG:32633)
332000
334000
336000
338000
340000
342000
344000

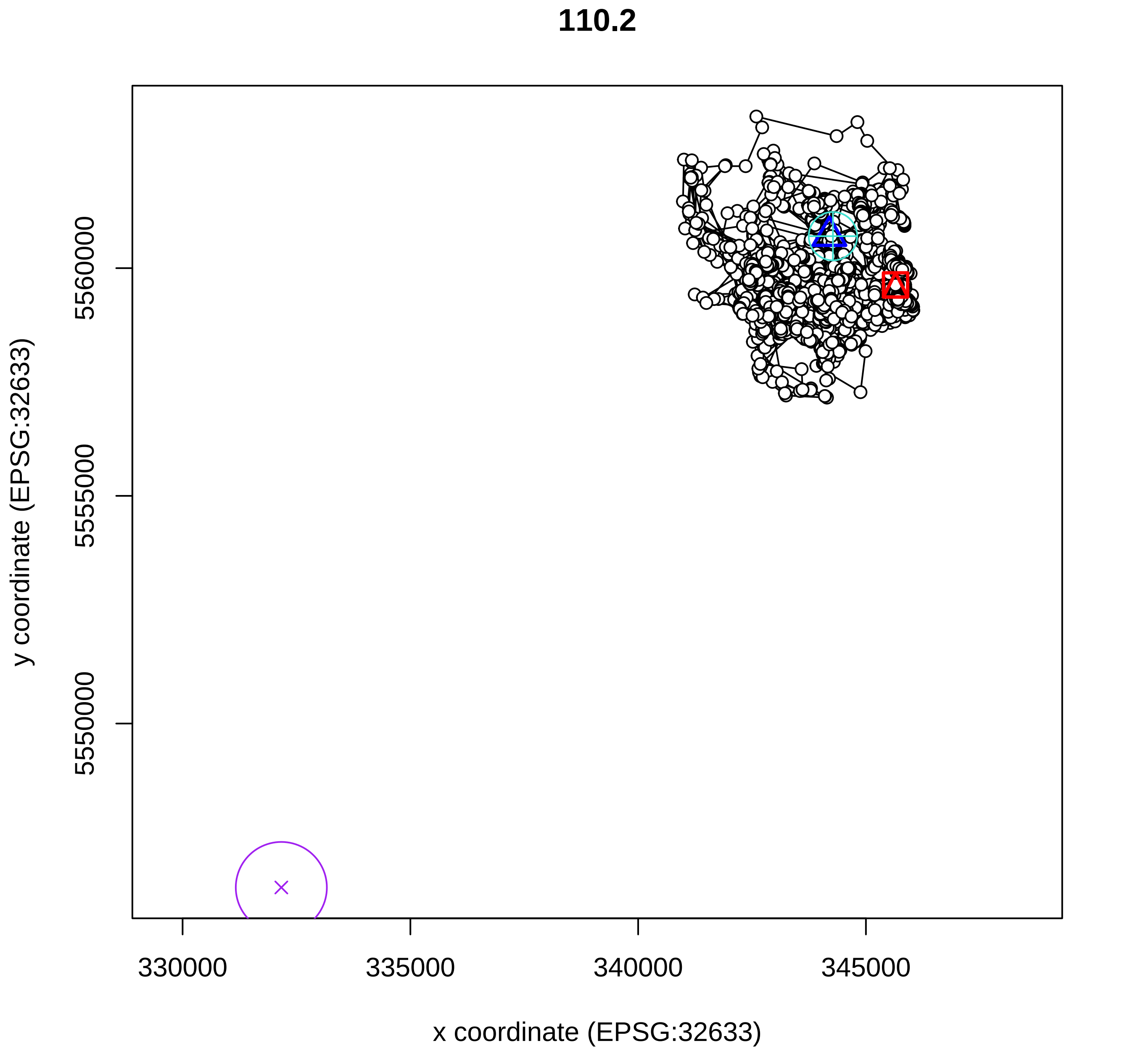
110.2
y coordinate (EPSG:32633)
5550000
5555000
5560000
330000
335000
340000
345000
x coordinate (EPSG:32633)

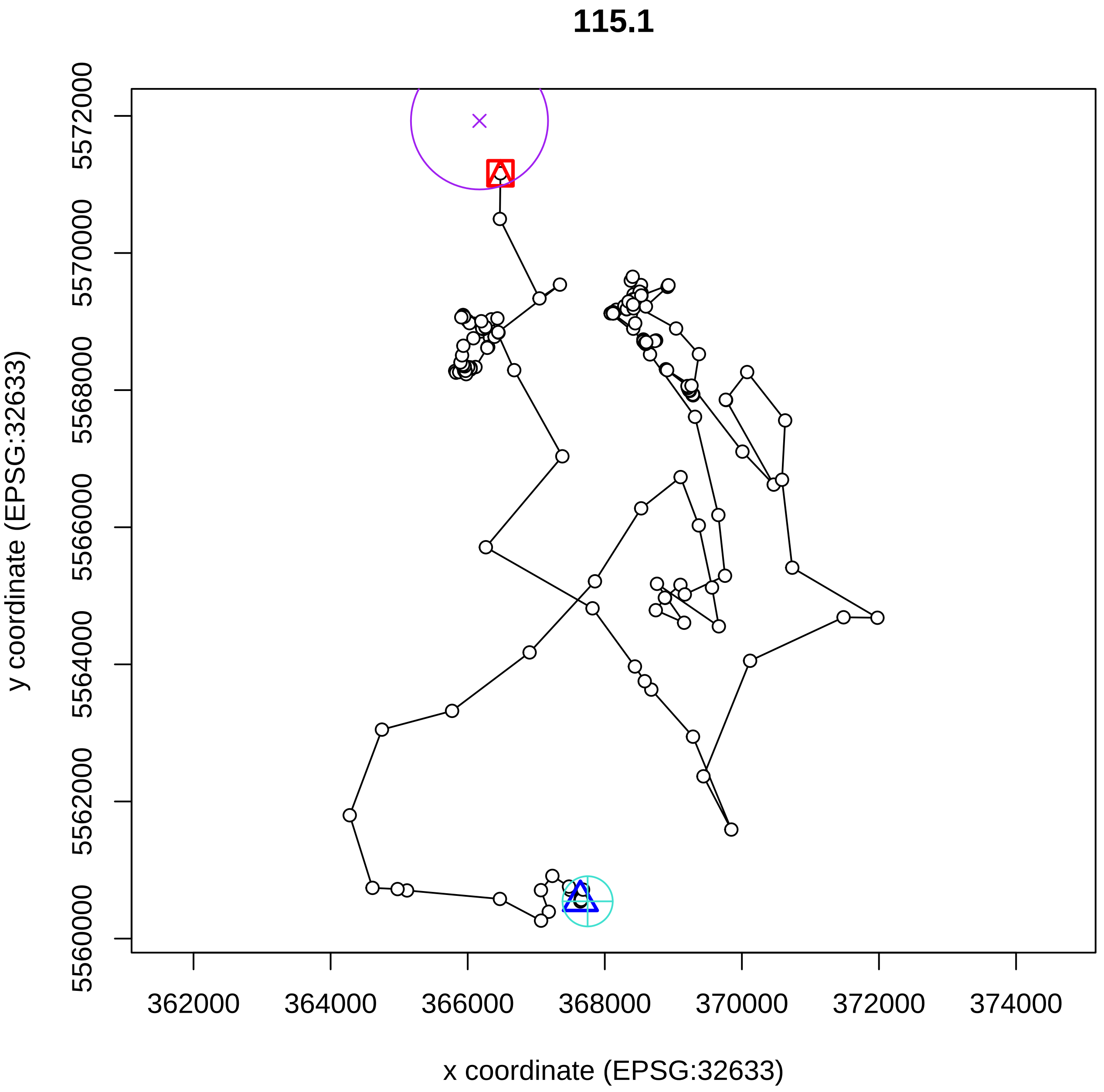
115.1
y coordinate (EPSG:32633)
5560000
5562000
5564000
5566000
5568000
5570000
5572000
362000
364000
366000
368000
370000
372000
374000
x coordinate (EPSG:32633)

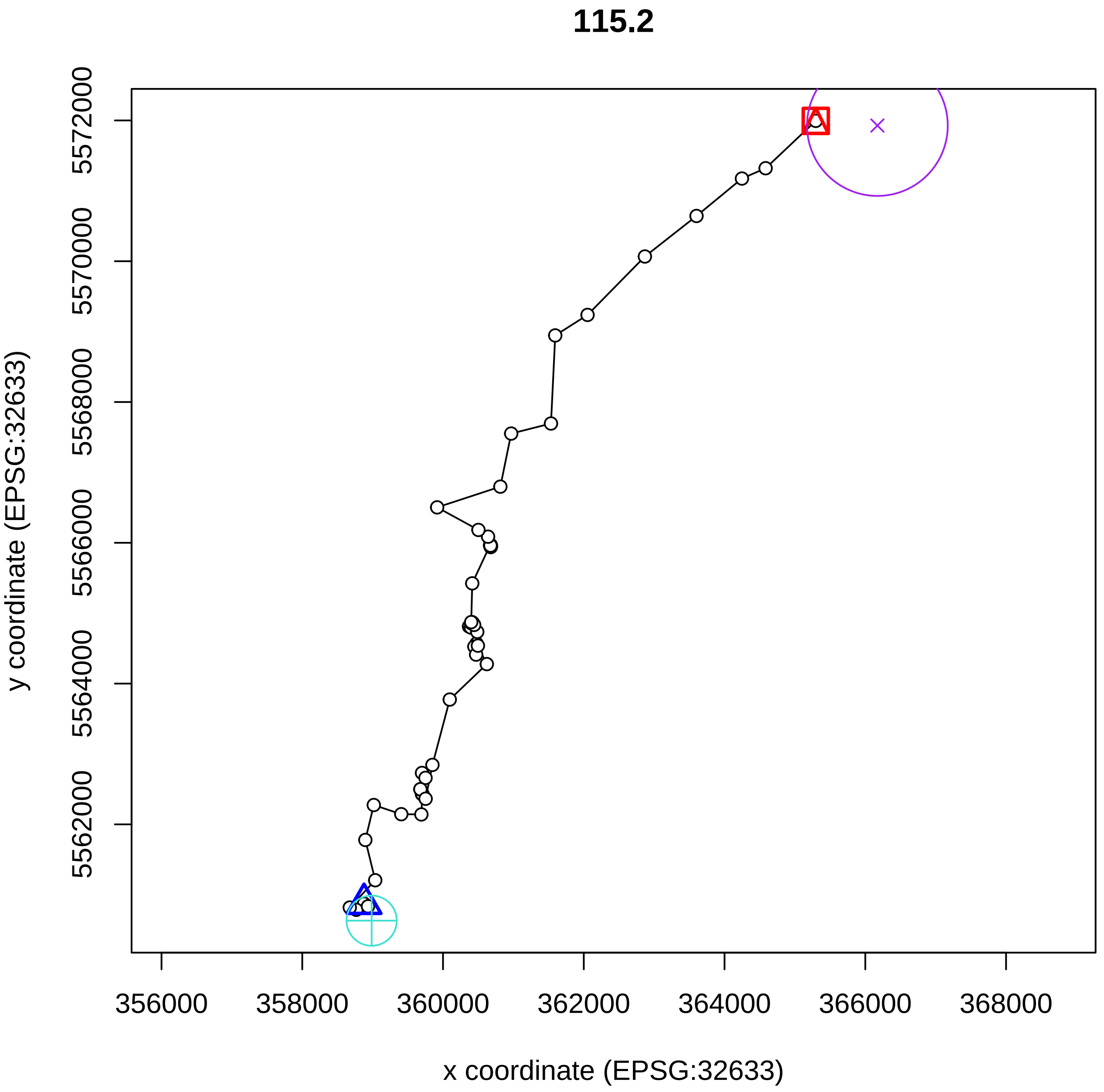

115.2
5562000
5564000
5566000
5568000
5570000
5572000
y coordinate (EPSG:32633)
356000
358000
360000
362000
364000
366000
368000
x coordinate (EPSG:32633)

117.1

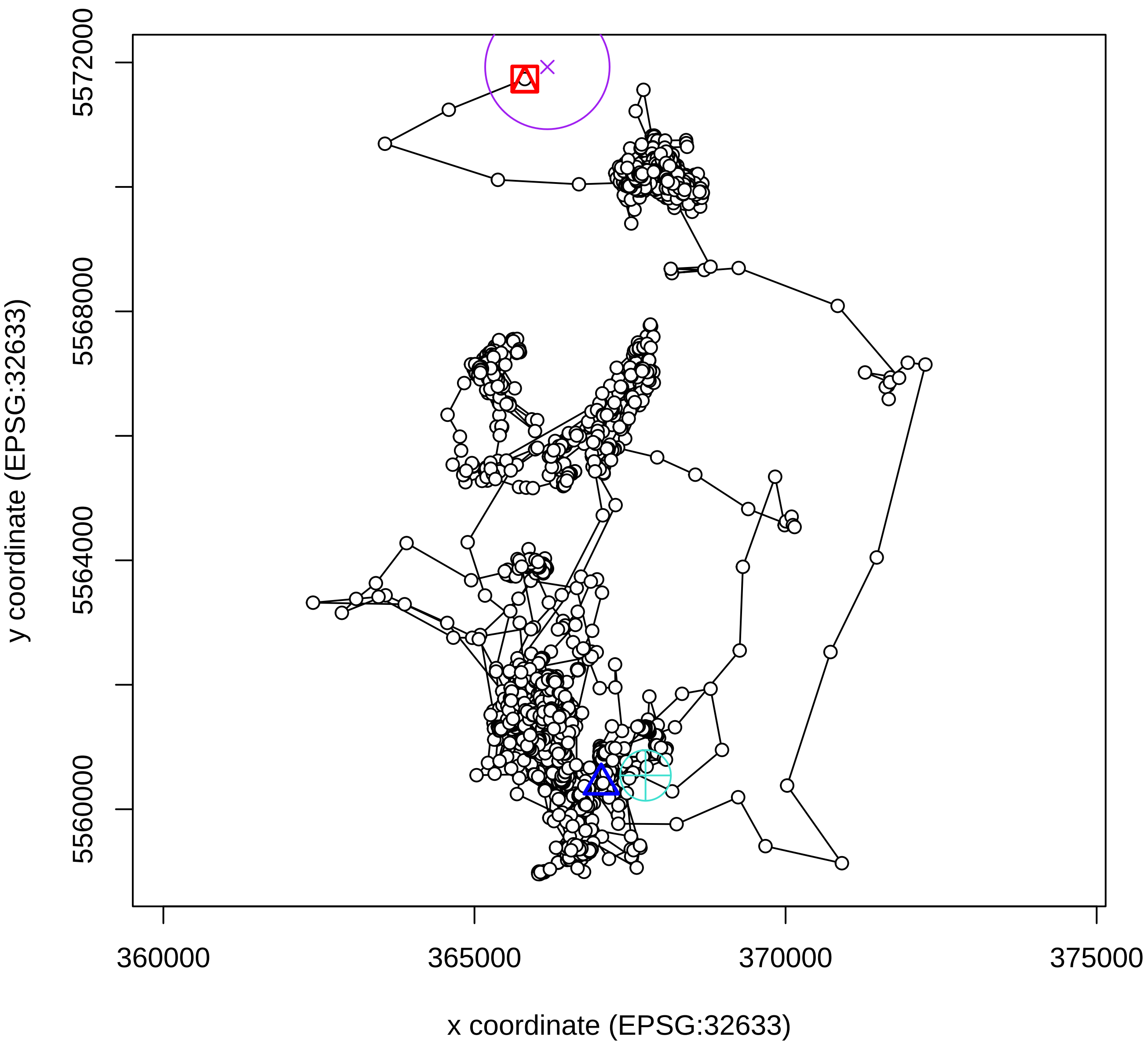

5572000
5568000
5564000
5560000
y coordinate (EPSG:32633)
360000
365000
370000
375000
x coordinate (EPSG:32633)

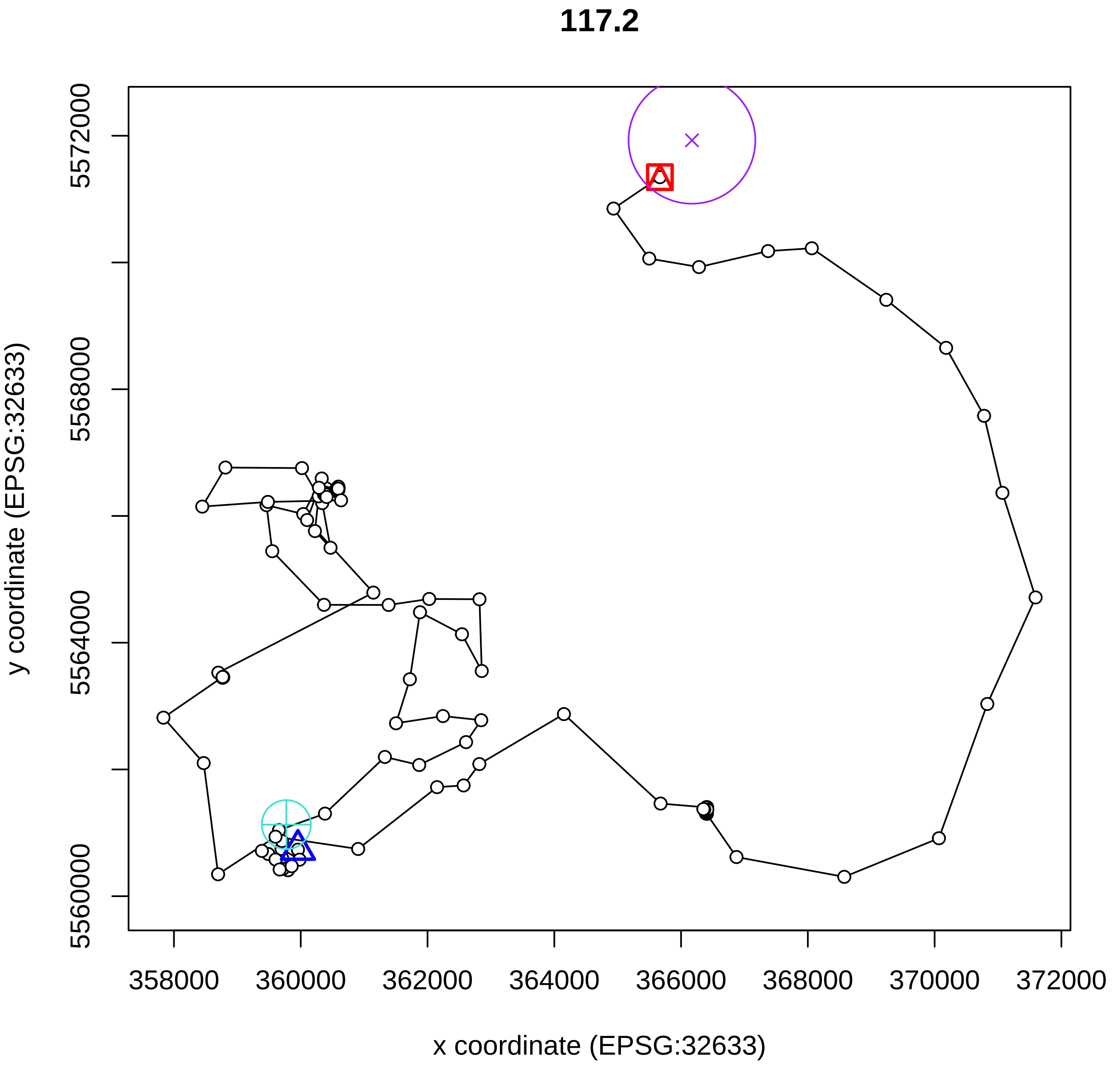
117.2
5572000
5568000
5564000
5560000
y coordinate (EPSG:32633)
358000
360000
362000
364000
366000
368000
370000
372000
x coordinate (EPSG:32633)

118.1

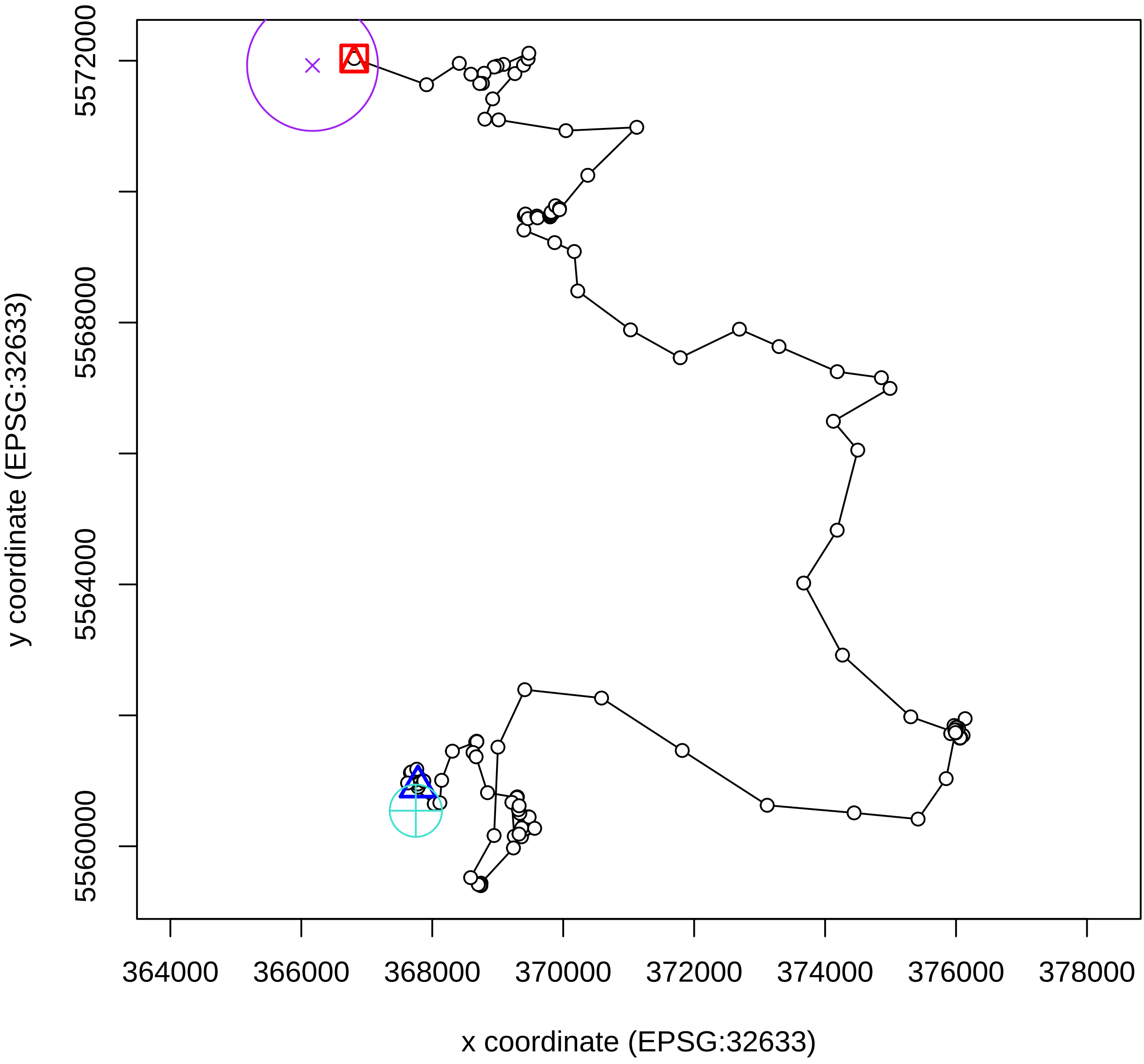

5572000
5568000
5564000
5560000
y coordinate (EPSG:32633)
364000
366000
368000
370000
372000
374000
376000
378000
x coordinate (EPSG:32633)

120.1

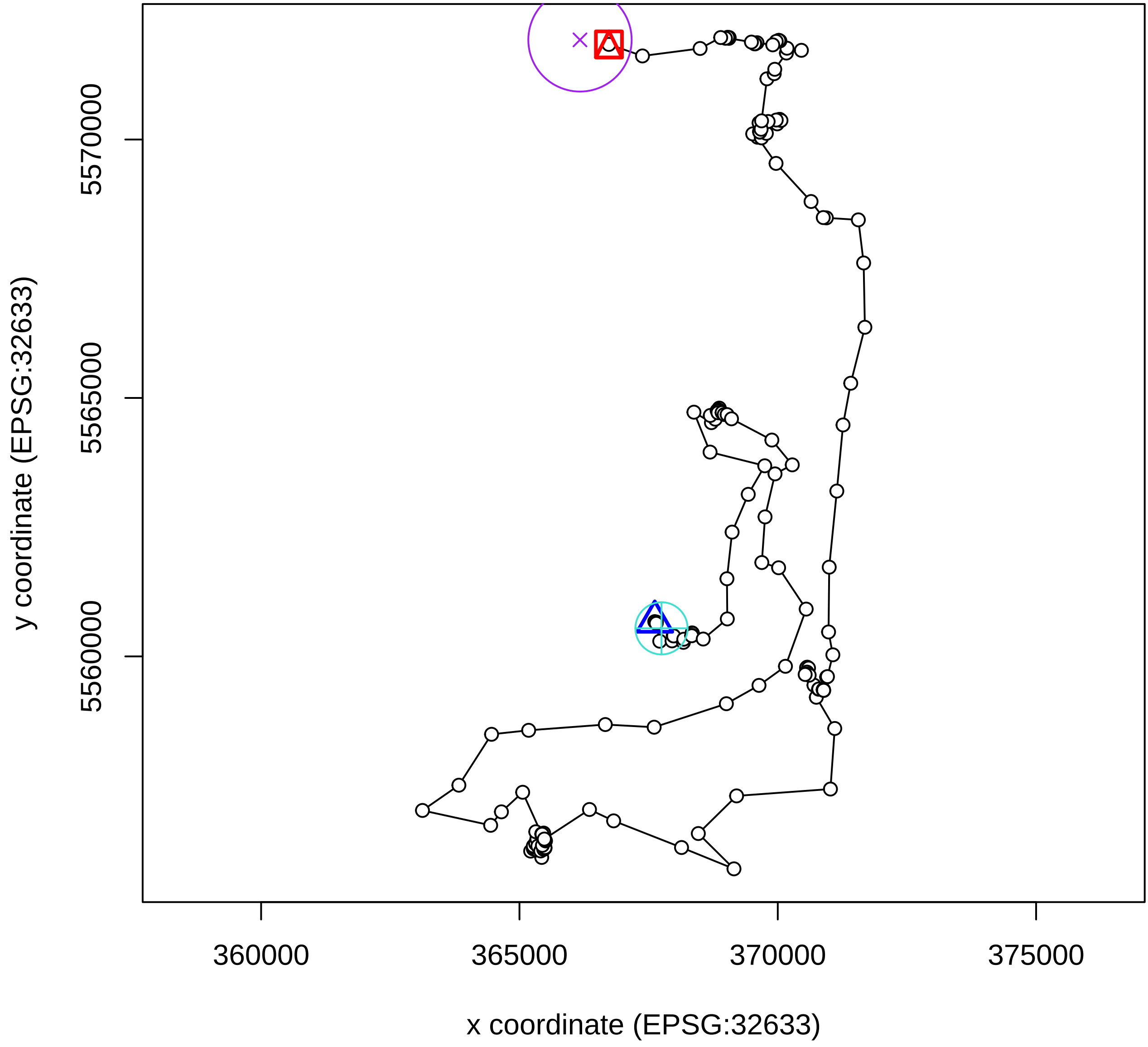

5570000
5565000
5560000
y coordinate (EPSG:32633)
360000
365000
370000
375000
x coordinate (EPSG:32633)

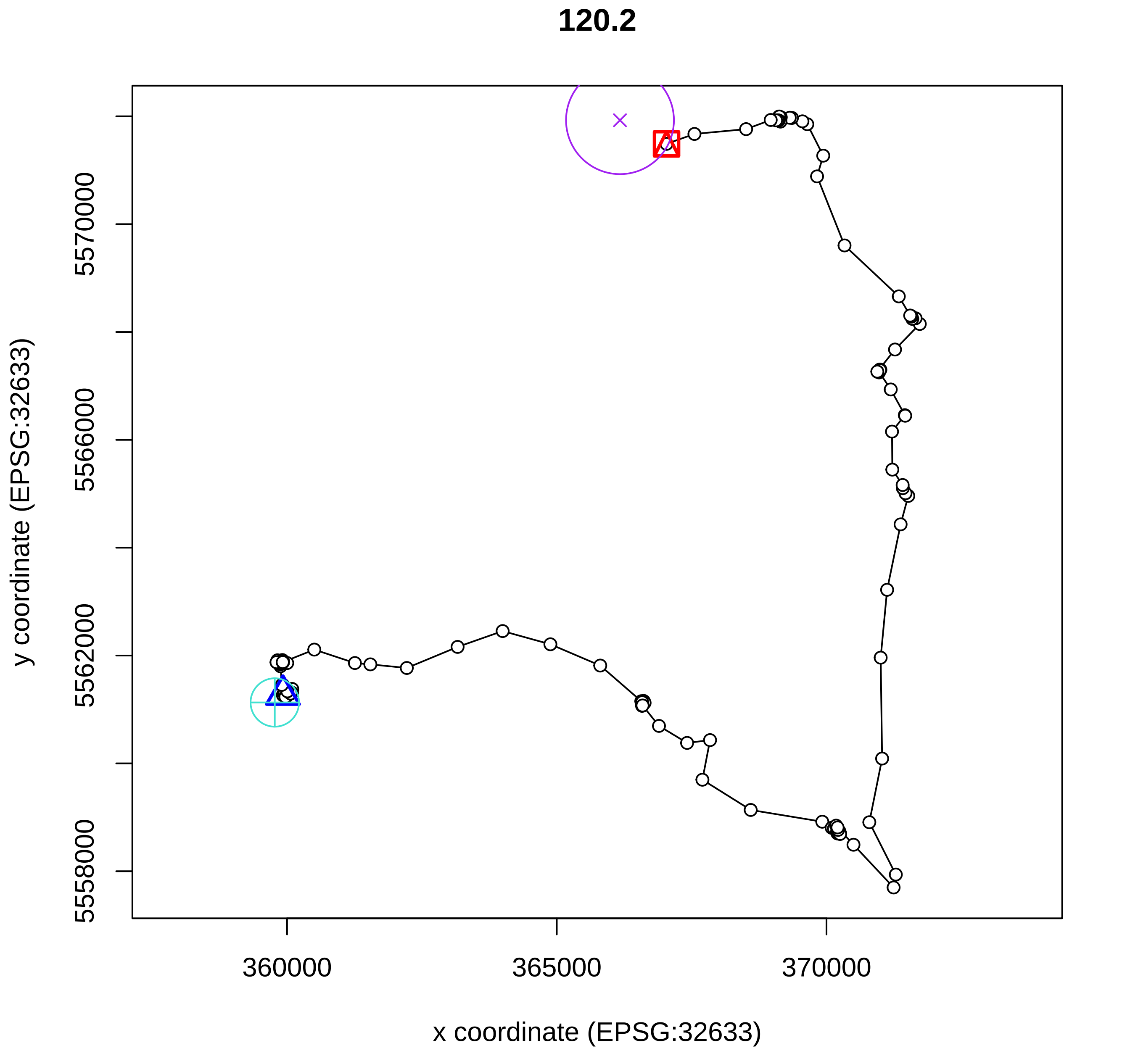

120.2
y coordinate (EPSG:32633)
5558000
5562000
5566000
5570000
360000
365000
370000
x coordinate (EPSG:32633)

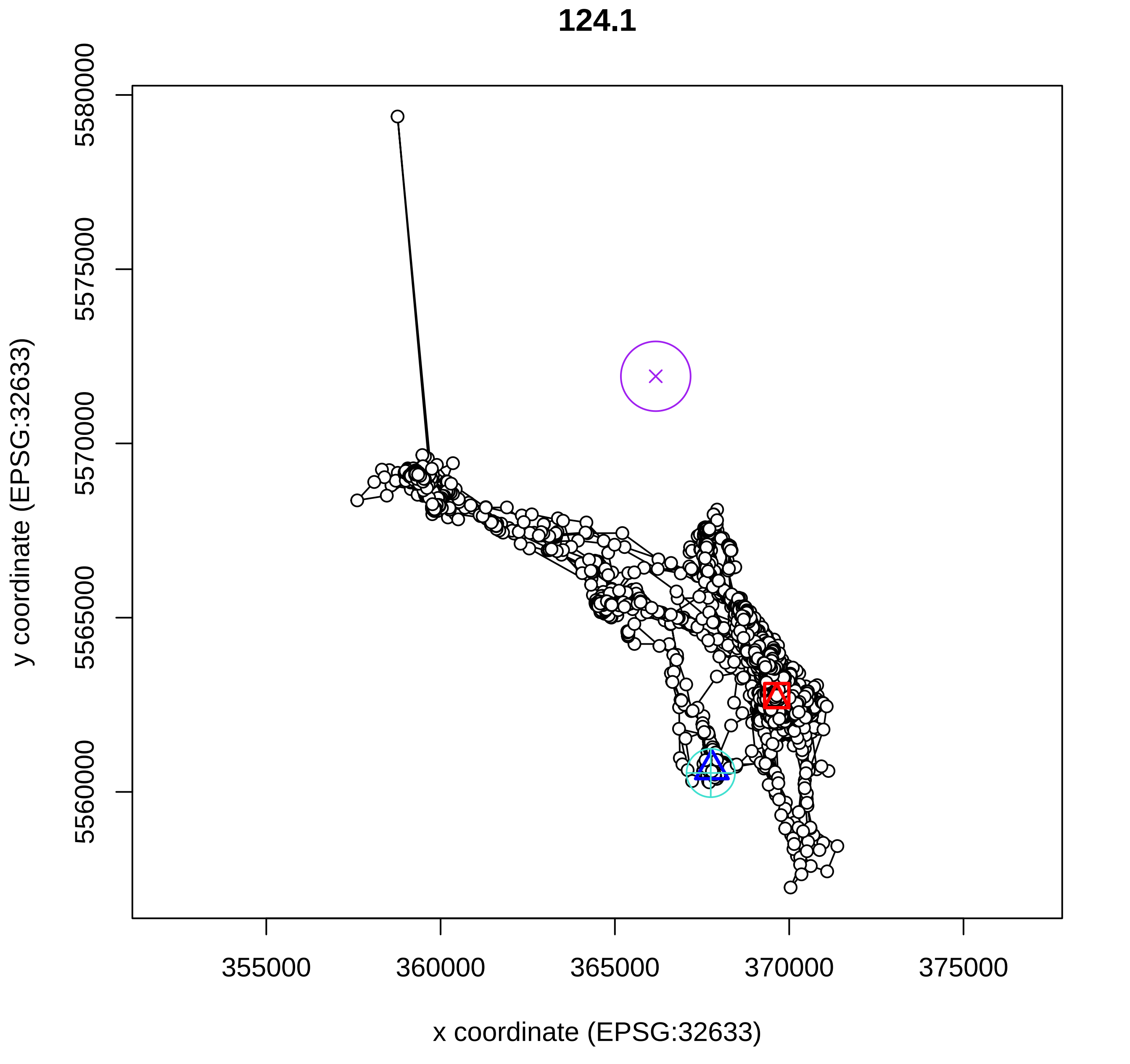
124.1
y coordinate (EPSG:32633)
5580000
5575000
5570000
5565000
5560000
x coordinate (EPSG:32633)
355000
360000
365000
370000
375000

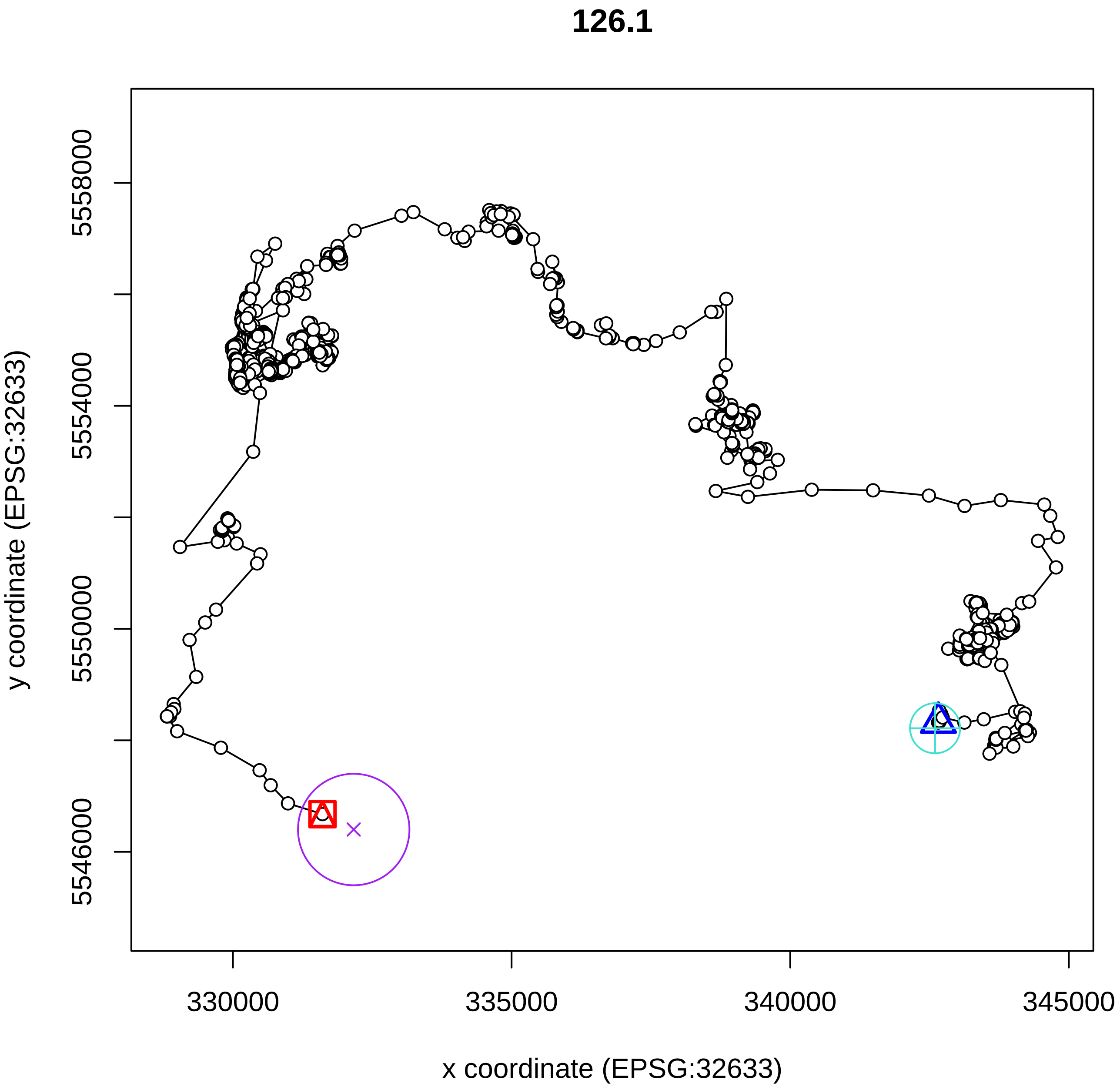
126.1
5558000
5554000
5550000
5546000
y coordinate (EPSG:32633)
330000
335000
340000
345000
x coordinate (EPSG:32633)

132.1

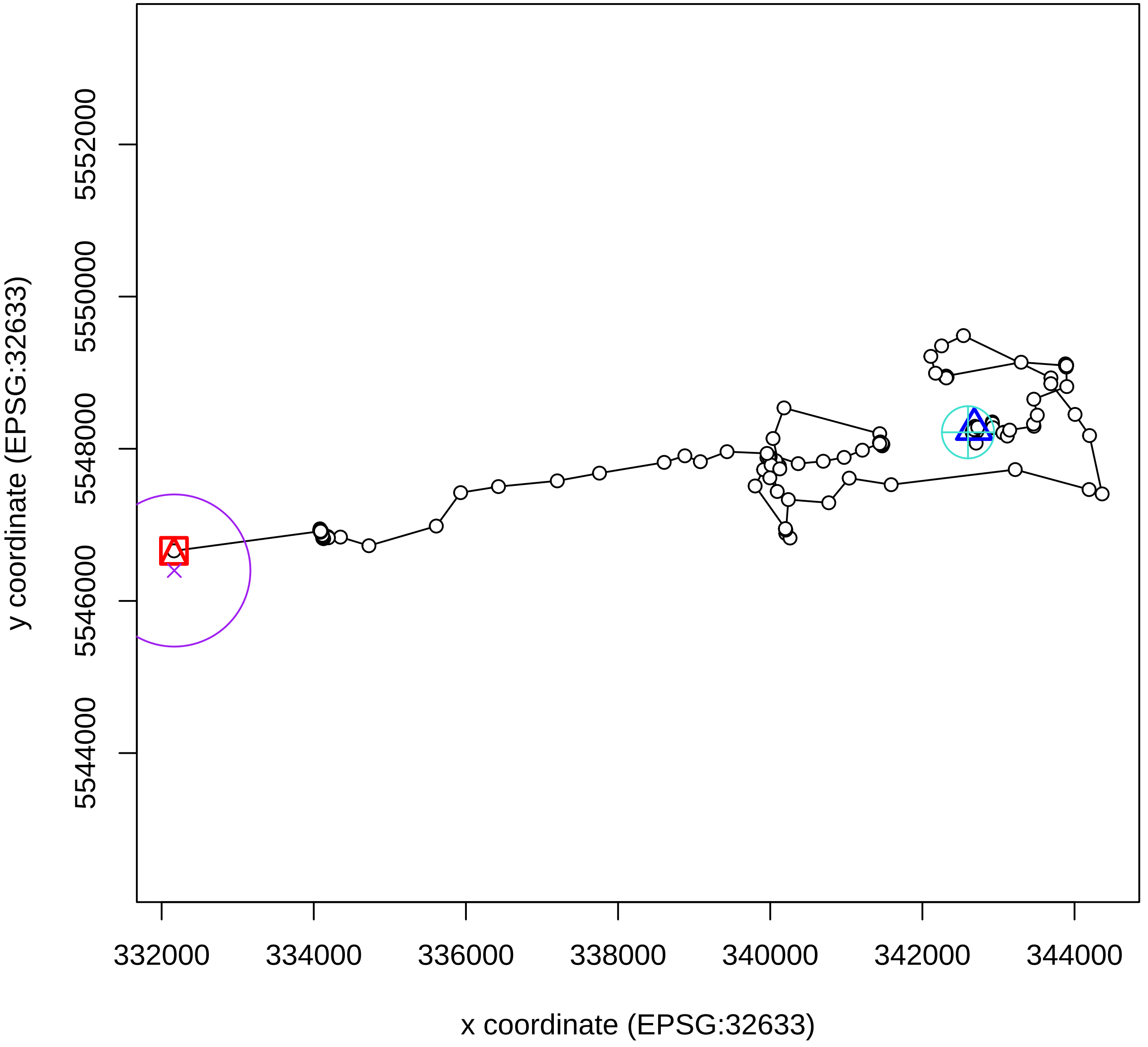

y coordinate (EPSG:32633)
5544000
5546000
5548000
5550000
5552000
332000
334000
336000
338000
340000
342000
344000
x coordinate (EPSG:32633)

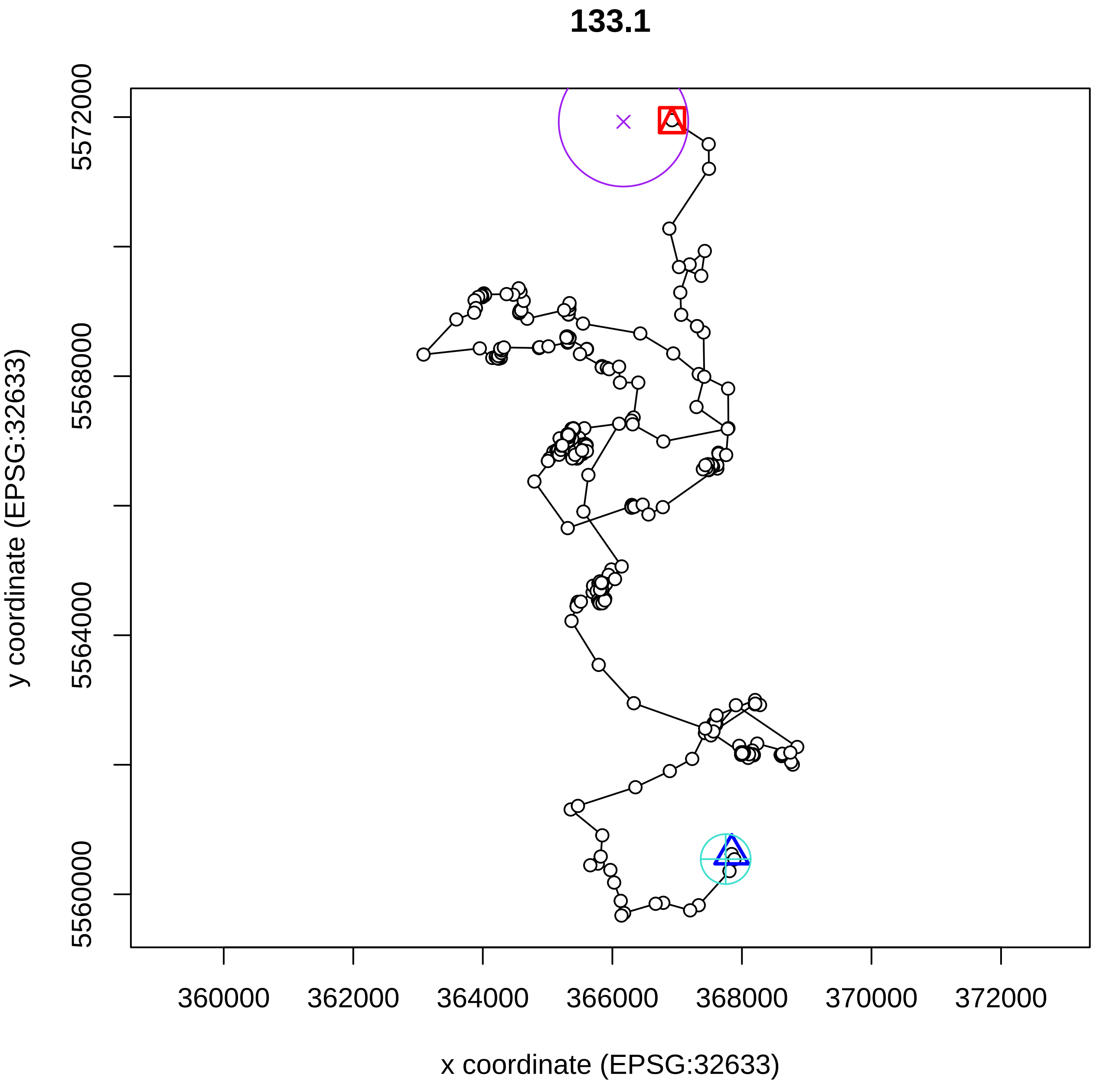

133.1
5572000
5568000
5564000
5560000
y coordinate (EPSG:32633)
360000
362000
364000
366000
368000
370000
372000
x coordinate (EPSG:32633)

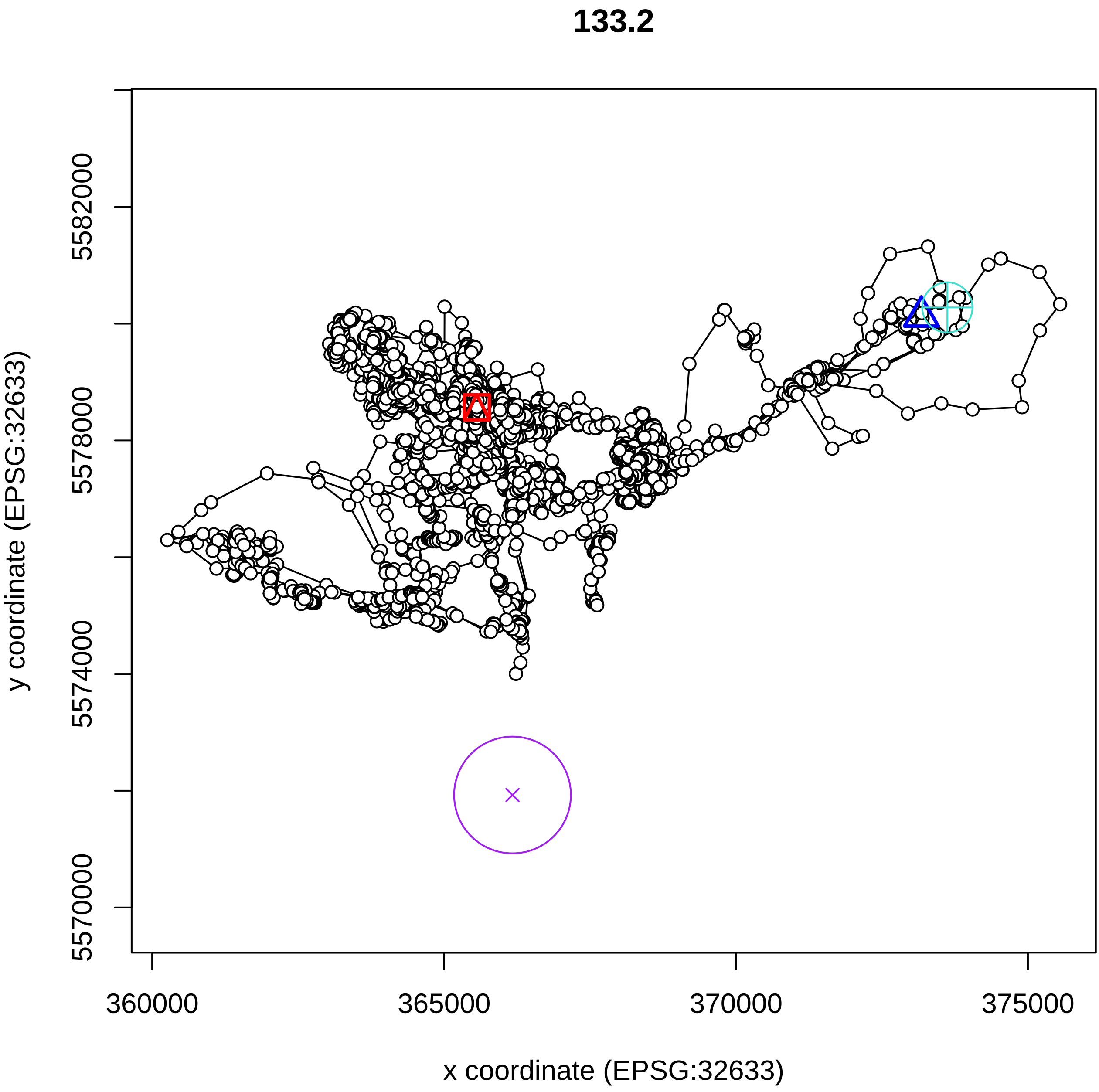
133.2
y coordinate (EPSG:32633)
5570000
5574000
5578000
5582000
x coordinate (EPSG:32633)
360000
365000
370000
375000

135.1

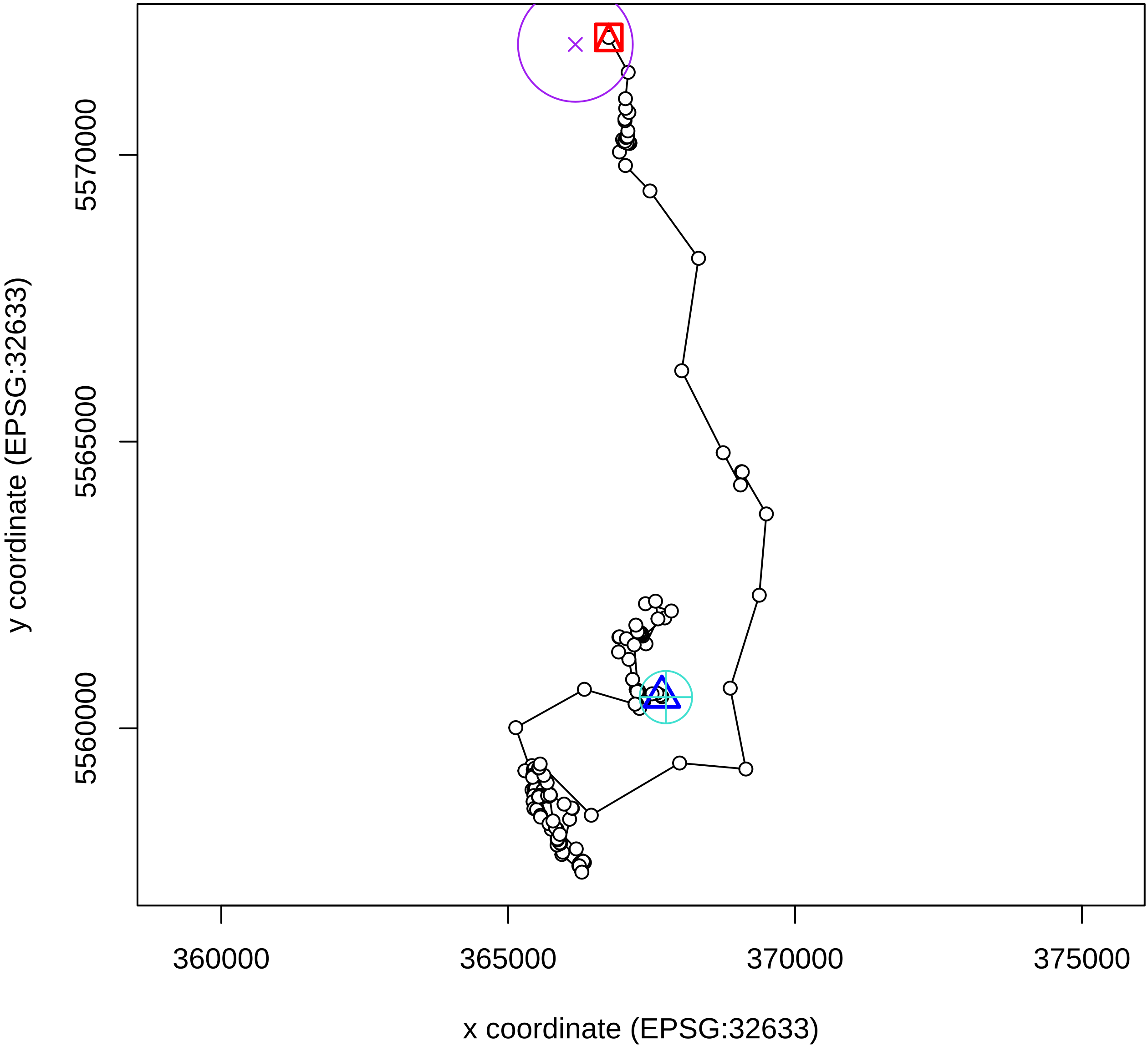

5570000
5565000
5560000
y coordinate (EPSG:32633)
360000
365000
370000
375000
x coordinate (EPSG:32633)

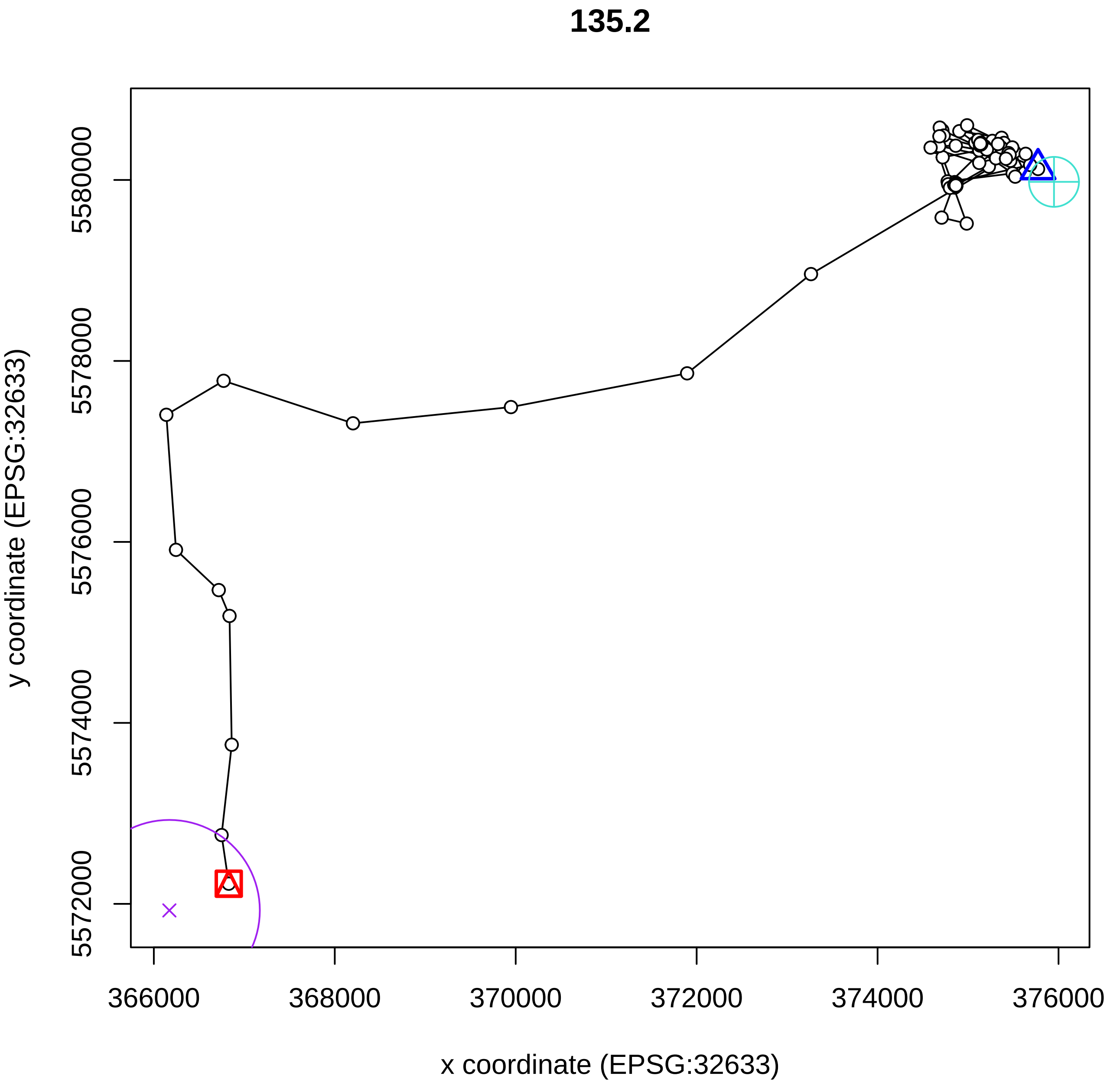

135.2
5572000
5574000
5576000
5578000
5580000
y coordinate (EPSG:32633)
366000
368000
370000
372000
374000
376000
x coordinate (EPSG:32633)

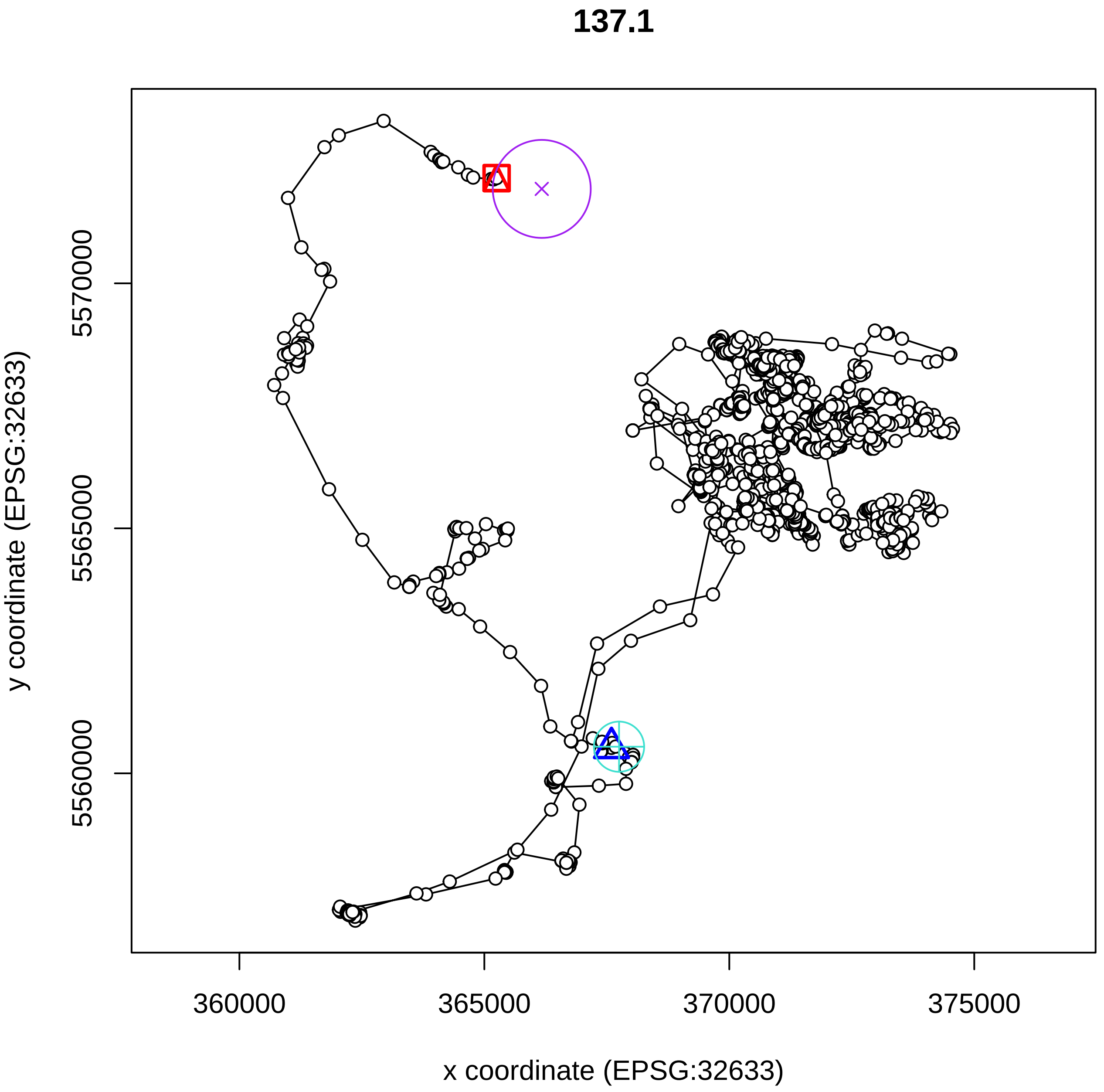
137.1
y coordinate (EPSG:32633)
5560000
5565000
5570000
x coordinate (EPSG:32633)
360000
365000
370000
375000

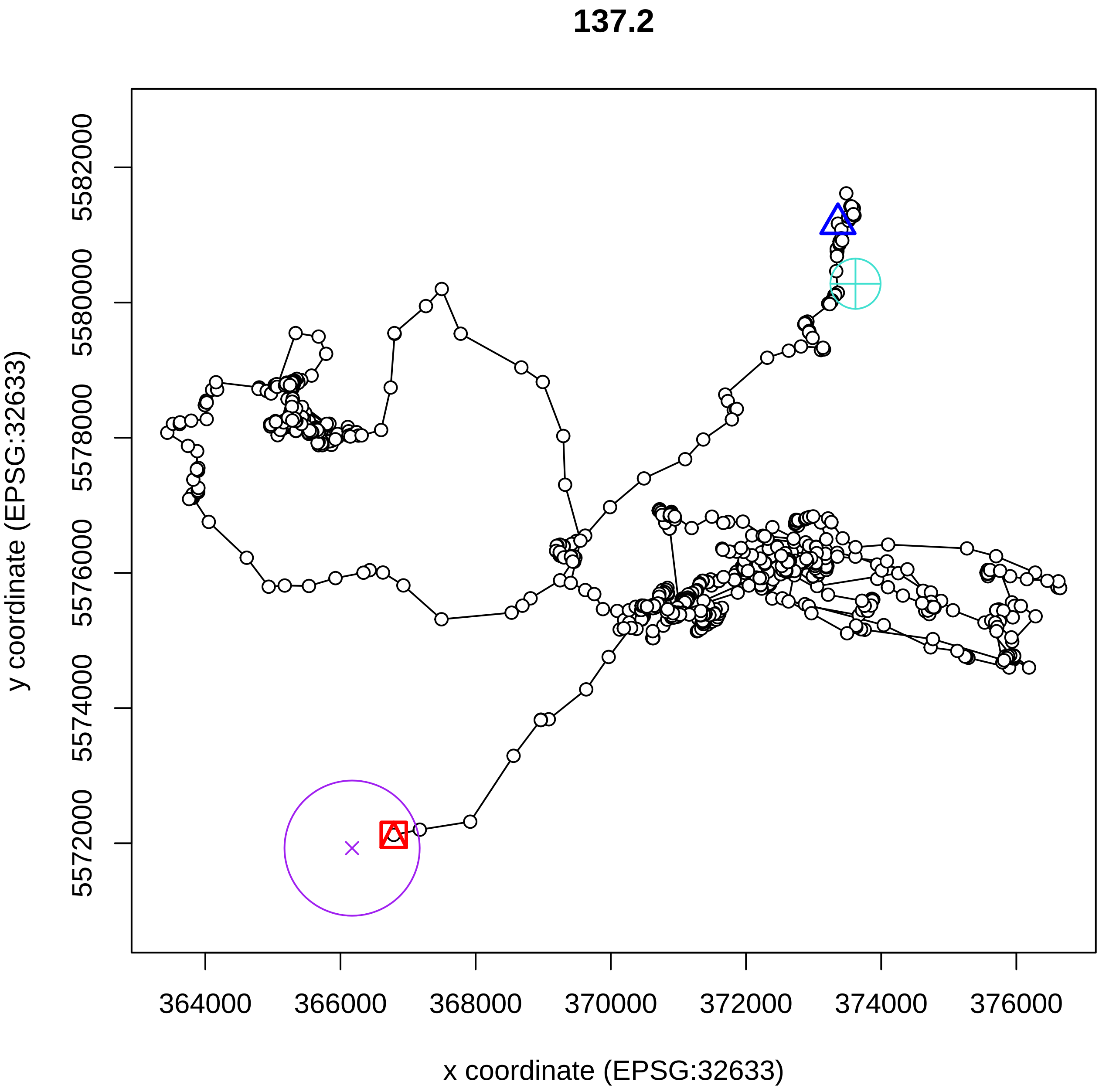
137.2
5582000
5580000
5578000
5576000
5574000
5572000
y coordinate (EPSG:32633)
364000
366000
368000
370000
372000
374000
376000
x coordinate (EPSG:32633)

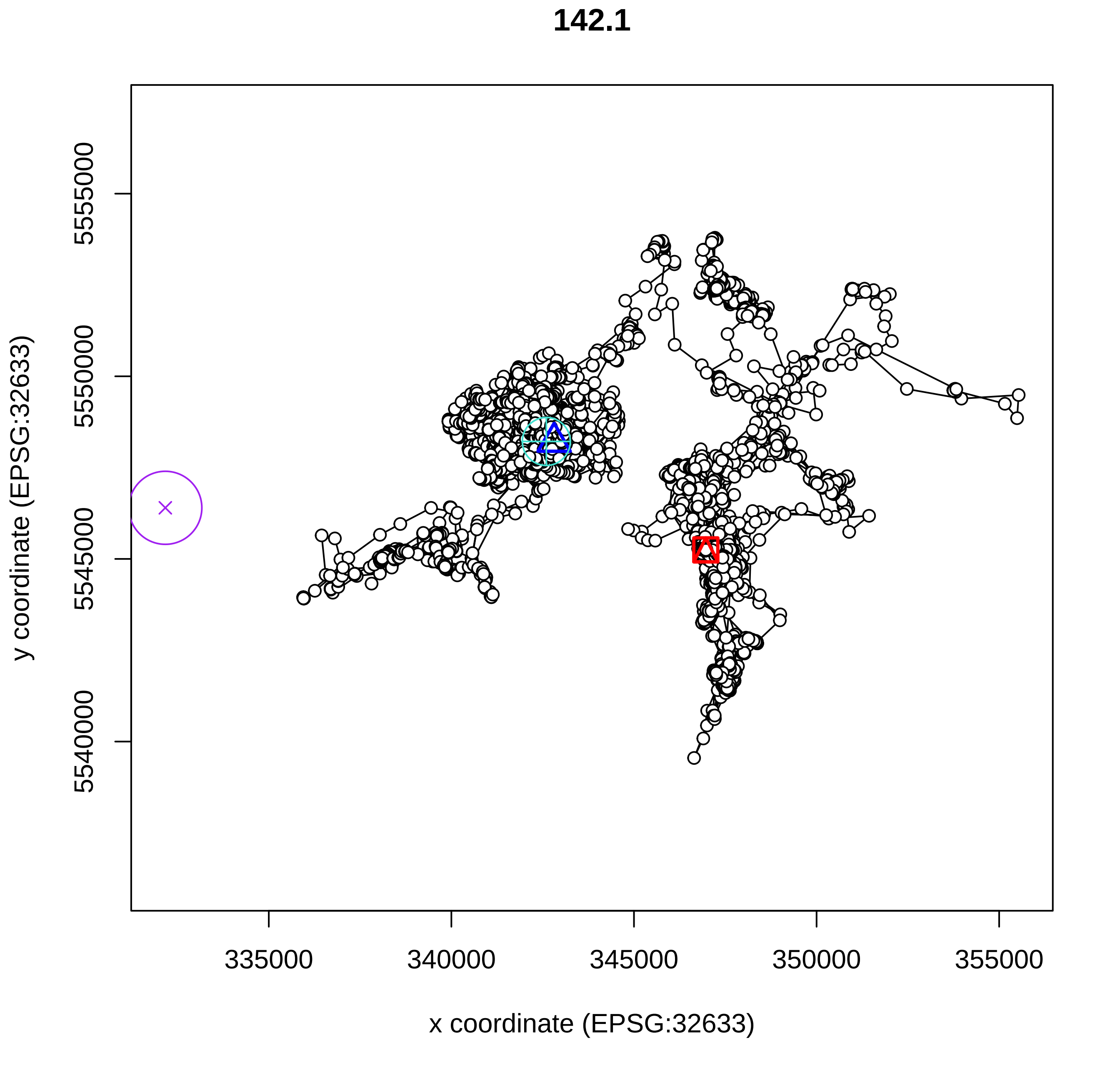
142.1
y coordinate (EPSG:32633)
5540000
5545000
5550000
5555000
x coordinate (EPSG:32633)
335000
340000
345000
350000
355000

144.1

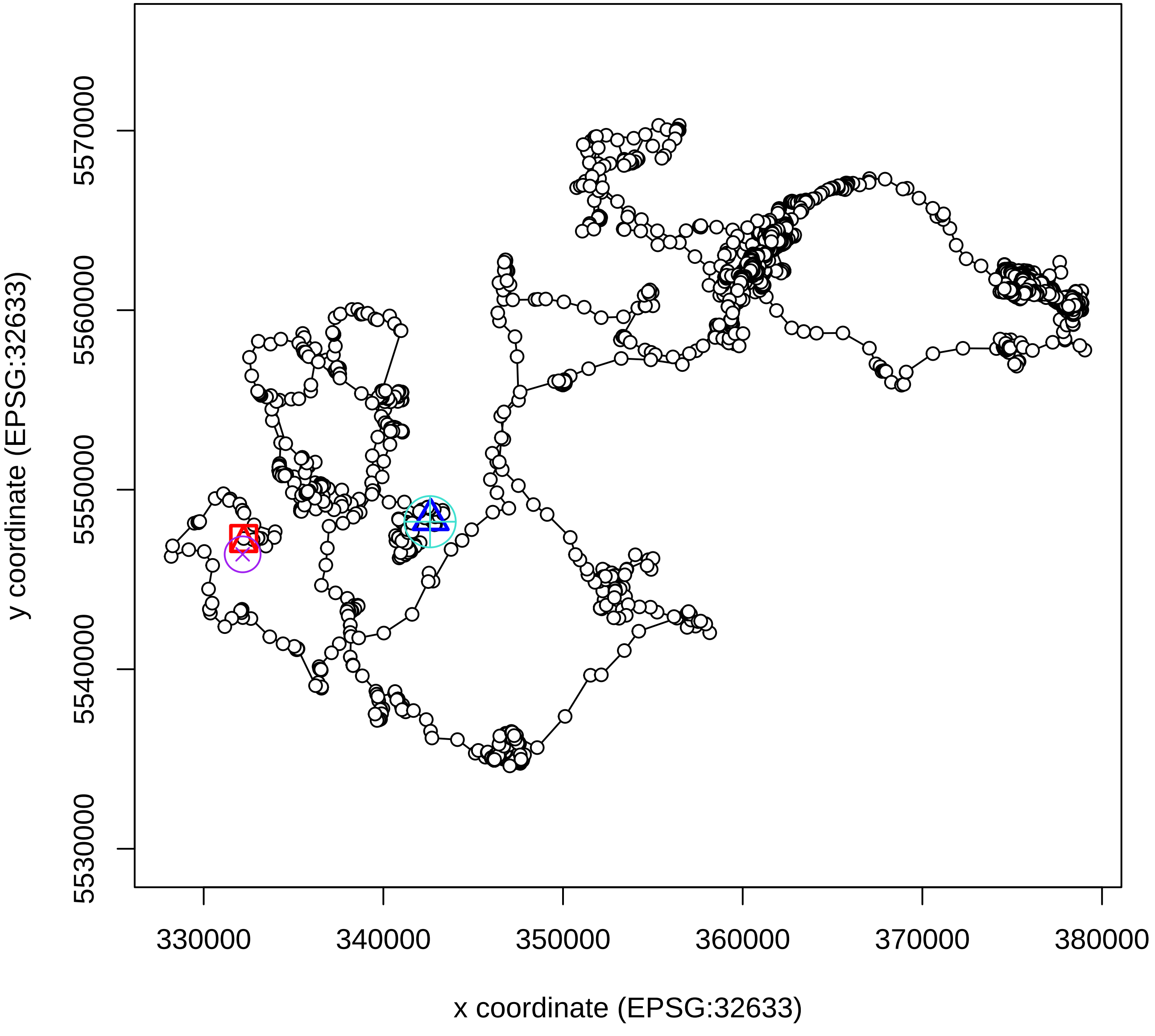

5530000
5540000
5550000
5560000
5570000
y coordinate (EPSG:32633)
330000
340000
350000
360000
370000
380000
x coordinate (EPSG:32633)

145.1

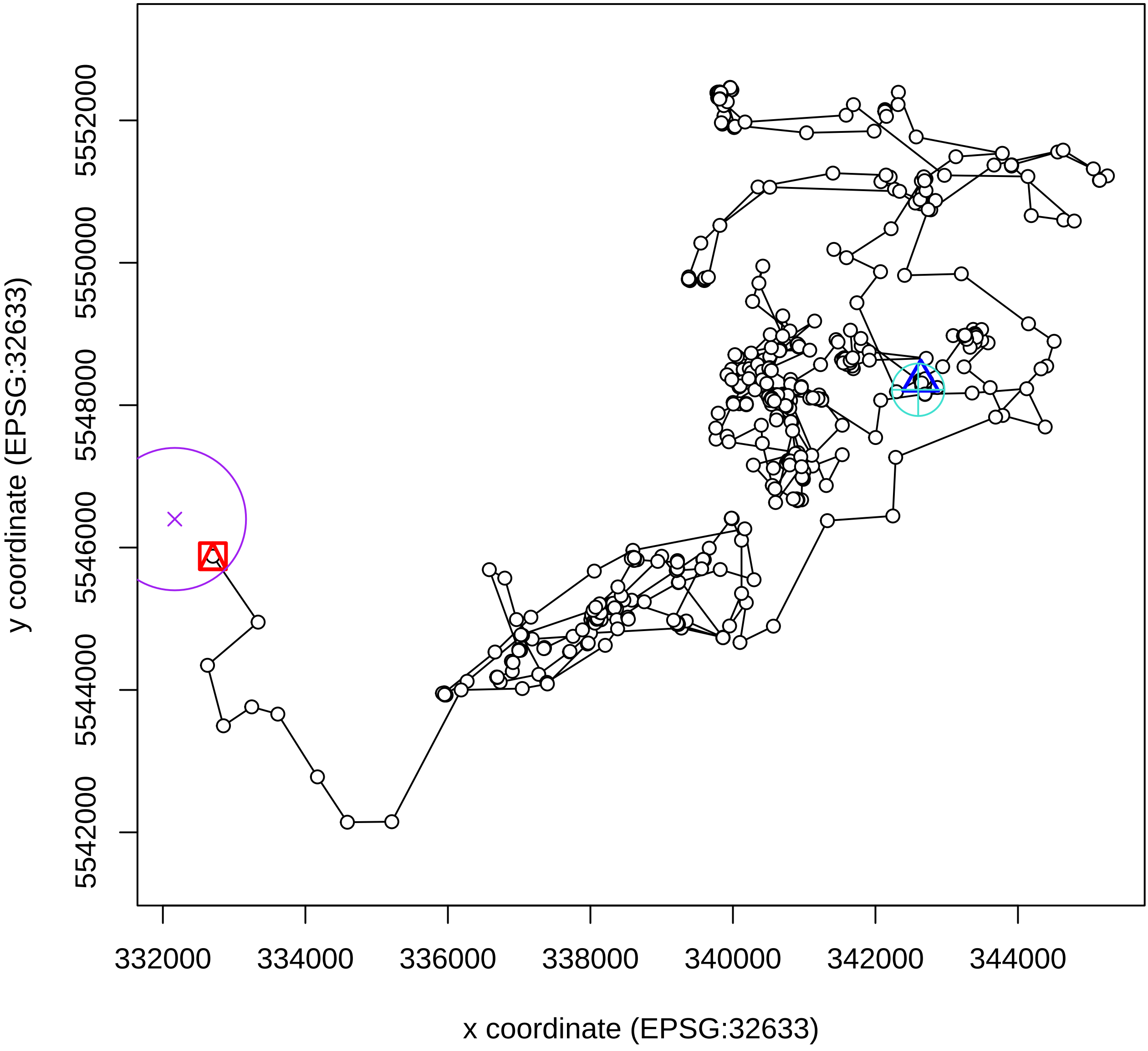

y coordinate (EPSG:32633)
5542000
5544000
5546000
5548000
5550000
5552000
332000
334000
336000
338000
340000
342000
344000
x coordinate (EPSG:32633)

149.1

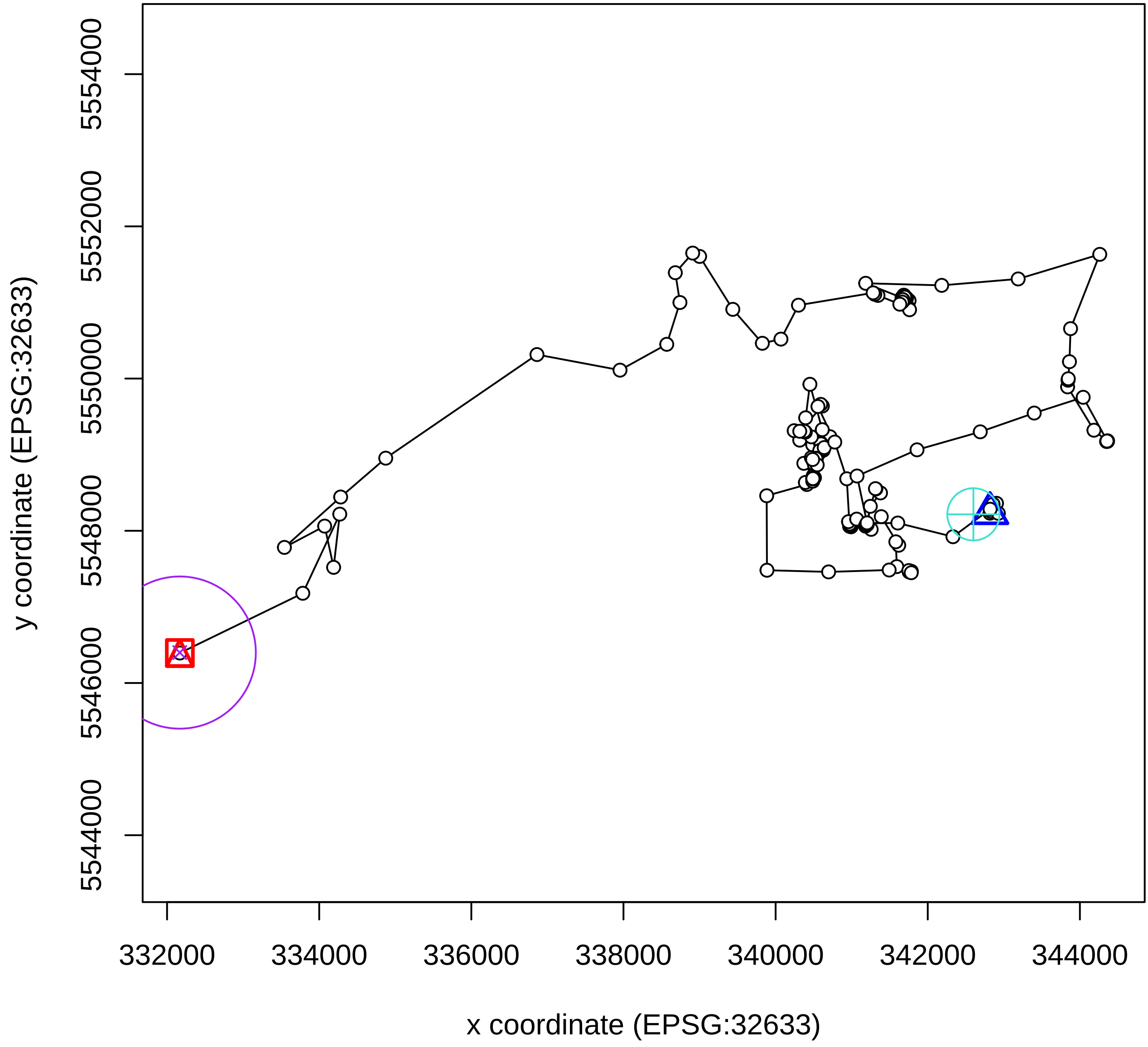

5554000
5552000
5550000
5548000
5546000
5544000
y coordinate (EPSG:32633)
332000
334000
336000
338000
340000
342000
344000
x coordinate (EPSG:32633)

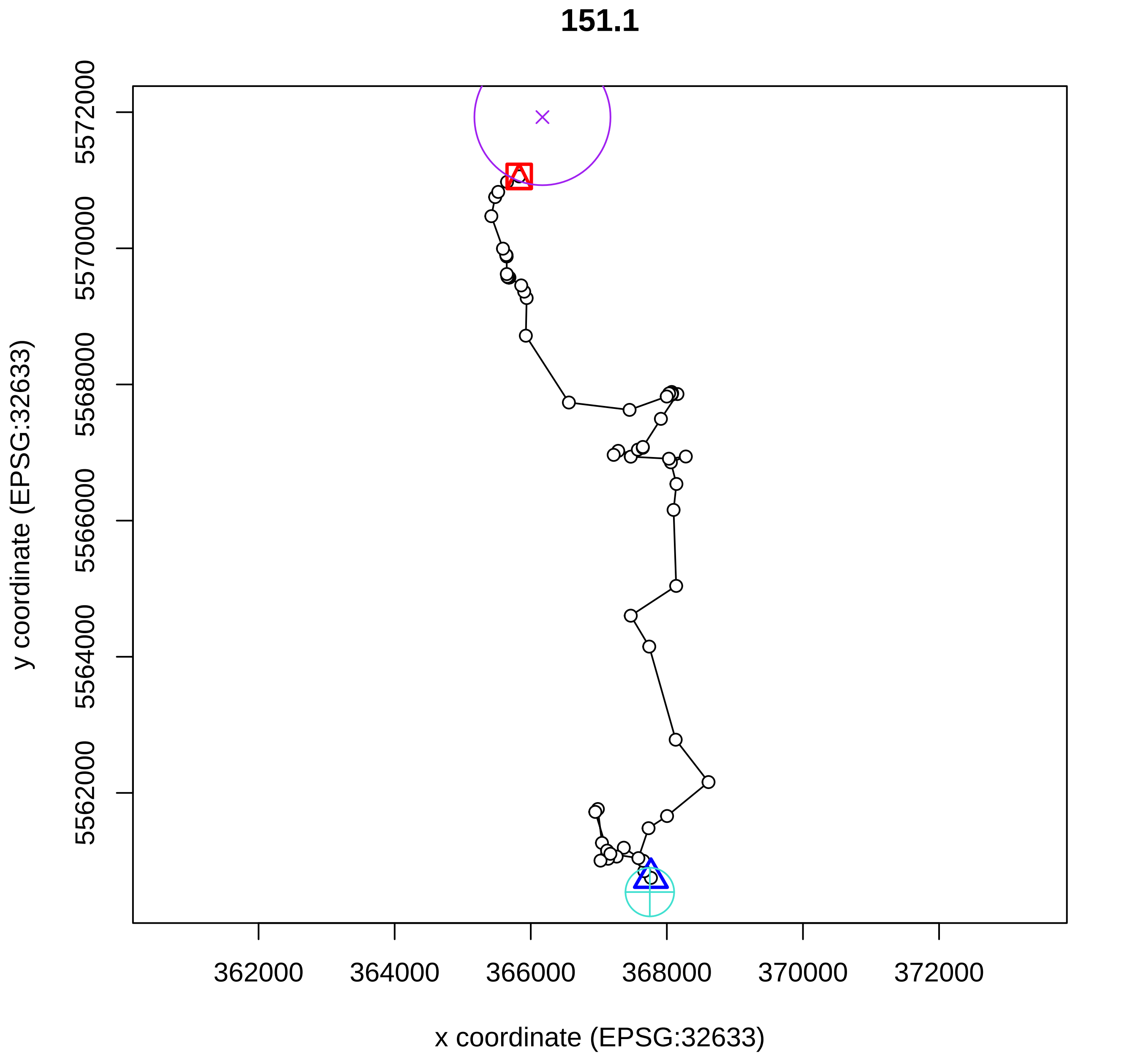
151.1
y coordinate (EPSG:32633)
5562000
5564000
5566000
5568000
5570000
5572000
362000
364000
366000
368000
370000
372000
x coordinate (EPSG:32633)
