# Supplementary material for: A GPS assisted translocation experiment to study the homing behavior of red deer
Source: Sci Rep. 2024 Mar 21;14:6770. doi: 10.1038/s41598-024-56951-0 (PMC10958021; doi:10.1038/s41598-024-56951-0)

**Map Information**

Open purple circle: Home area

Purple dot: Release location

Light-blue track: segment 1

Light-red track: segment 2

Black: segment 3

Background map from by Stamen Design, under CC BY 3.0. Data by OpenStreetMap. The site name is given in the map header as well as the ID of the animal and testing round (number before decimal point is the ID number, after is the testing round).

104.1, site: Doupov

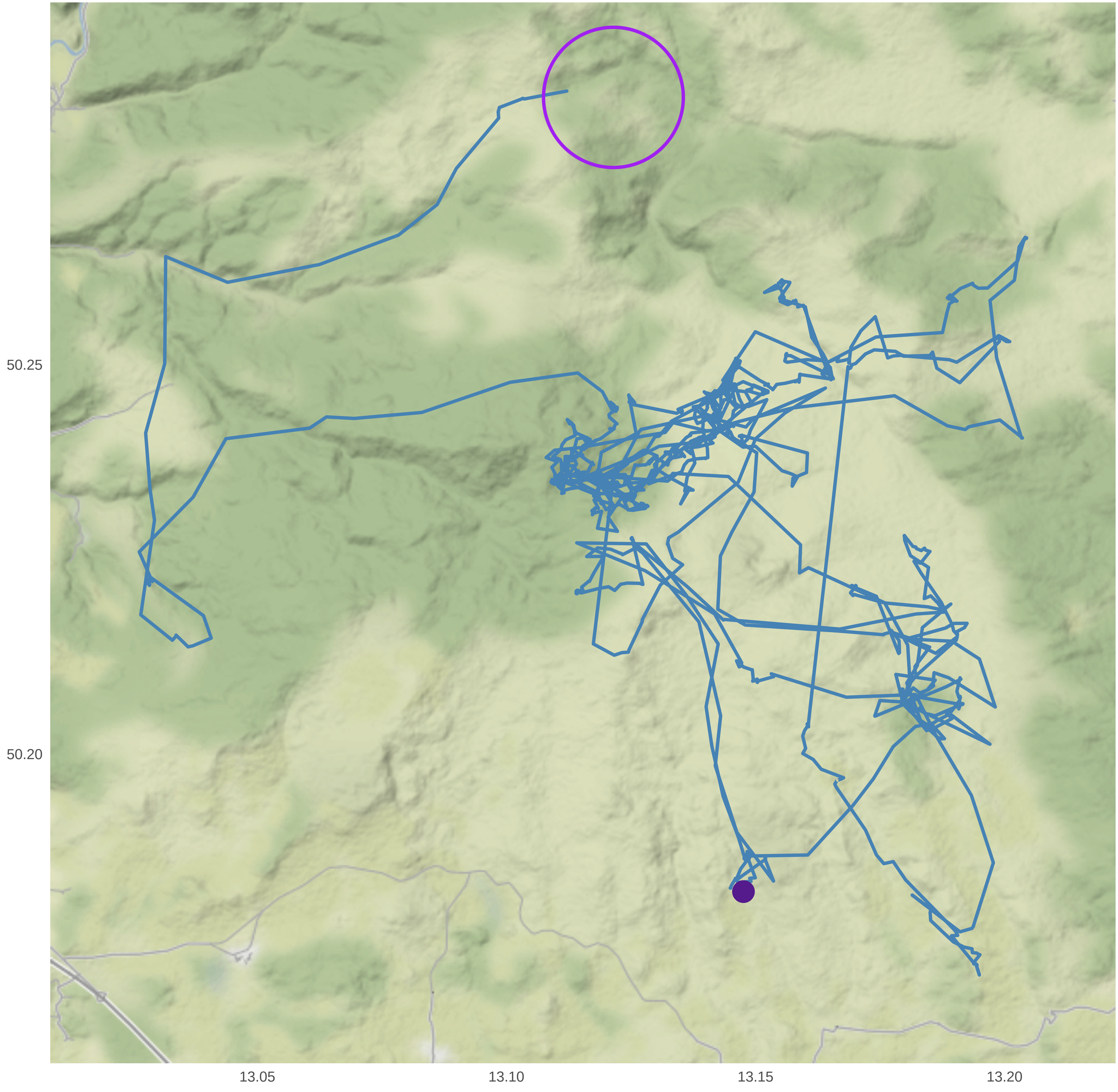

104.2, site: Doupov

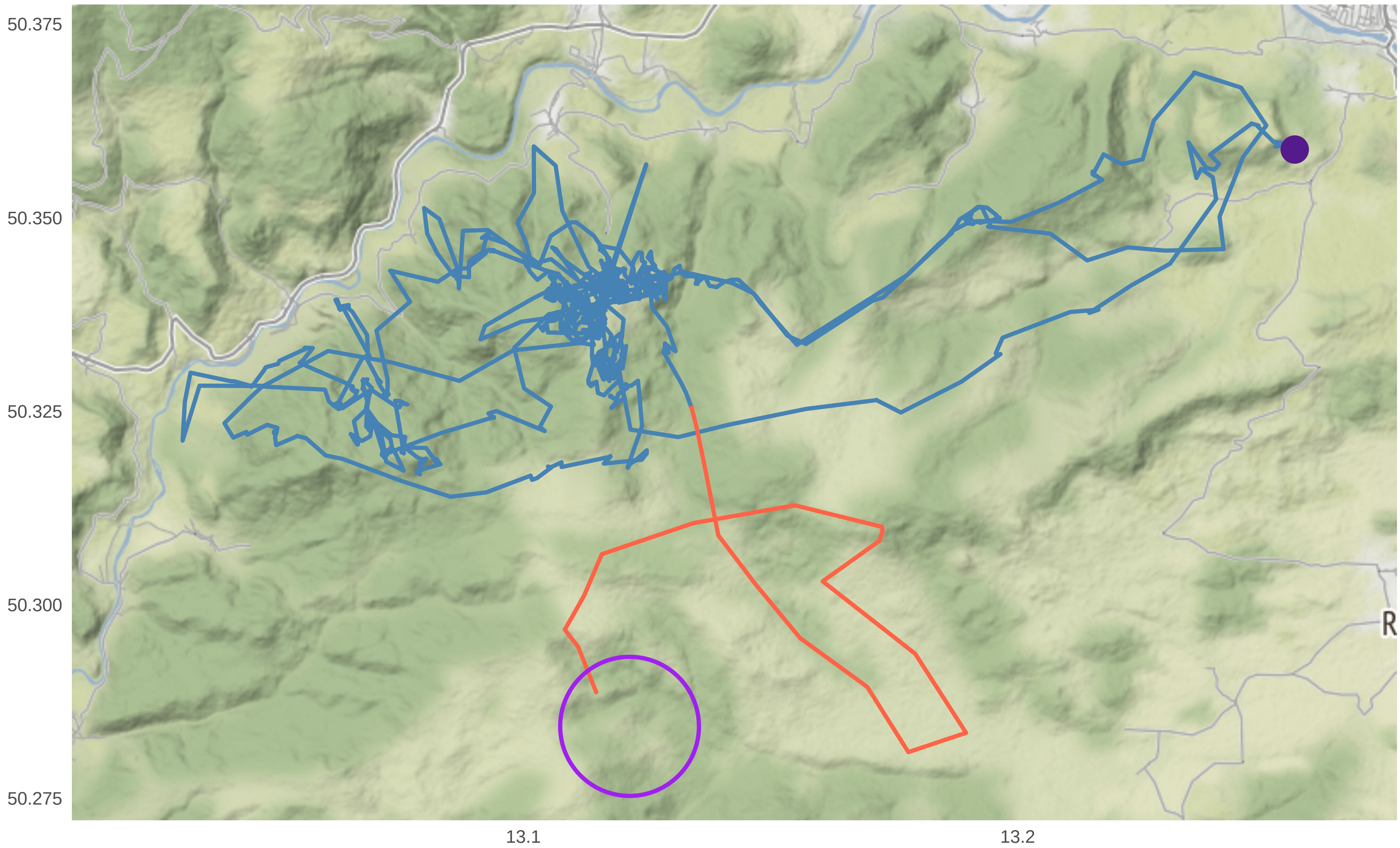

105.1, site: Kladska

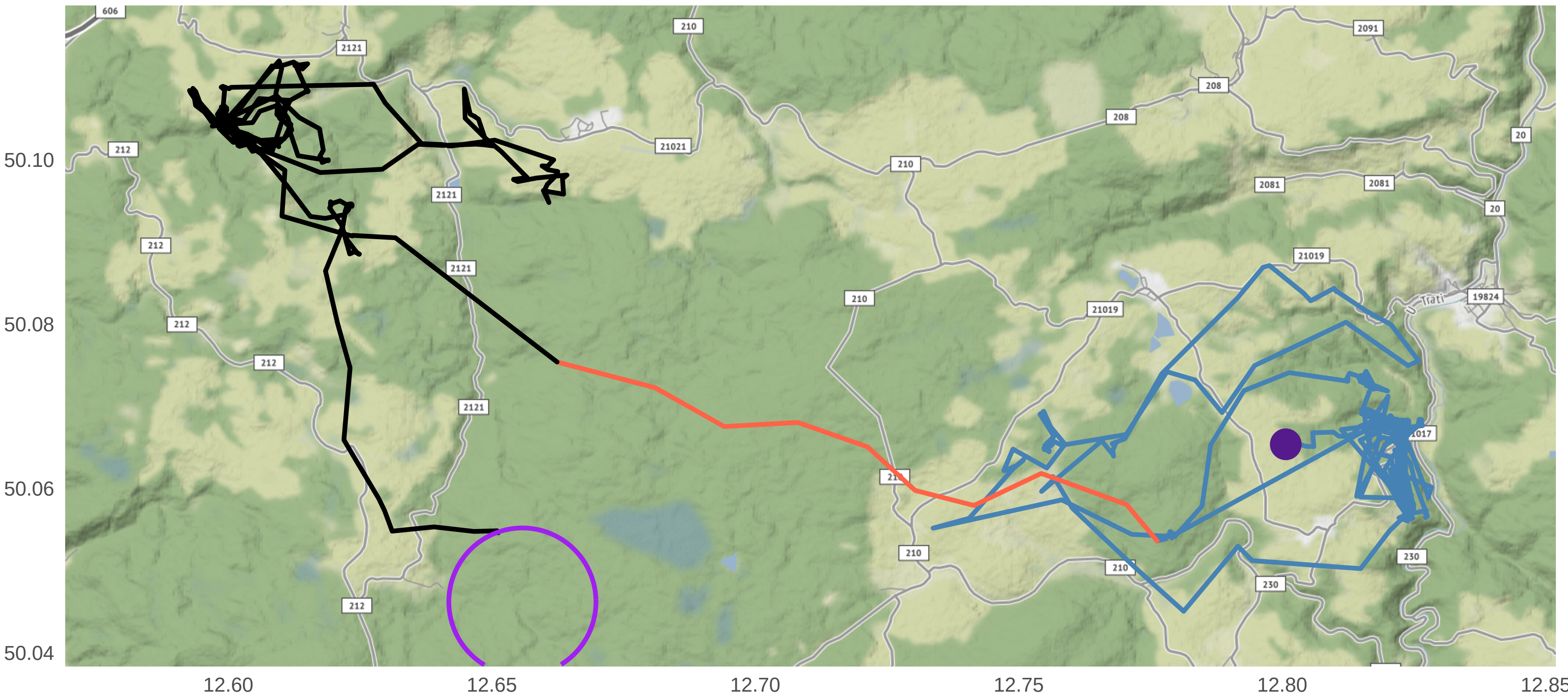

105.2, site: Kladska

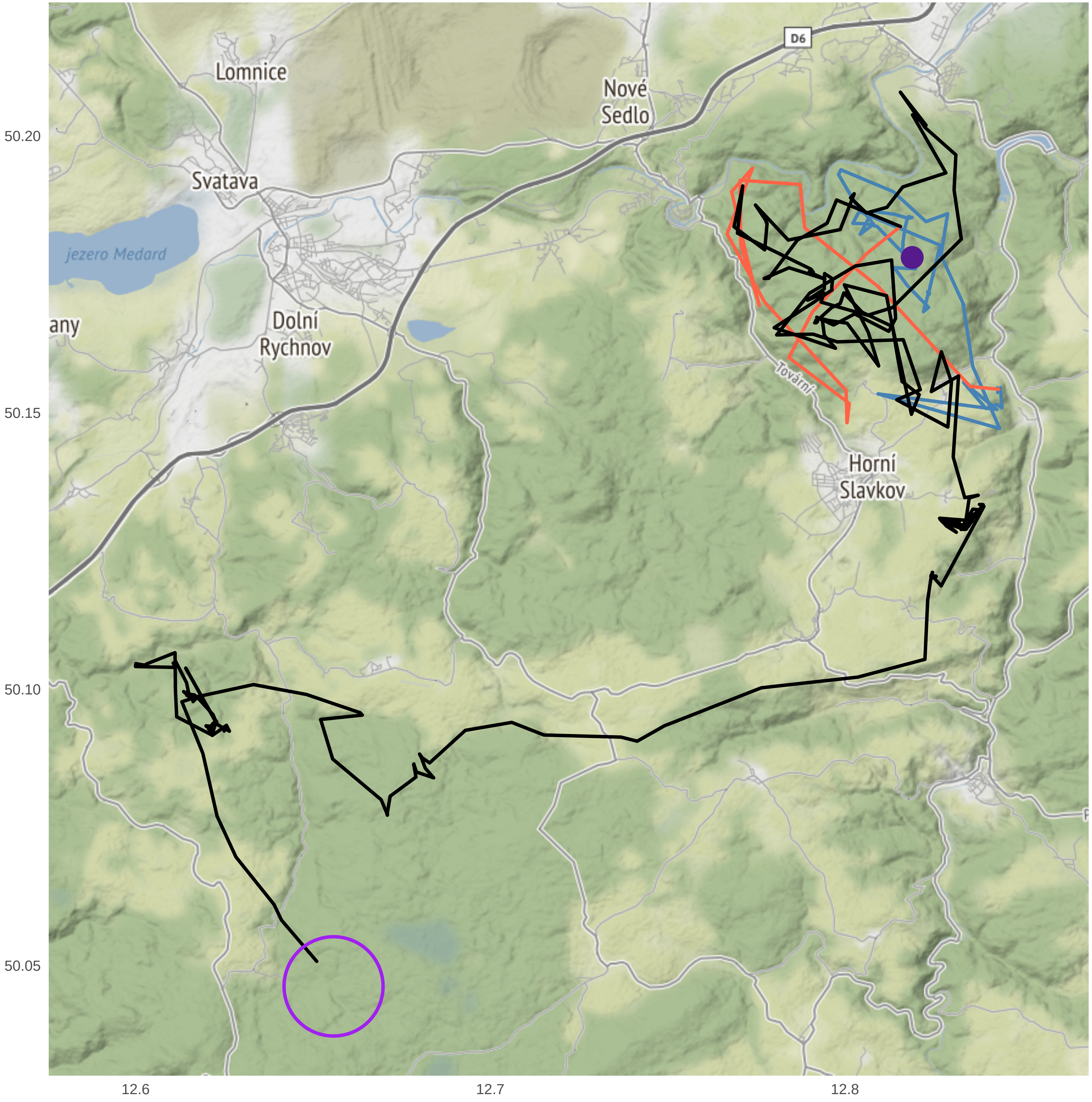

107.1, site: Doupov

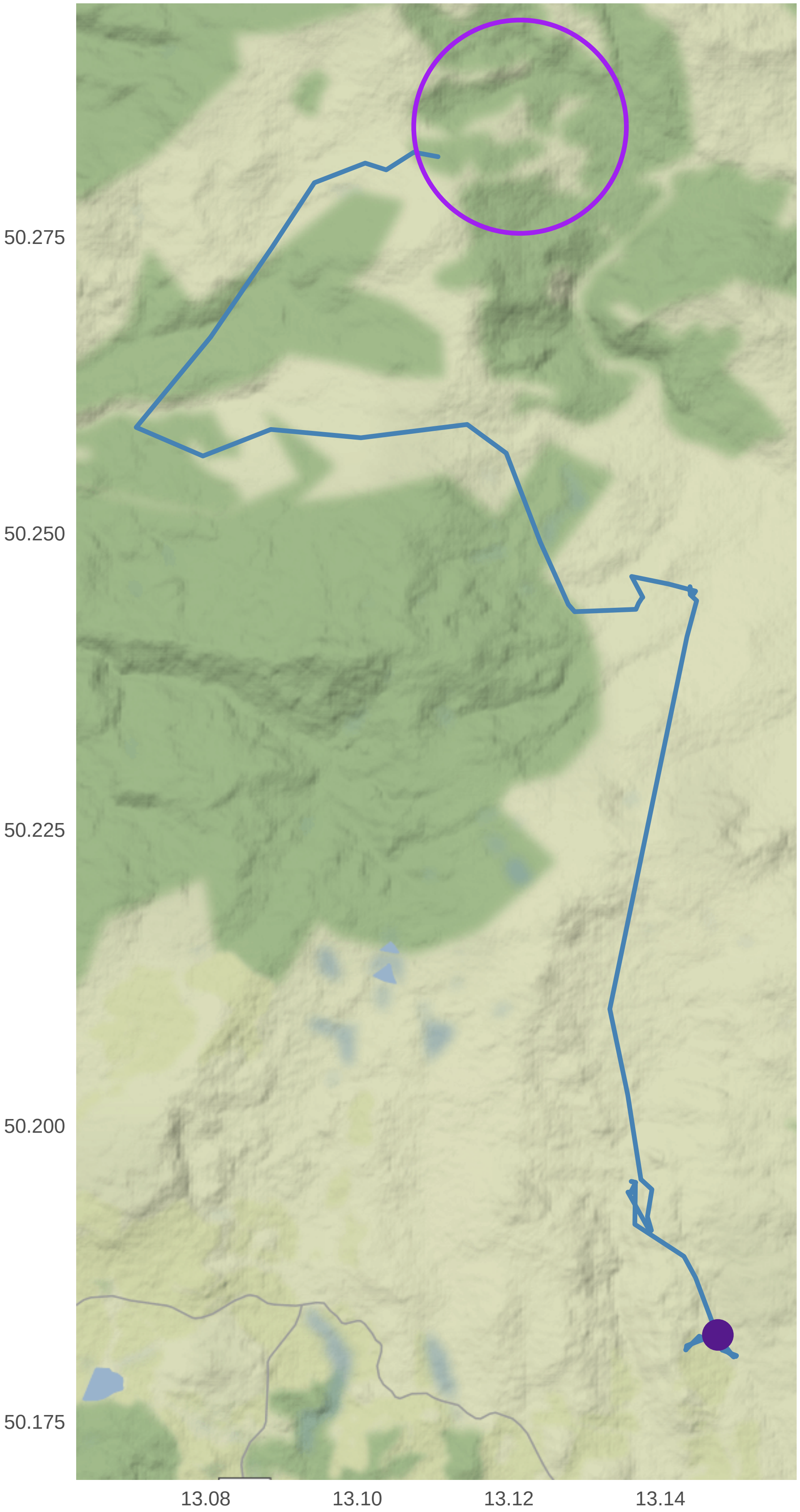

107.2, site: Doupov

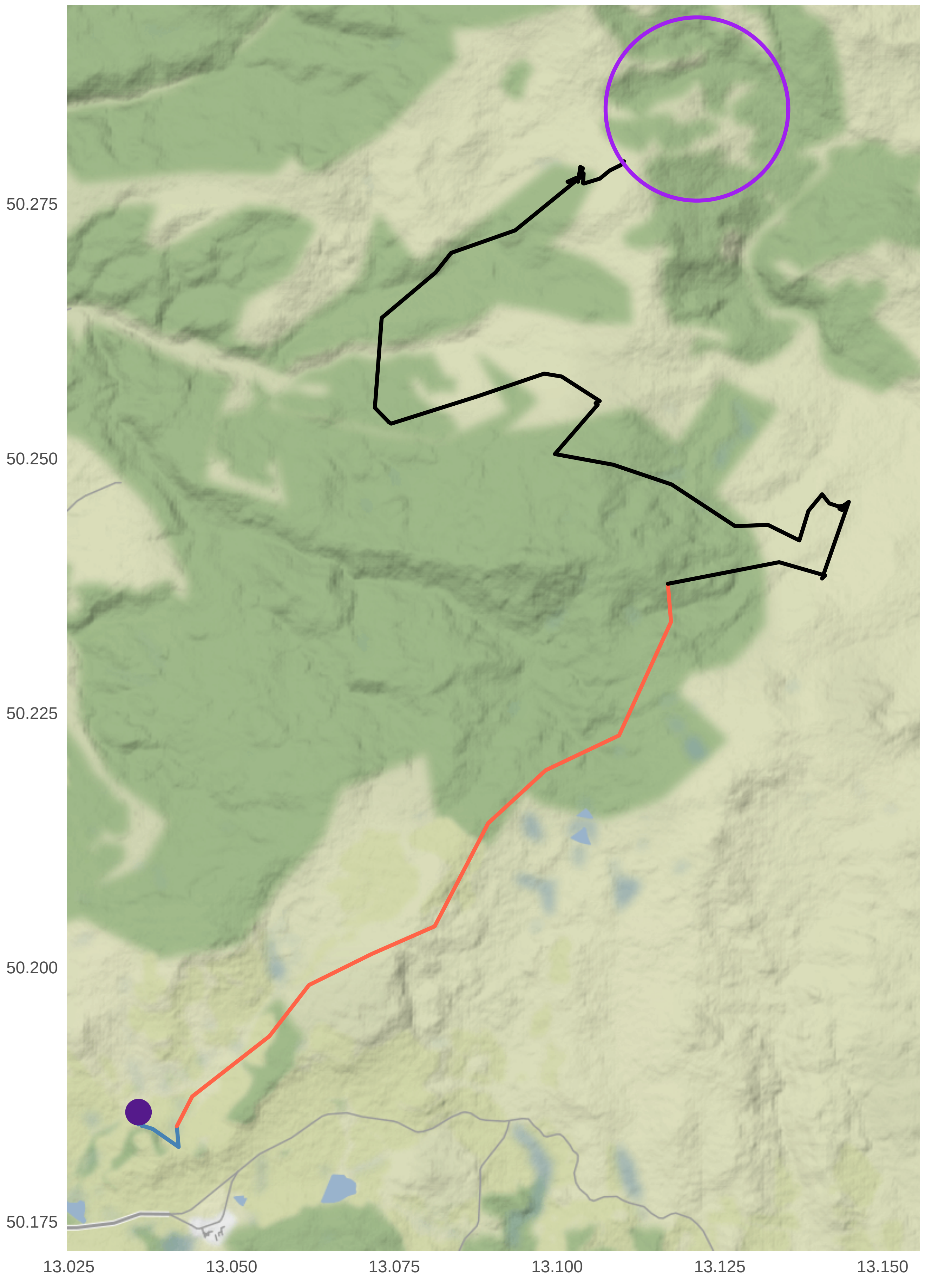

107.3, site: Doupov

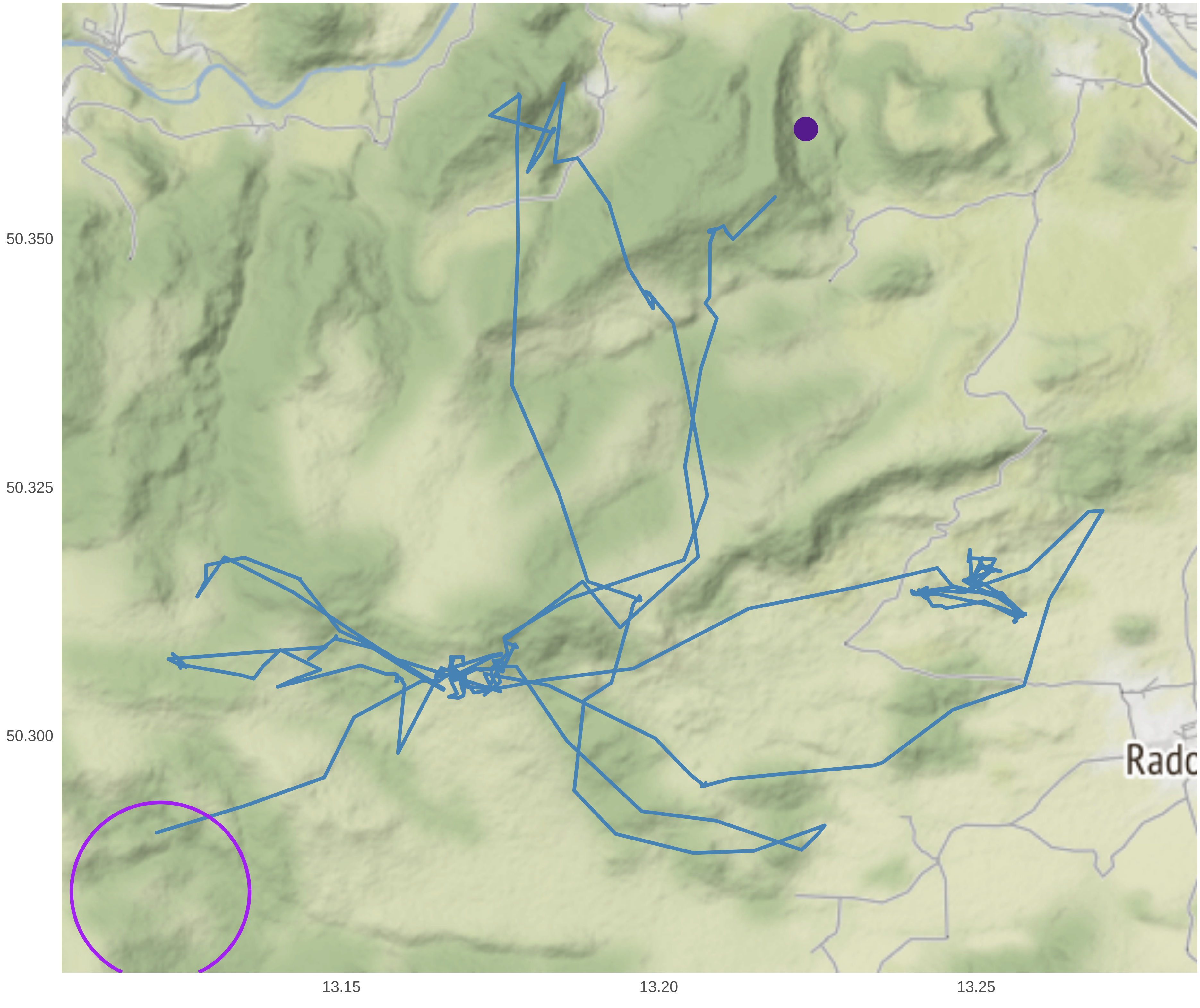

108.1, site: Doupov

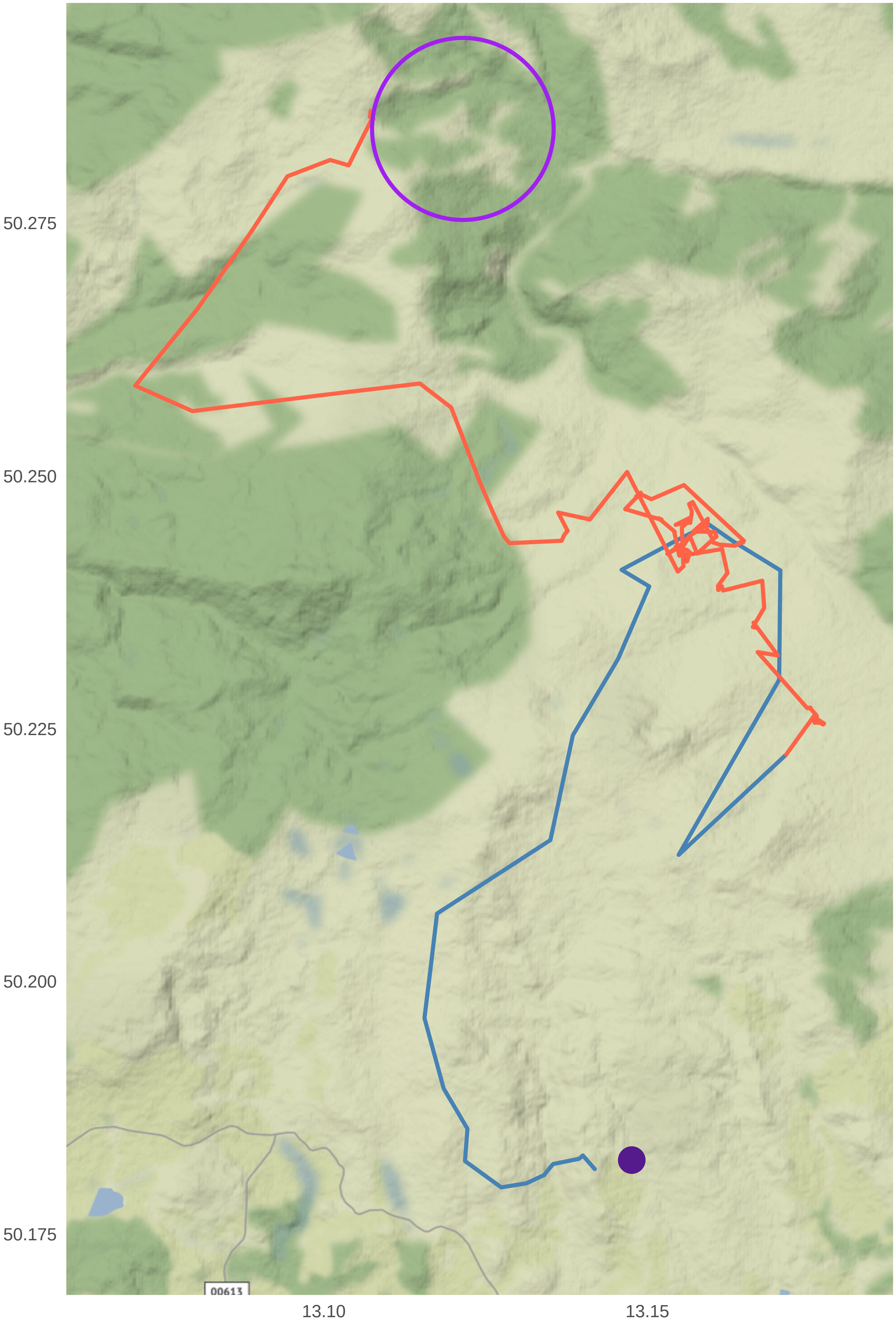

108.2, site: Doupov

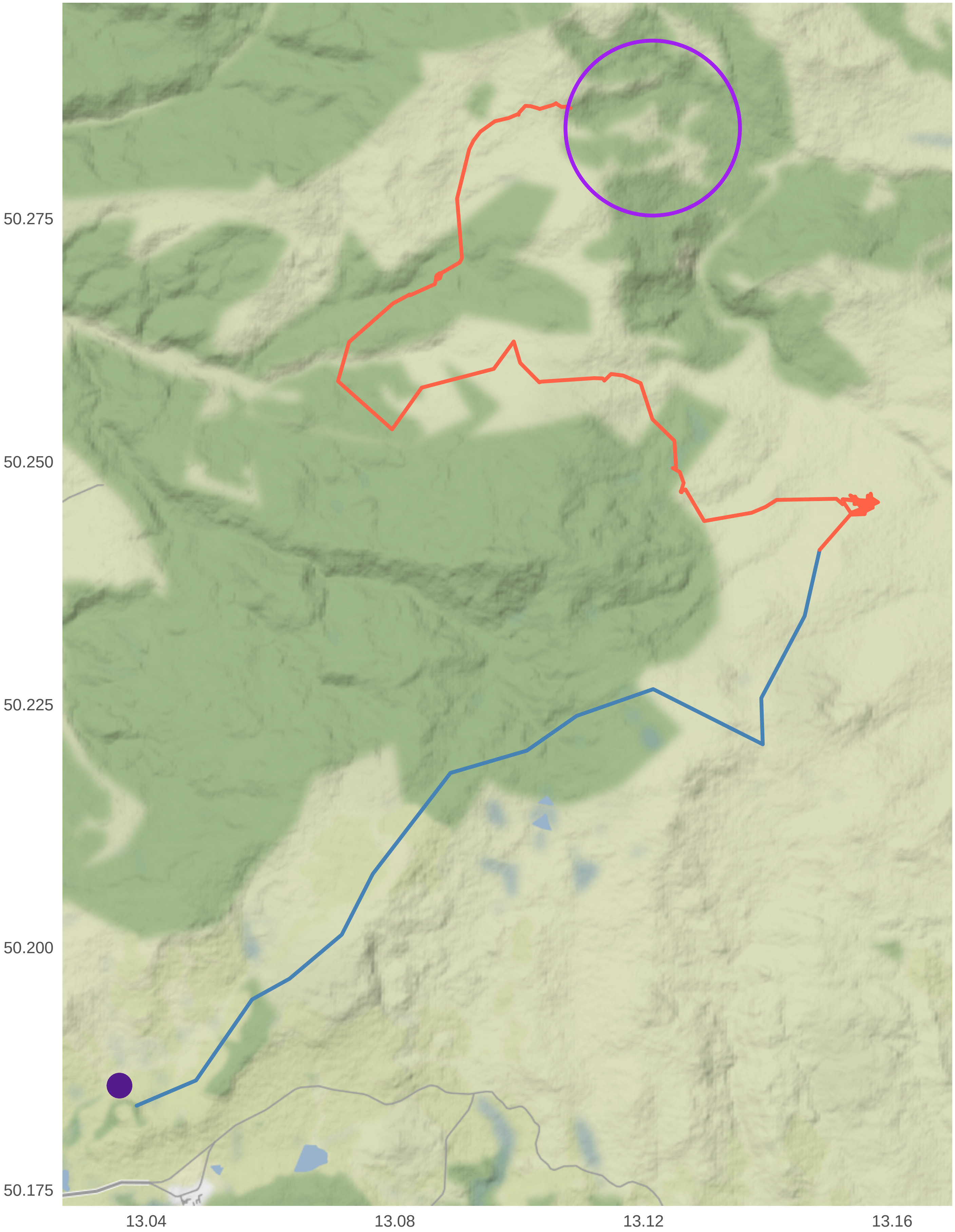

108.3, site: Doupov

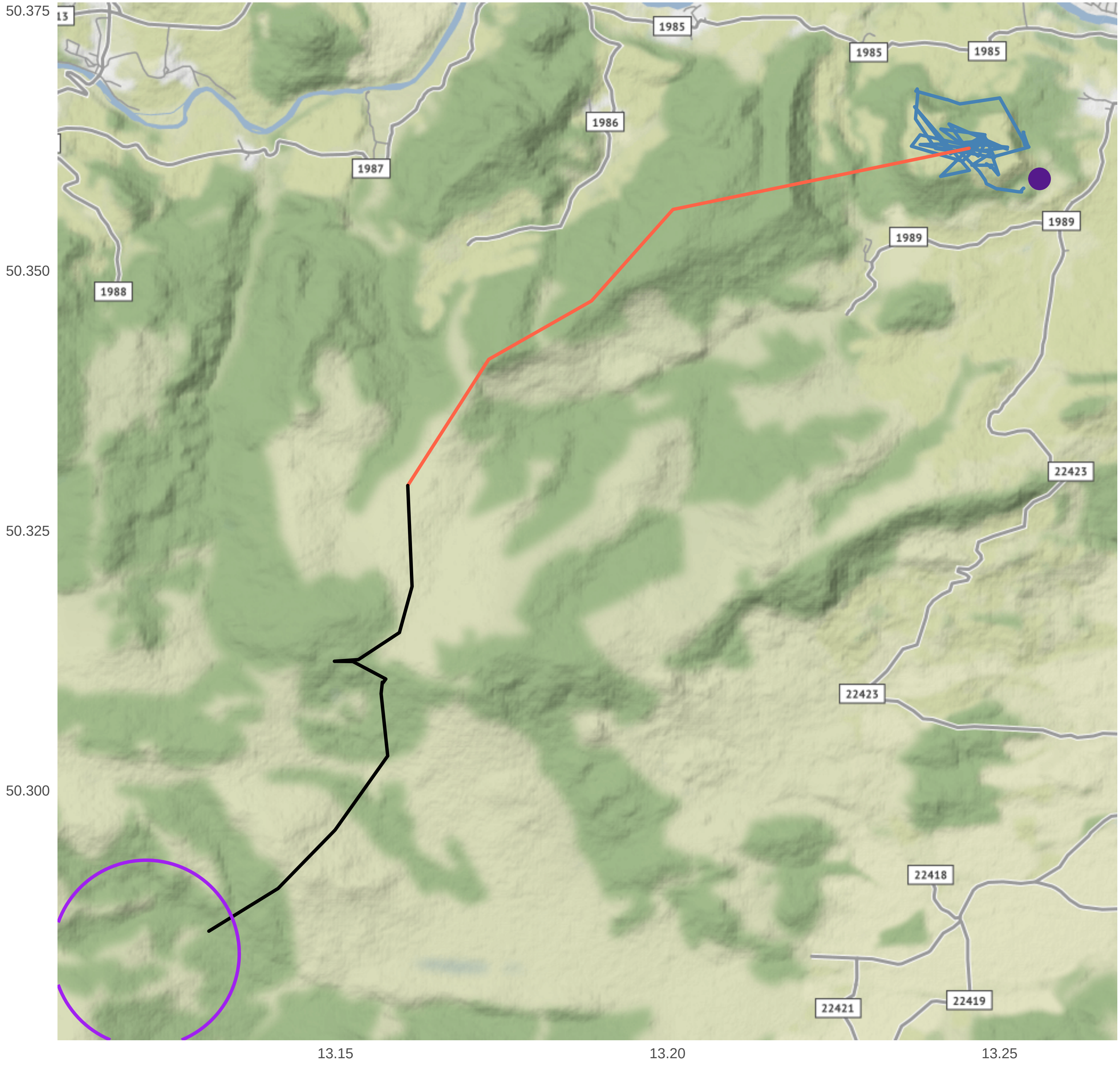

110.1, site: Kladska

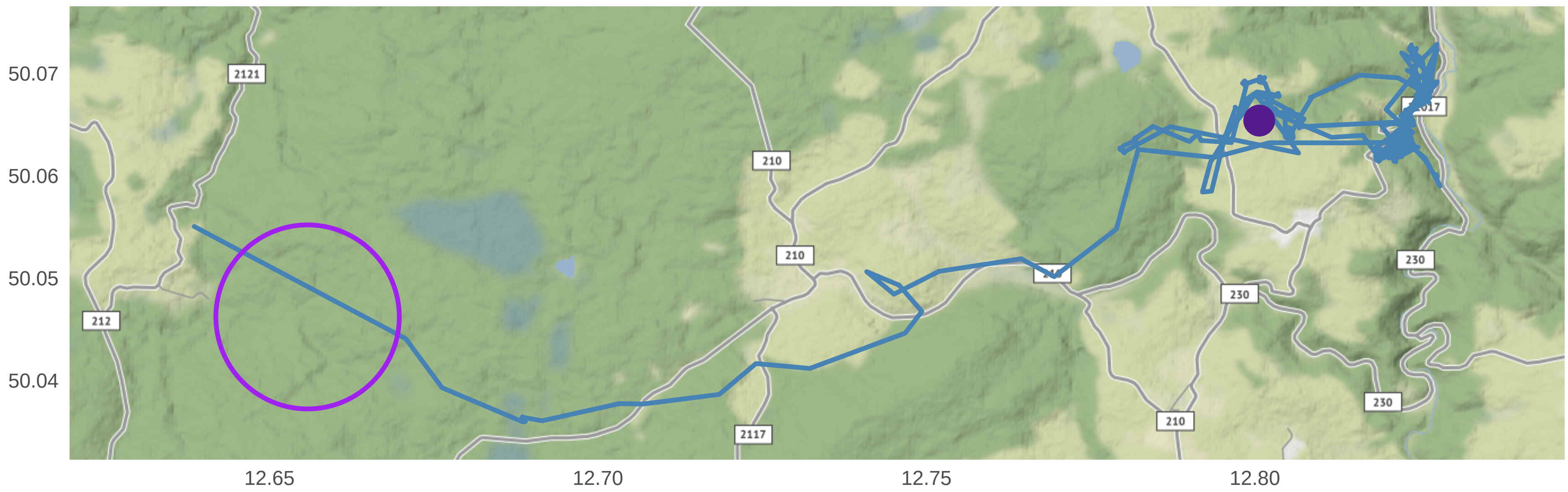

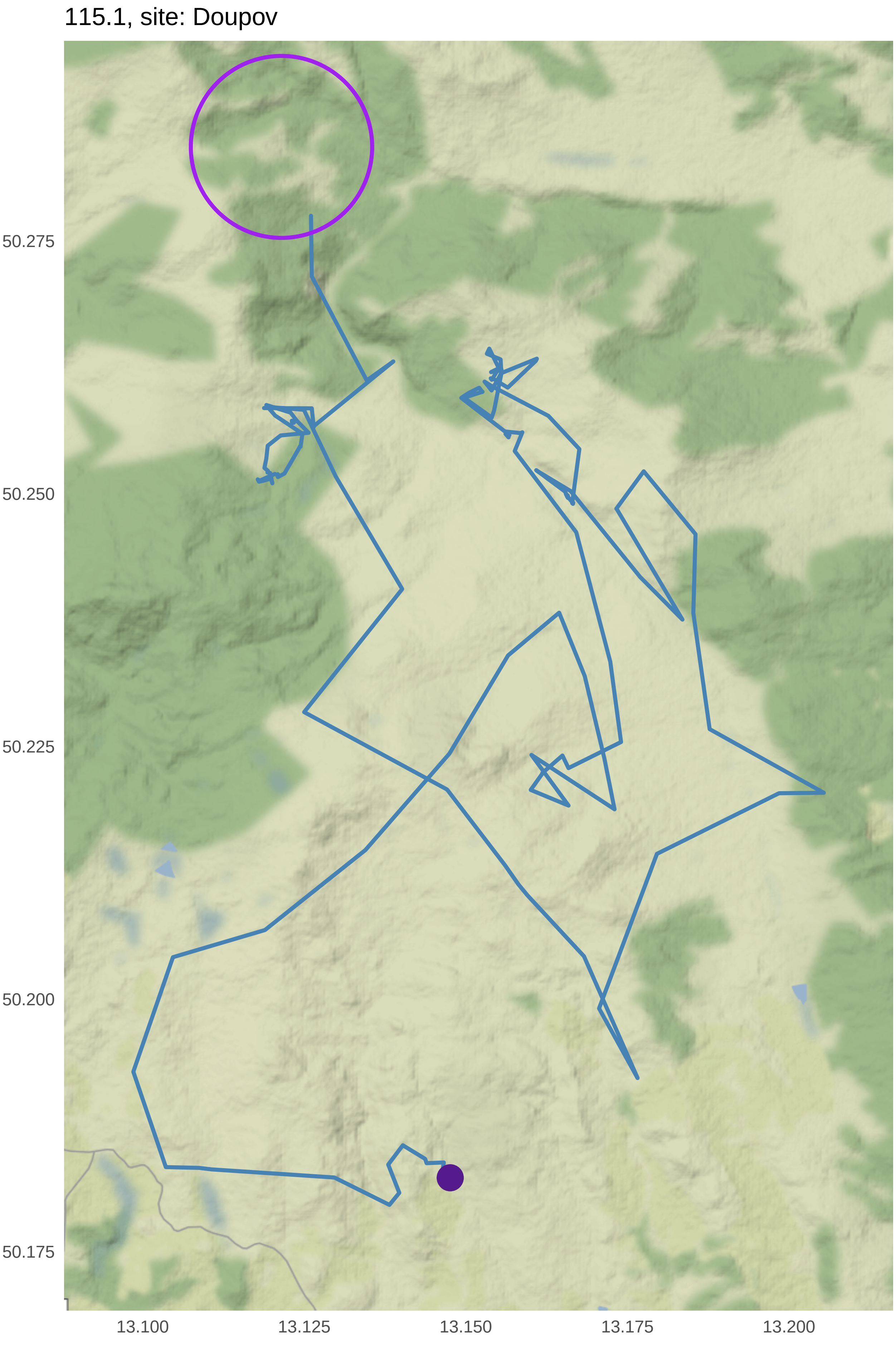
115.1, site: Doupov
50.275
50.250
50.225
50.200
50.175
13.100
13.125
13.150
13.175
13.200

115.2, site: Doupov

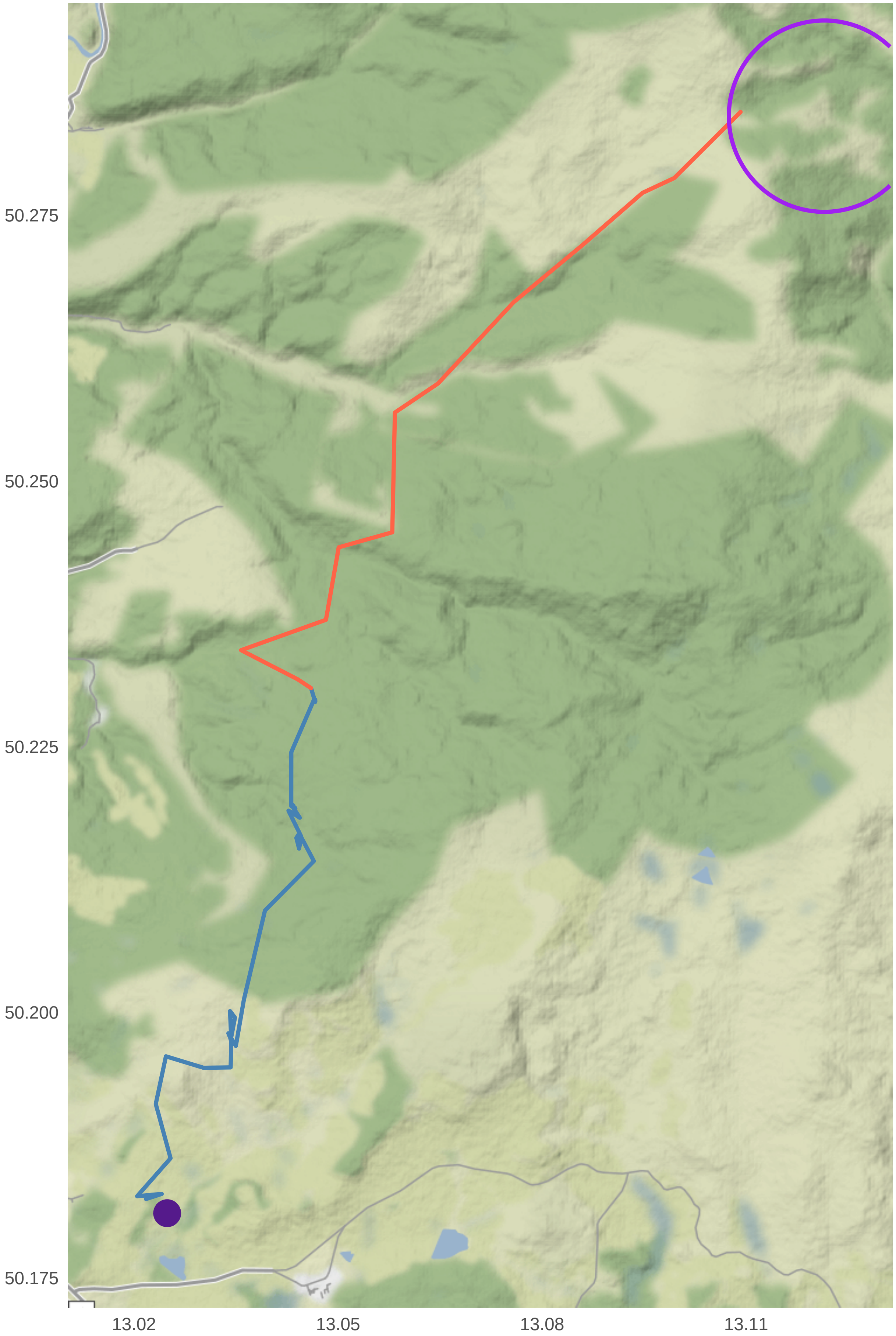

117.1, site: Doupov

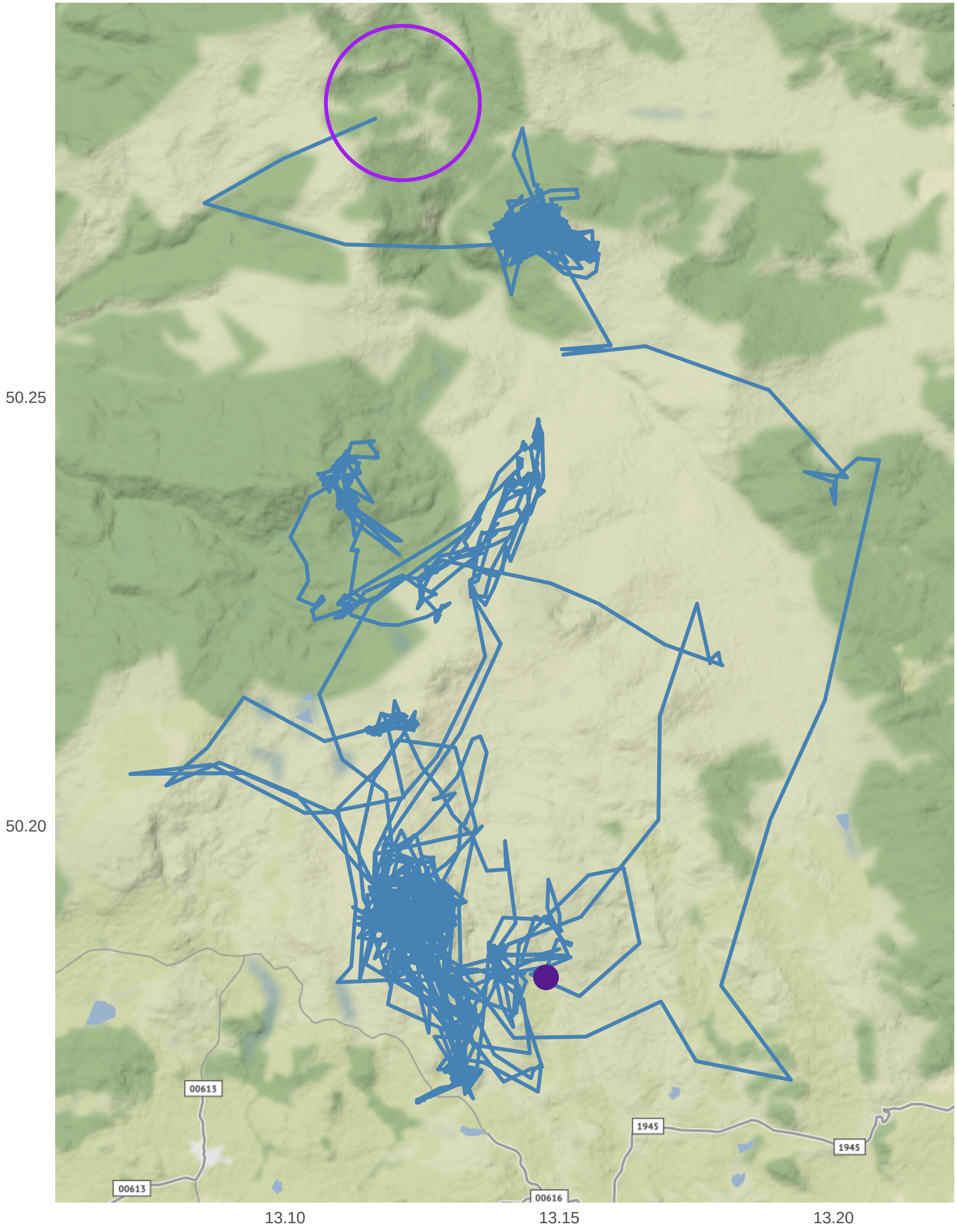

117.2, site: Doupov

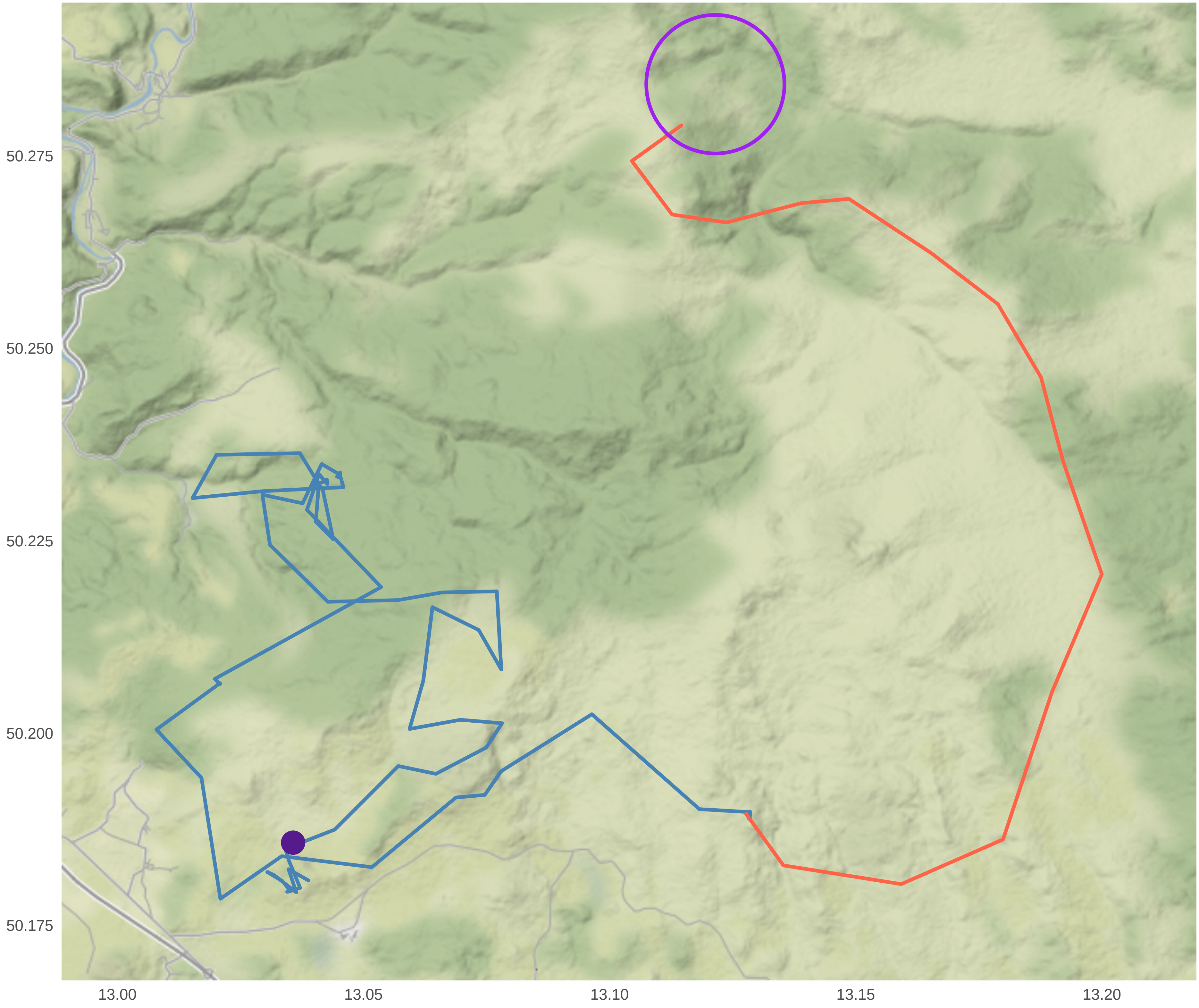

118.1, site: Doupov

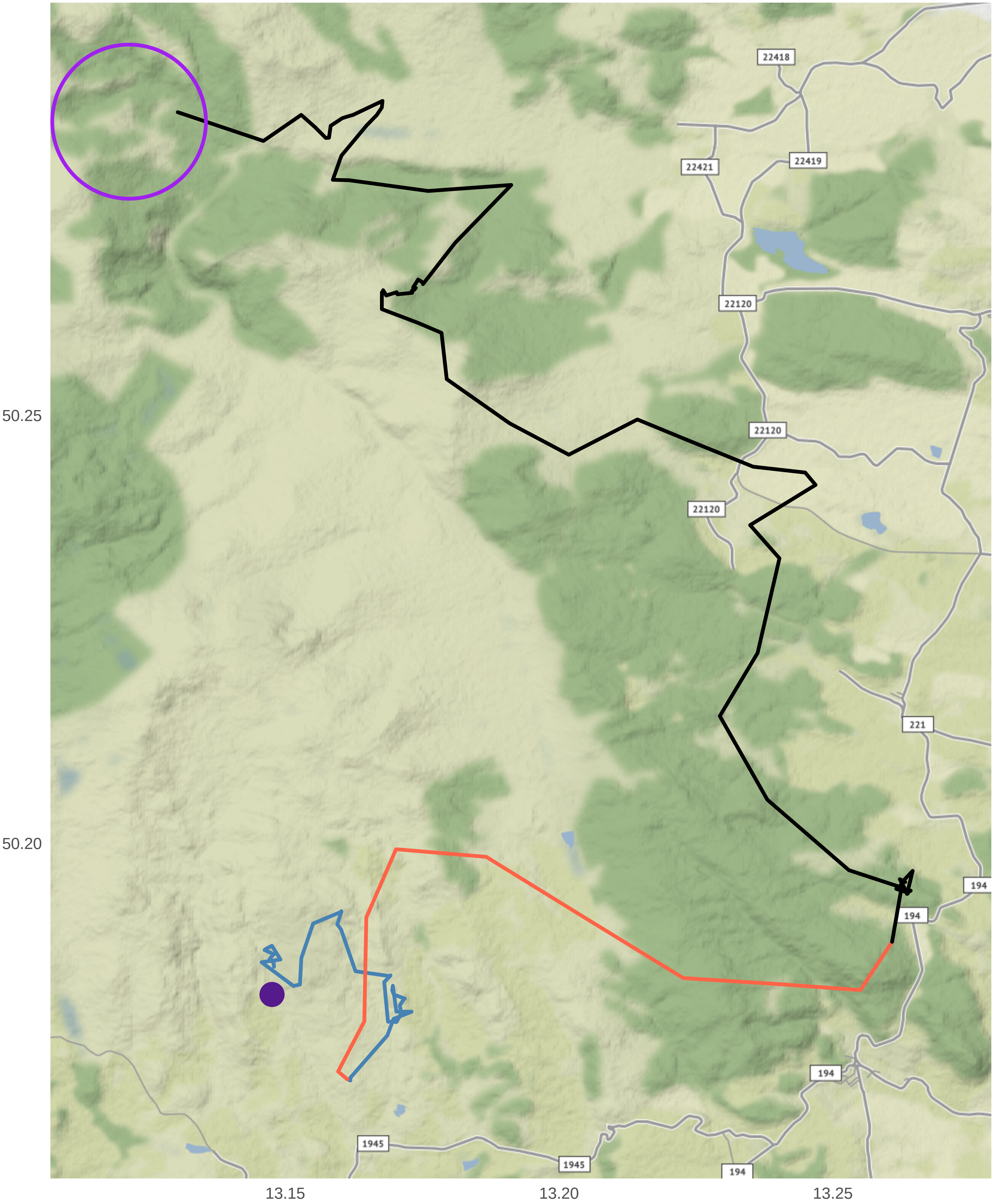

120.1, site: Doupov

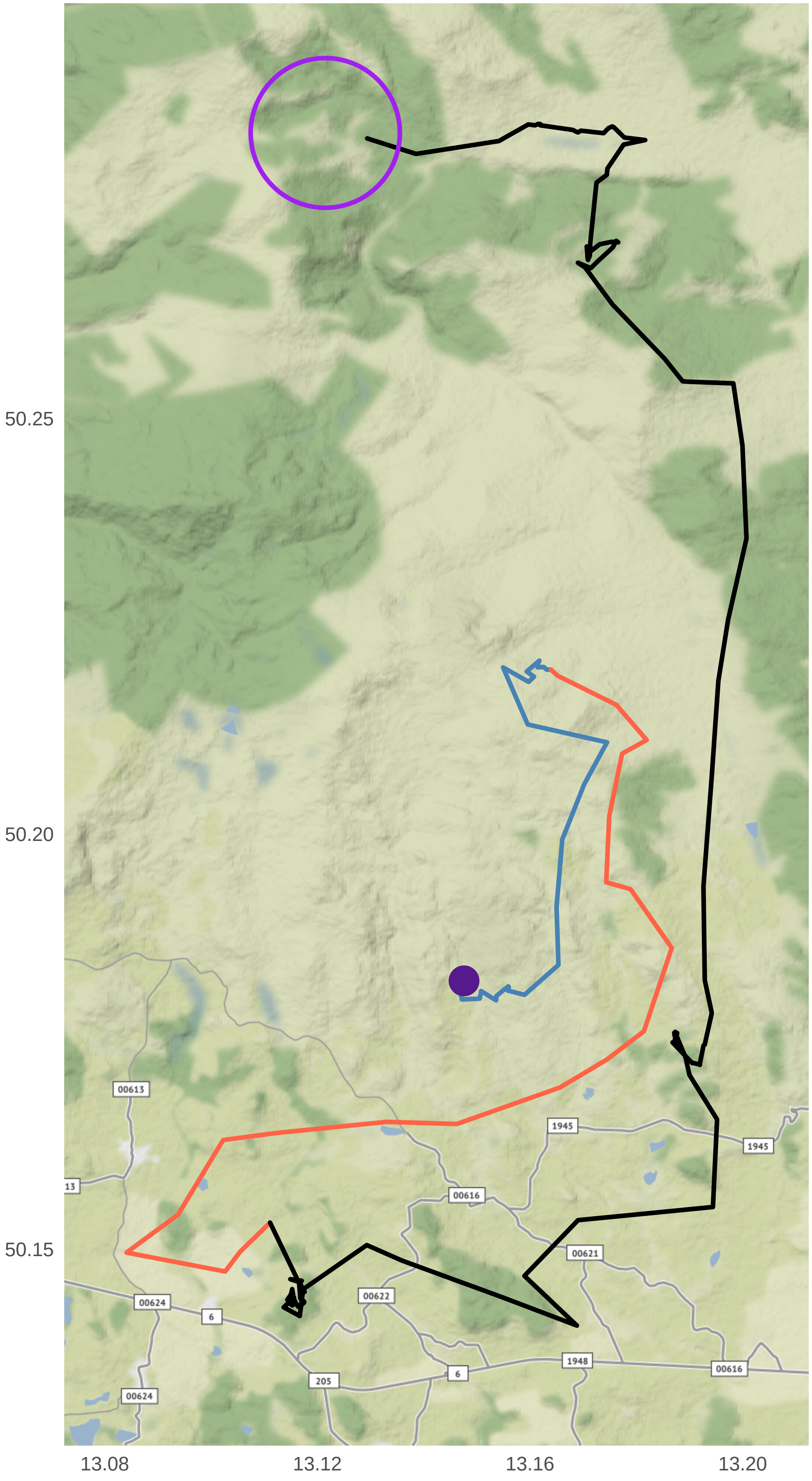

120.2, site: Doupov

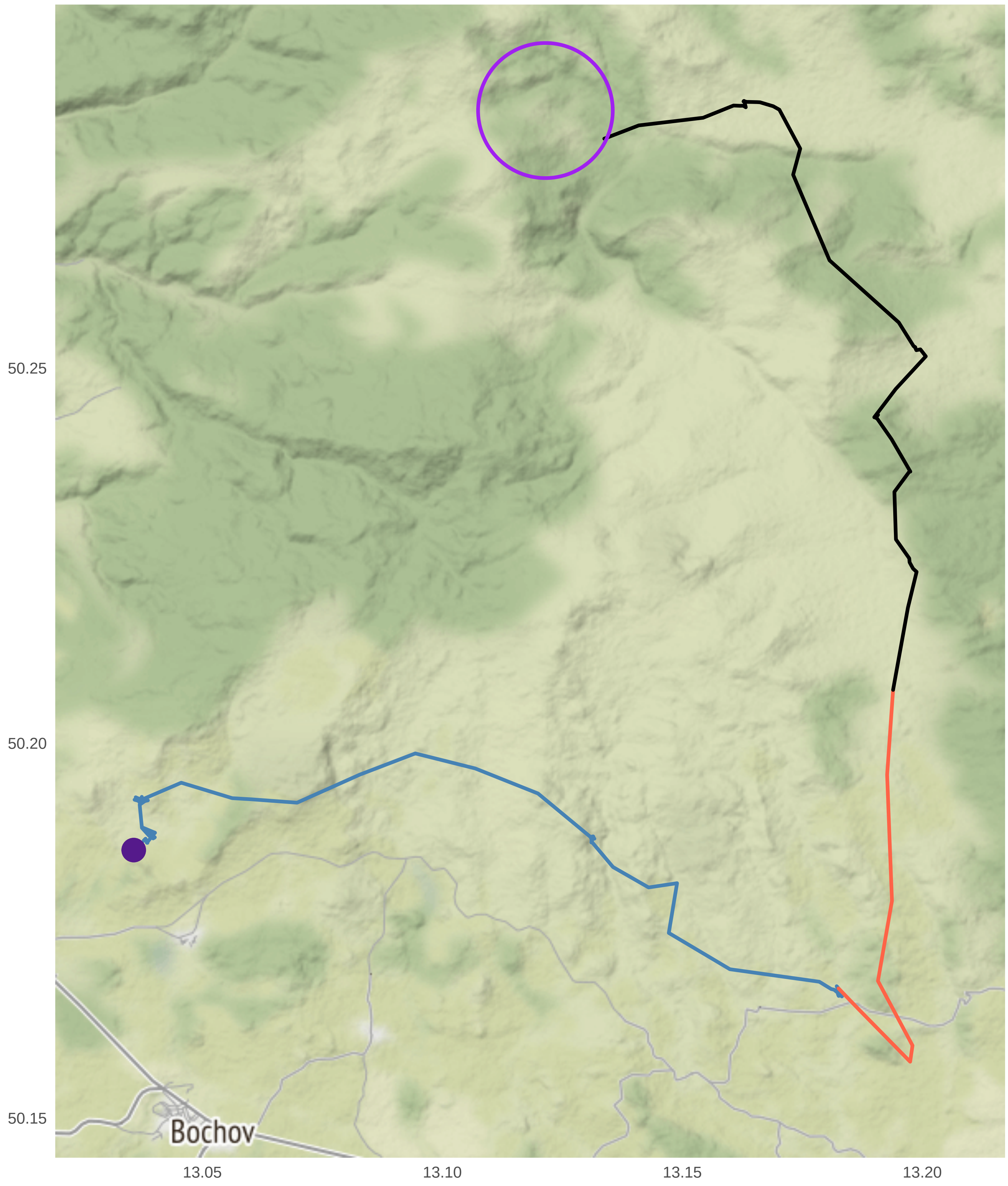

126.1, site: Kladska

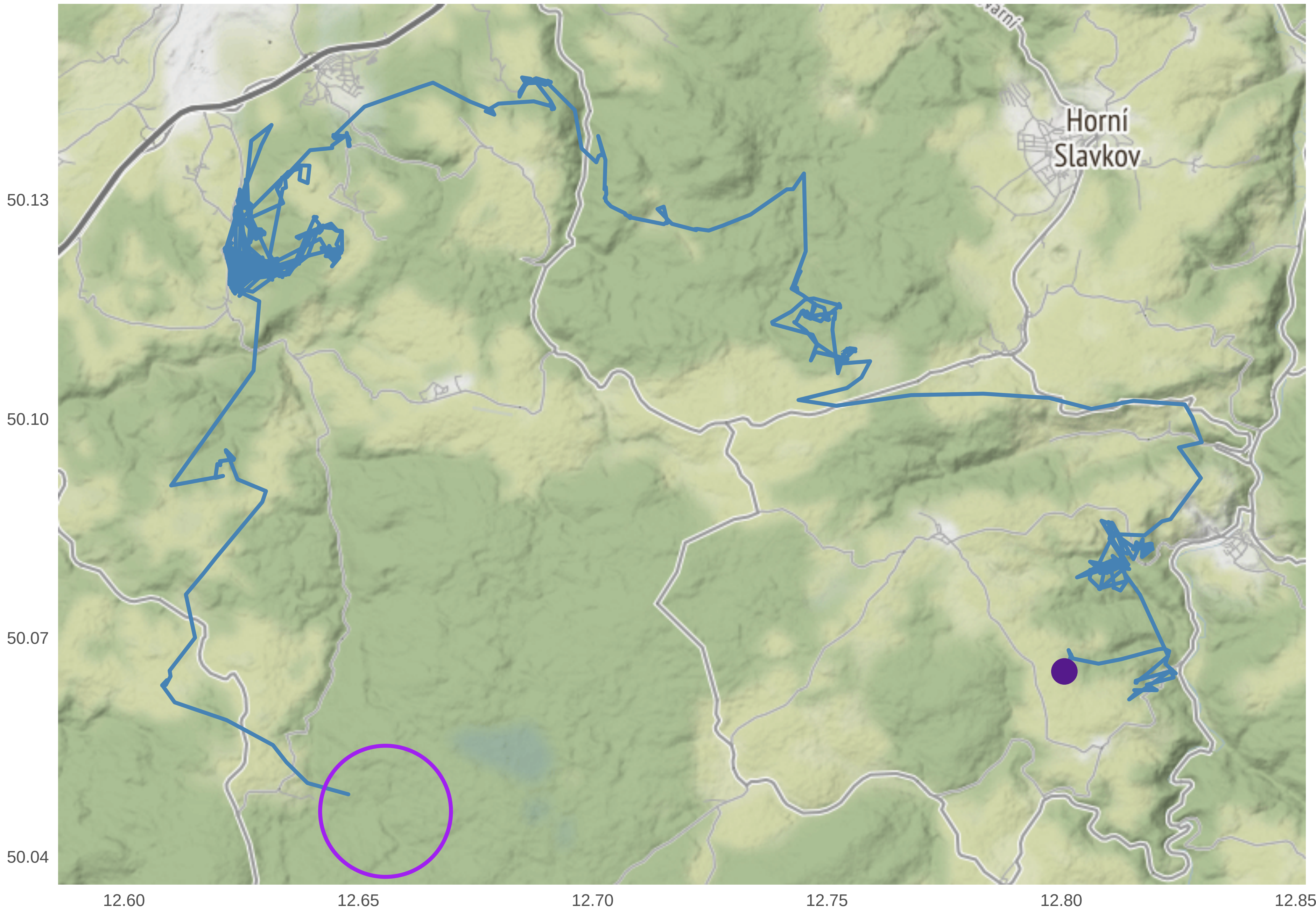

132.1, site: Kladska

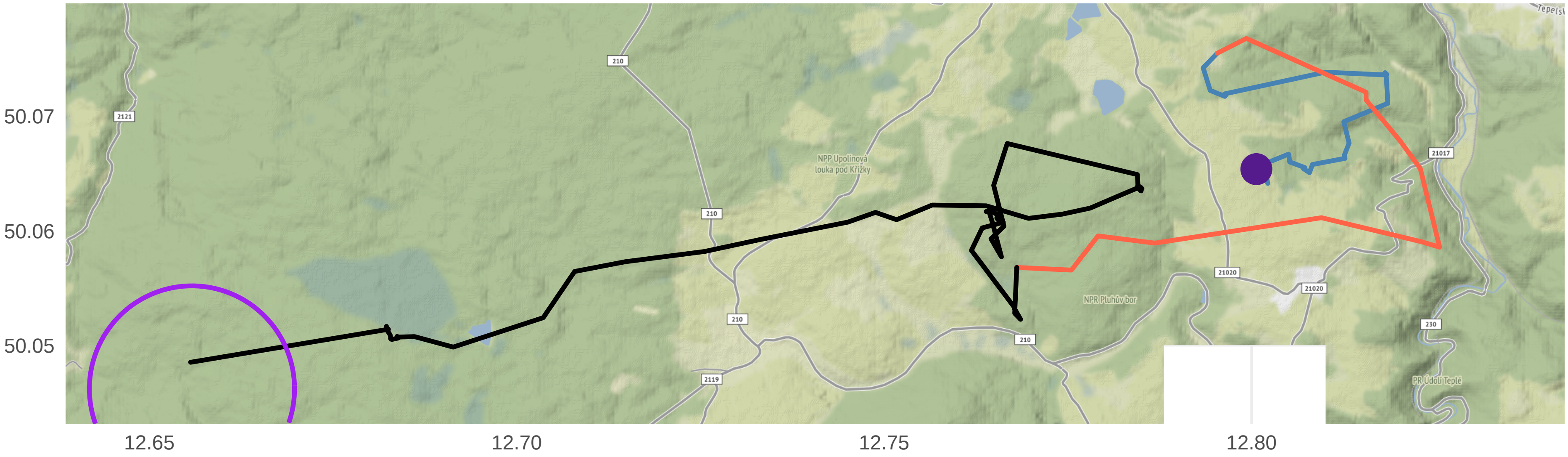

133.1, site: Doupov

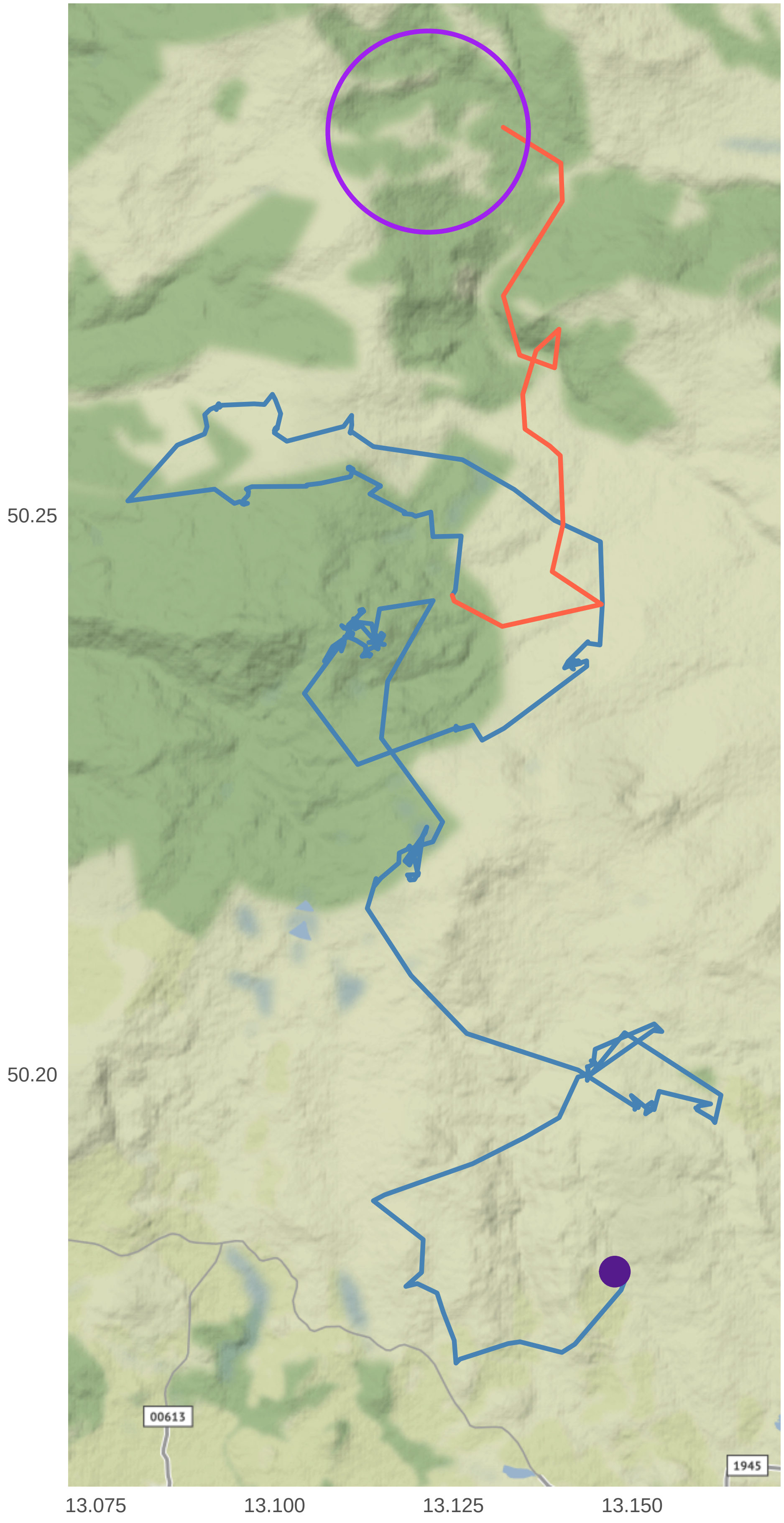

135.1, site: Doupov

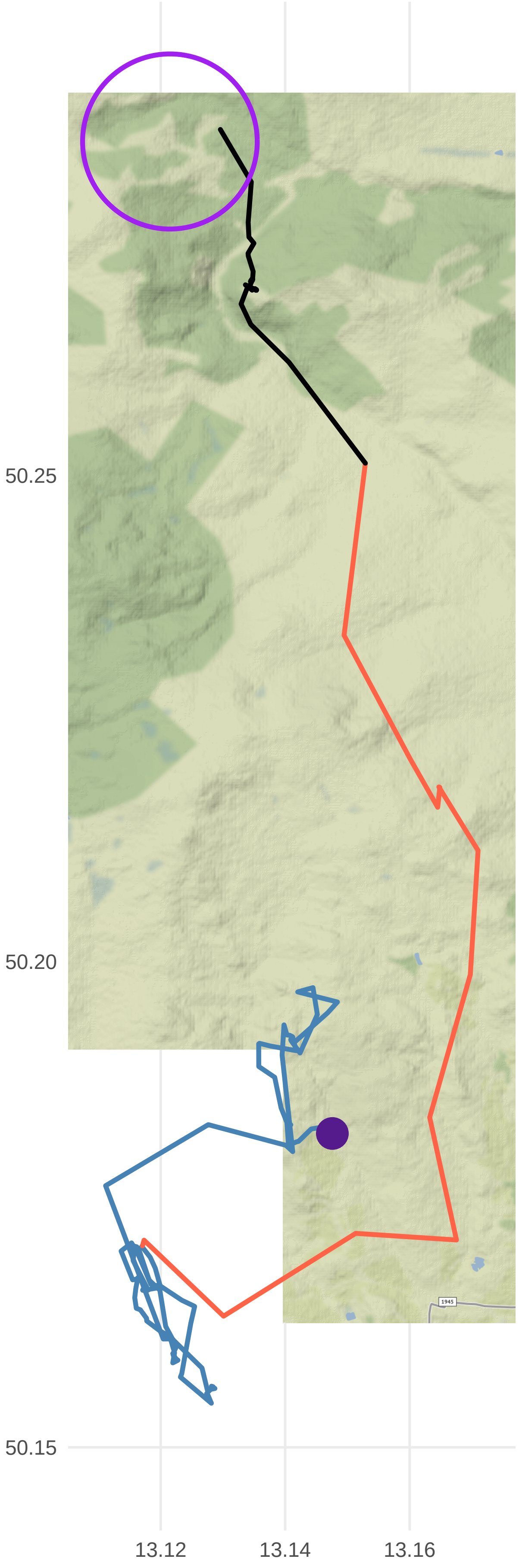

135.2, site: Doupov

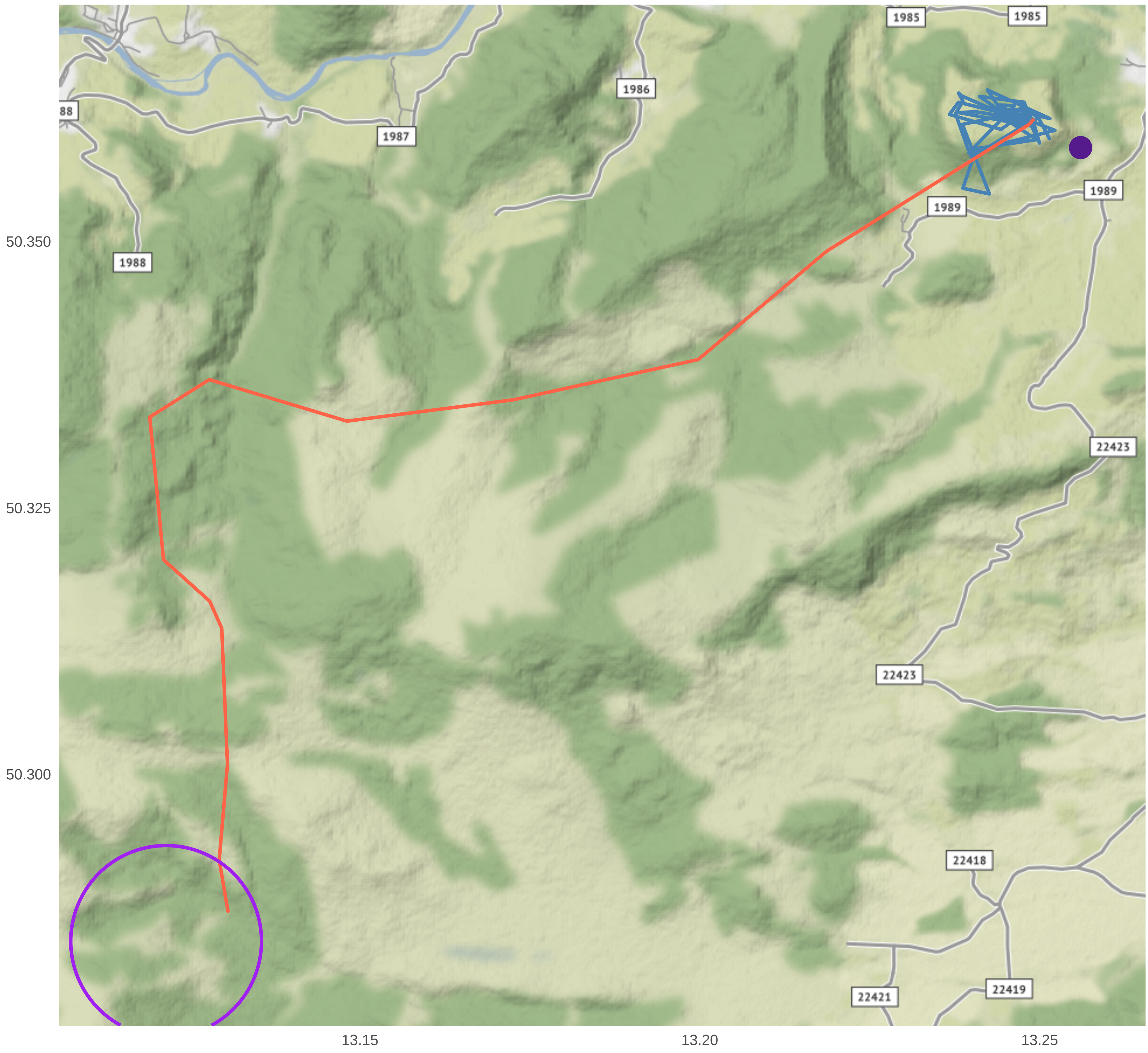

137.1, site: Doupov

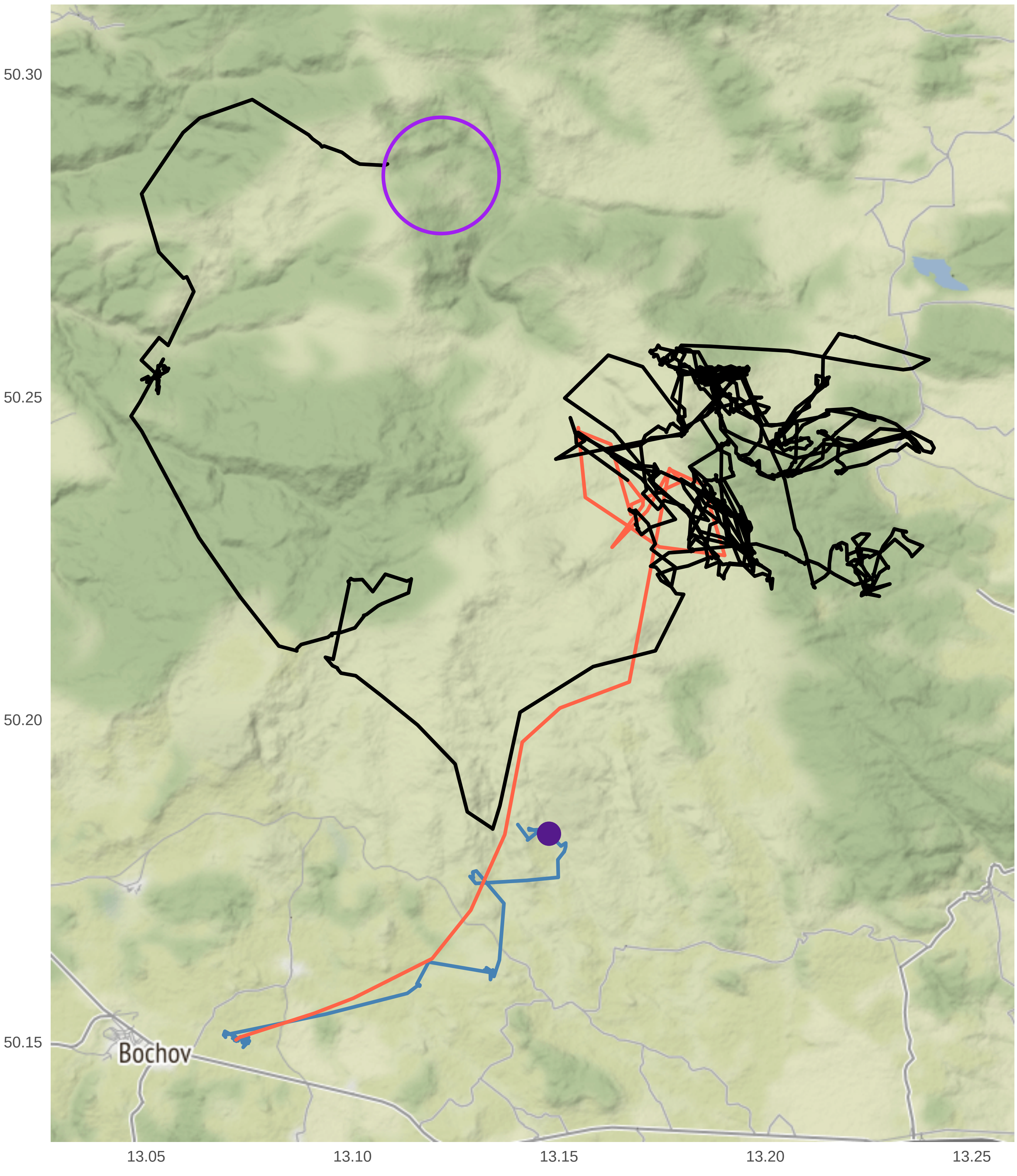

137.2, site: Doupov

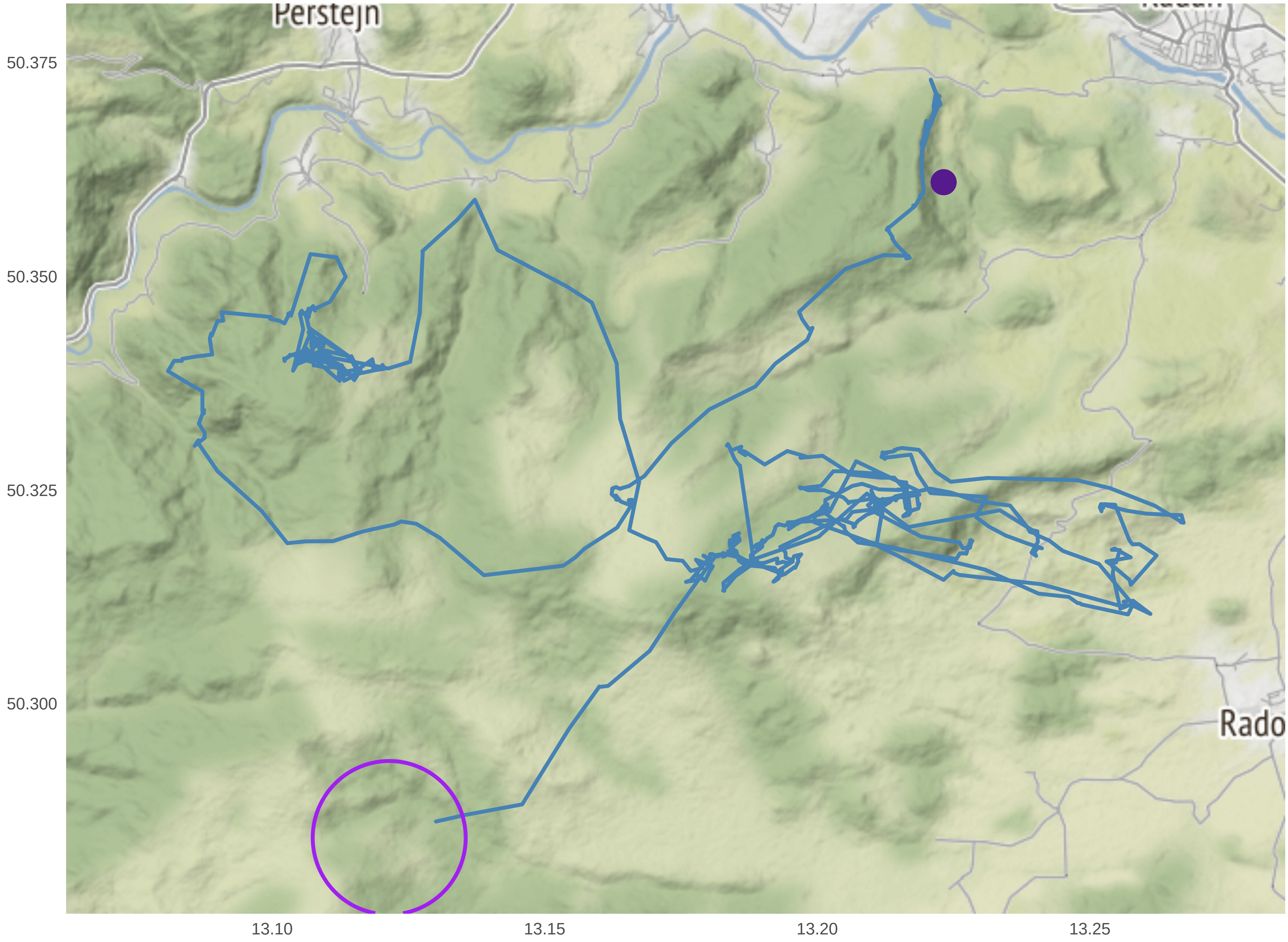

144.1, site: Kladska

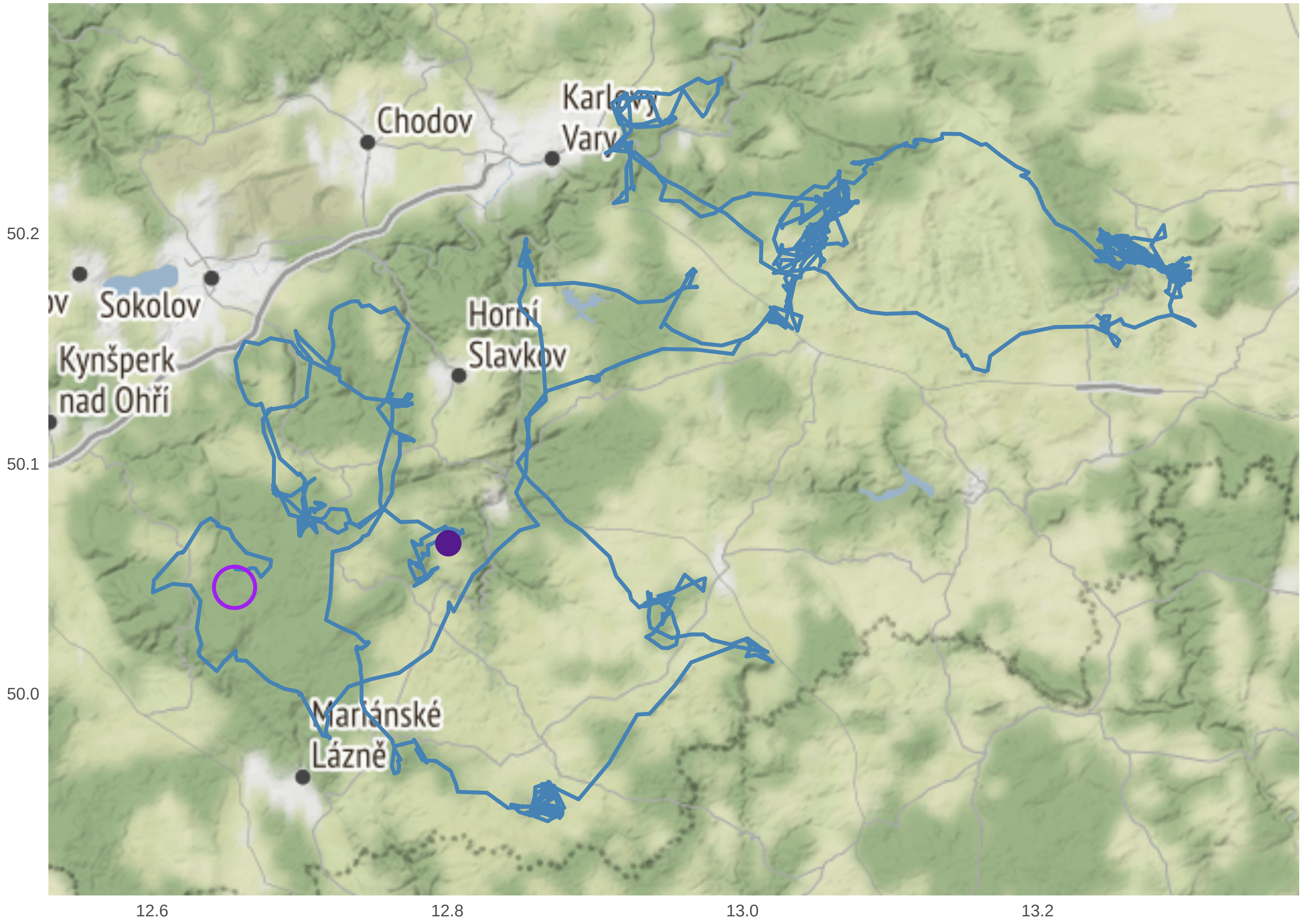

145.1, site: Kladska

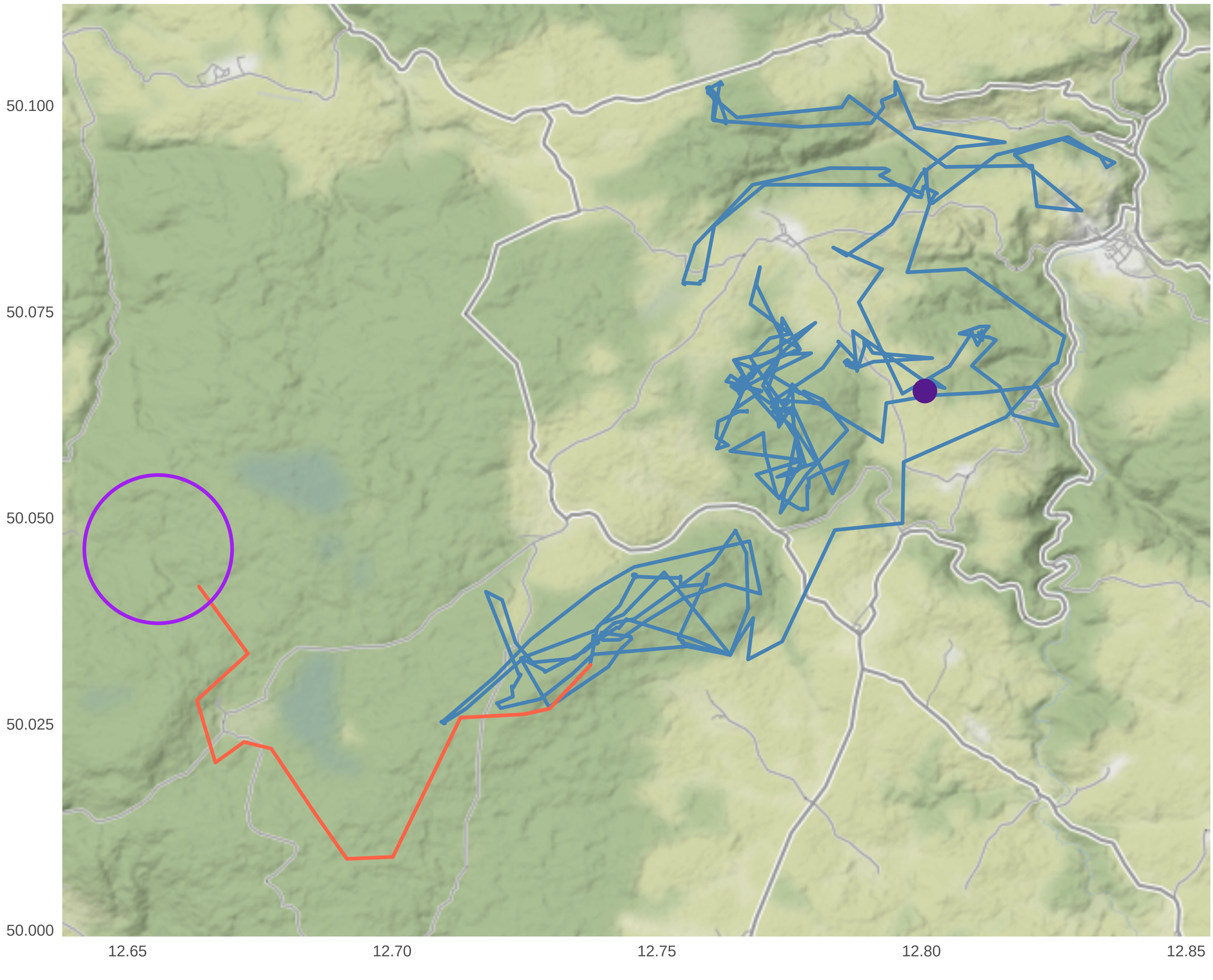

149.1, site: Kladska

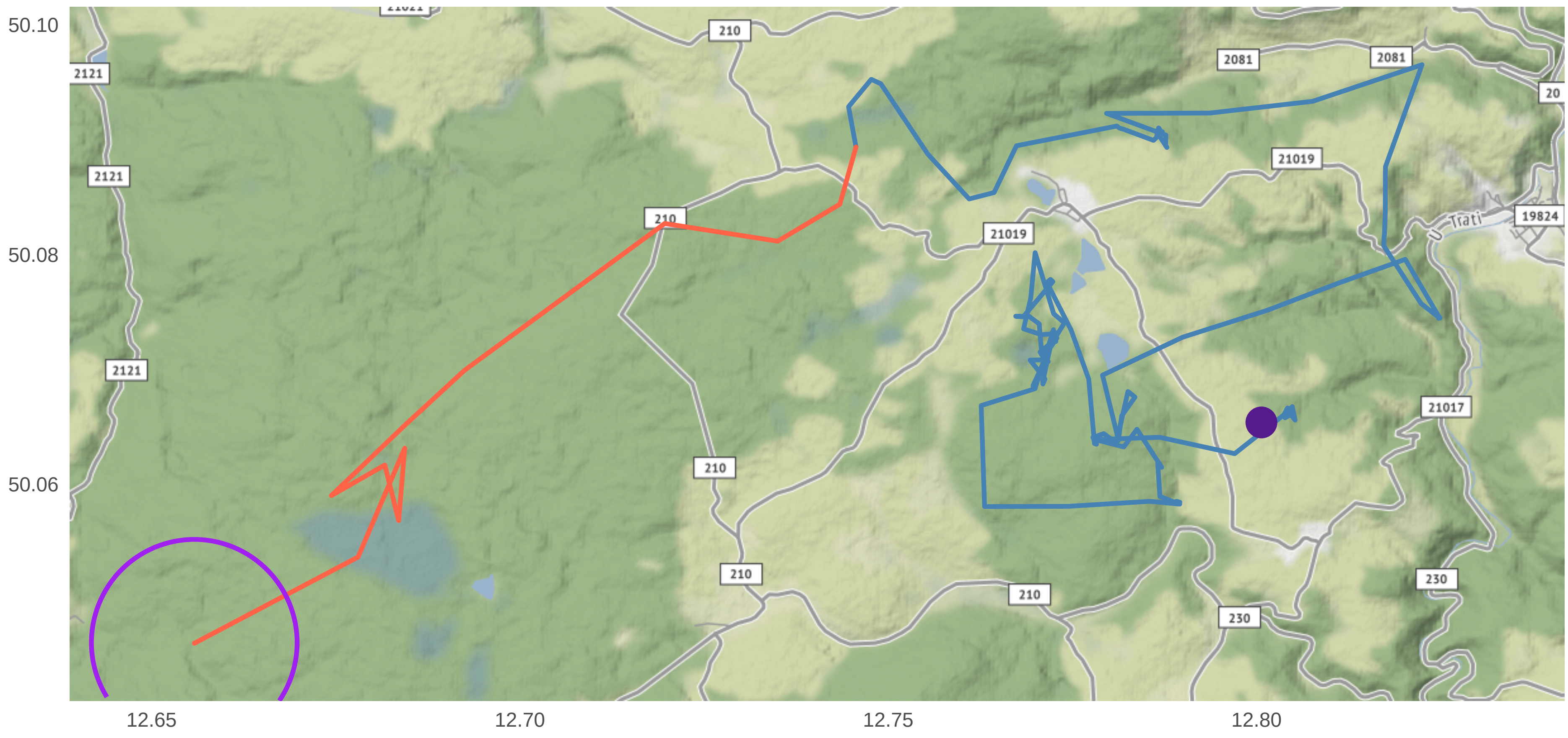

151.1, site: Doupov

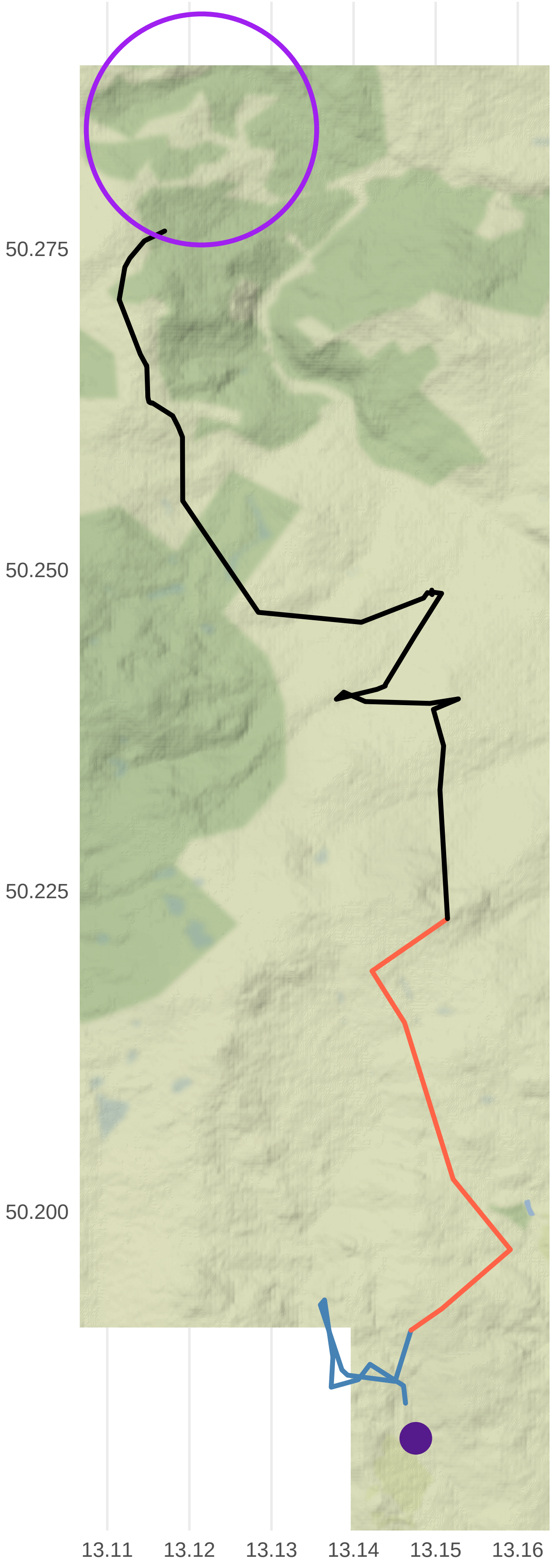

96.1, site: Doupov

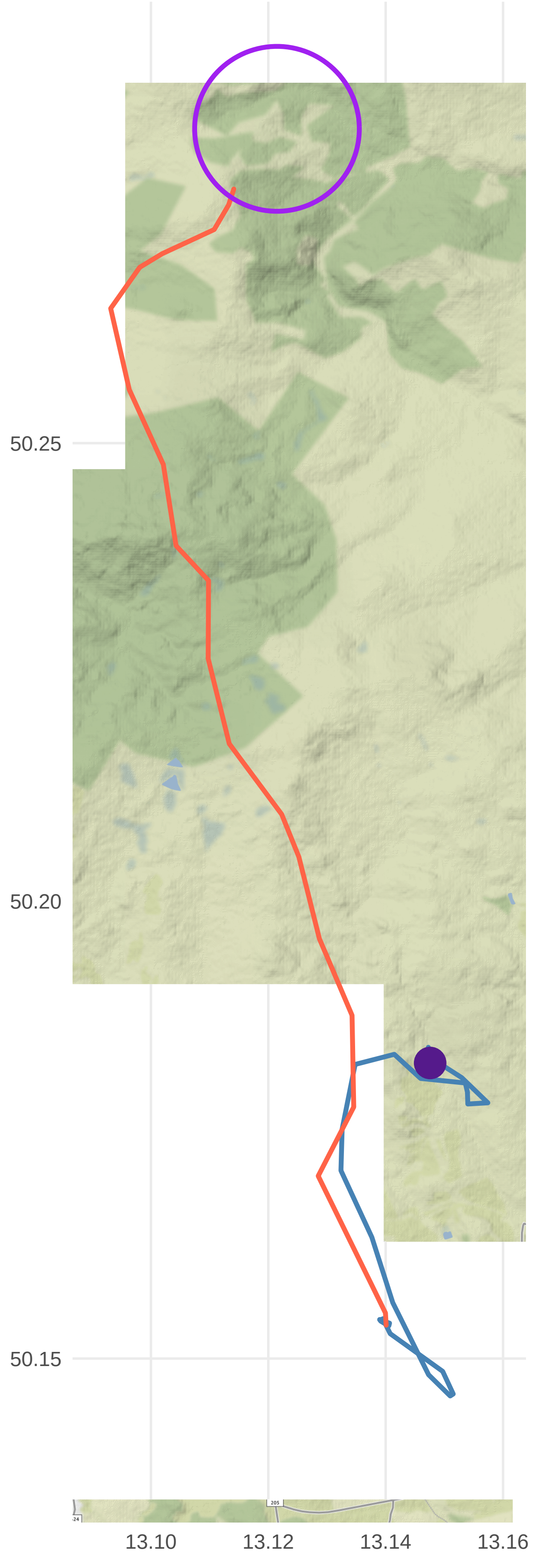

96.2, site: Doupov

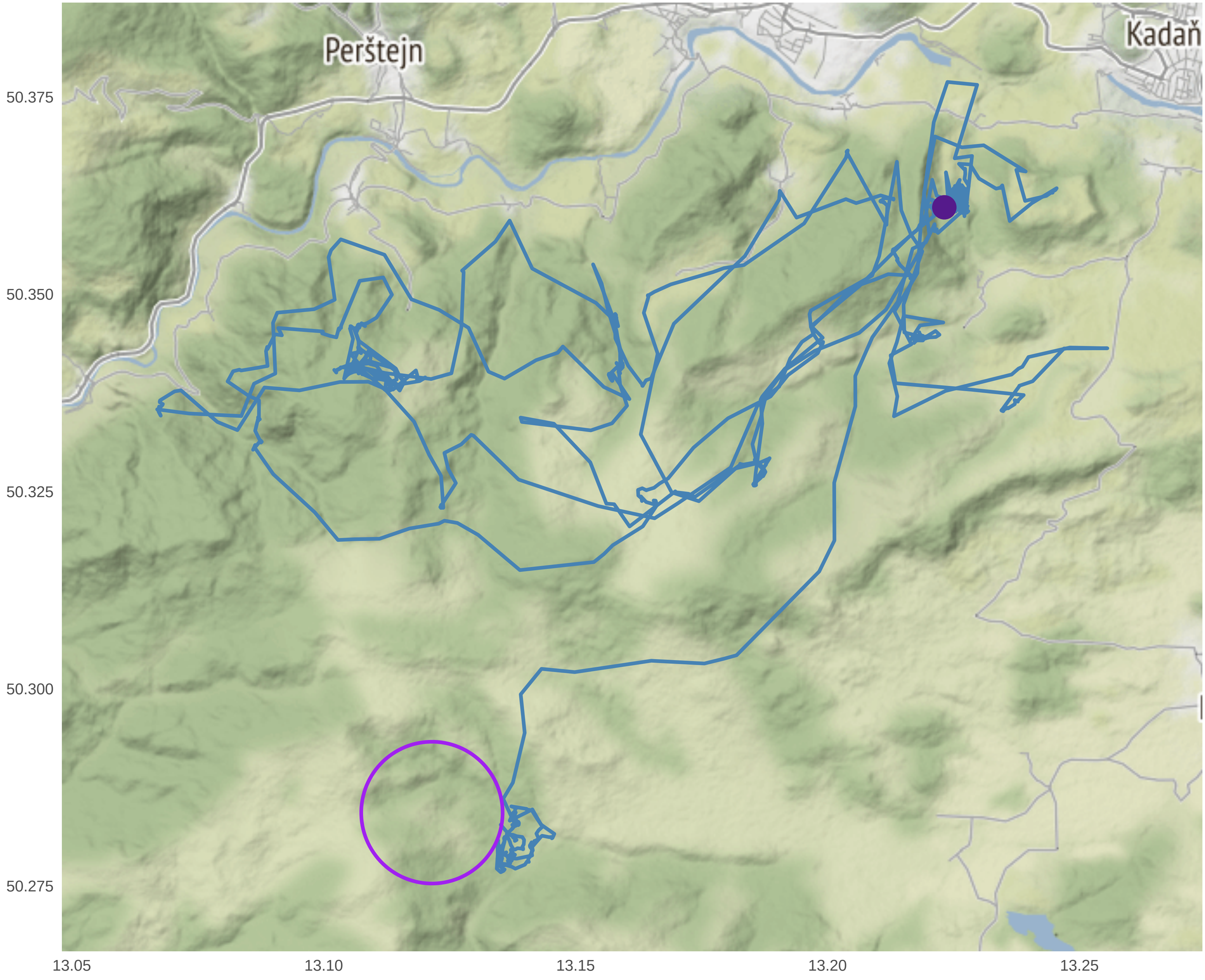

Supplement: Supplementary file 4 — Supplementary Information 4. [file 41598_2024_56951_MOESM4_ESM.pdf]
